# Proteome-wide association study and functional validation identify novel protein markers for pancreatic ductal adenocarcinoma

**DOI:** 10.1093/gigascience/giae012

**Published:** 2024-04-12

**Authors:** Jingjing Zhu, Ke Wu, Shuai Liu, Alexandra Masca, Hua Zhong, Tai Yang, Dalia H Ghoneim, Praveen Surendran, Tanxin Liu, Qizhi Yao, Tao Liu, Sarah Fahle, Adam Butterworth, Md Ashad Alam, Jaydutt V Vadgama, Youping Deng, Hong-Wen Deng, Chong Wu, Yong Wu, Lang Wu

**Affiliations:** Department of Quantitative Health Sciences, John A. Burns School of Medicine, University of Hawaiʻi at Mānoa, Honolulu, HI 96813, USA; Division of Cancer Research and Training, Department of Internal Medicine, Charles R. Drew University of Medicine and Science, David Geffen UCLA School of Medicine and UCLA Jonsson Comprehensive Cancer Center, Los Angeles, CA 90095, USA; Cancer Epidemiology Division, Population Sciences in the Pacific Program, University of Hawaiʻi Cancer Center, University of Hawaiʻi at Mānoa, Honolulu, HI 96813, USA; Cancer Epidemiology Division, Population Sciences in the Pacific Program, University of Hawaiʻi Cancer Center, University of Hawaiʻi at Mānoa, Honolulu, HI 96813, USA; Cancer Epidemiology Division, Population Sciences in the Pacific Program, University of Hawaiʻi Cancer Center, University of Hawaiʻi at Mānoa, Honolulu, HI 96813, USA; Department of Biostatistics, University of Michigan–Ann Arbor, Ann Arbor, MI 48109, USA; Cancer Epidemiology Division, Population Sciences in the Pacific Program, University of Hawaiʻi Cancer Center, University of Hawaiʻi at Mānoa, Honolulu, HI 96813, USA; MRC/BHF Cardiovascular Epidemiology Unit, Department of Public Health and Primary Care, University of Cambridge, Cambridge CB2 0SR, UK; Department of Epidemiology, Johns Hopkins Bloomberg School of Public Health, Baltimore, MD 21205, USA; Division of Surgical Oncology, Michael E. DeBakey Department of Surgery, Baylor College of Medicine, Houston, TX 77030, USA; Center for Translational Research on Inflammatory Diseases (CTRID), Michael E. DeBakey VA Medical Center, Houston, TX 77030, USA; Biological Sciences Division, Pacific Northwest National Laboratory, Richland, WA 99354, USA; MRC/BHF Cardiovascular Epidemiology Unit, Department of Public Health and Primary Care, University of Cambridge, Cambridge CB2 0SR, UK; MRC/BHF Cardiovascular Epidemiology Unit, Department of Public Health and Primary Care, University of Cambridge, Cambridge CB2 0SR, UK; NIHR Blood and Transplant Research Unit in Donor Health and Genomics, Department of Public Health and Primary Care, University of Cambridge, Cambridge CB2 0SR, UK; Tulane Center for Biomedical Informatics and Genomics, Division of Biomedical Informatics and Genomics, Deming Department of Medicine, Tulane University, New Orleans, LA 70112, USA; Division of Cancer Research and Training, Department of Internal Medicine, Charles R. Drew University of Medicine and Science, David Geffen UCLA School of Medicine and UCLA Jonsson Comprehensive Cancer Center, Los Angeles, CA 90095, USA; Department of Quantitative Health Sciences, John A. Burns School of Medicine, University of Hawaiʻi at Mānoa, Honolulu, HI 96813, USA; Tulane Center for Biomedical Informatics and Genomics, Division of Biomedical Informatics and Genomics, Deming Department of Medicine, Tulane University, New Orleans, LA 70112, USA; Department of Biostatistics, The University of Texas MD Anderson Cancer Center, Houston, TX 77030, USA; Division of Cancer Research and Training, Department of Internal Medicine, Charles R. Drew University of Medicine and Science, David Geffen UCLA School of Medicine and UCLA Jonsson Comprehensive Cancer Center, Los Angeles, CA 90095, USA; Cancer Epidemiology Division, Population Sciences in the Pacific Program, University of Hawaiʻi Cancer Center, University of Hawaiʻi at Mānoa, Honolulu, HI 96813, USA

**Keywords:** biomarkers, protein, genetics, pancreatic cancer, risk

## Abstract

Pancreatic ductal adenocarcinoma (PDAC) remains a lethal malignancy, largely due to the paucity of reliable biomarkers for early detection and therapeutic targeting. Existing blood protein biomarkers for PDAC often suffer from replicability issues, arising from inherent limitations such as unmeasured confounding factors in conventional epidemiologic study designs. To circumvent these limitations, we use genetic instruments to identify proteins with genetically predicted levels to be associated with PDAC risk. Leveraging genome and plasma proteome data from the INTERVAL study, we established and validated models to predict protein levels using genetic variants. By examining 8,275 PDAC cases and 6,723 controls, we identified 40 associated proteins, of which 16 are novel. Functionally validating these candidates by focusing on 2 selected novel protein-encoding genes, *GOLM1* and *B4GALT1*, we demonstrated their pivotal roles in driving PDAC cell proliferation, migration, and invasion. Furthermore, we also identified potential drug repurposing opportunities for treating PDAC.

**Significance:**

PDAC is a notoriously difficult-to-treat malignancy, and our limited understanding of causal protein markers hampers progress in developing effective early detection strategies and treatments. Our study identifies novel causal proteins using genetic instruments and subsequently functionally validates selected novel proteins. This dual approach enhances our understanding of PDAC etiology and potentially opens new avenues for therapeutic interventions.

## Introduction

Pancreatic cancer is the seventh leading cause of cancer deaths in industrialized countries with pancreatic ductal adenocarcinoma (PDAC), making up over 90% of pancreatic cancer cases [1]. According to GLOBOCAN 2020 cancer statistics, pancreatic cancer is the 14th most common cancer type with 495,773 new cases in 2020. There were almost the same number of deaths caused by pancreatic cancer (466,003 deaths) in 2020, accounting for 4.7% of all cancer-related deaths [2]. Owing to its often asymptomatic or nonspecific symptoms during early stages, most patients are usually diagnosed in advanced stages. This results in 80–90% of pancreatic tumors being unresectable upon diagnosis, leading to a dismal prognosis: a mere 9% five-year survival rate after diagnosis [1]. Given these dire statistics, there is an urgent need to identify effective biomarkers for screening or early detection in high-risk populations. Equally crucial is the development of improved therapeutic strategies to improve PDAC outcome.

Currently, serum cancer antigen (CA) 19–9 is the only diagnostic biomarker for pancreatic cancer approved by the US Food and Drug Administration. However, elevated levels of CA 19–9 are related to other conditions, and its performance as a diagnostic tool for pancreatic cancer is far from ideal [3]: it has a poor positive predictive value (0.5–0.9%), along with restricted specificity (82–90%) and sensitivity (79–81%). Previous studies have also reported several other circulating blood protein biomarkers that are potentially associated with pancreatic cancer risk, such as CA242, PIVKA-II, and PAM4 [4–7]. However, results from existing studies often involving small sample sizes and findings are inconsistent. It is well known that the conventional epidemiologic study design measuring levels of proteins directly may be subject to selection bias and residual or unmeasured confounding, which could also contribute to the inconsistent findings in the existing literature.

An alternative design of using genetic instruments may decrease many limitations of existing studies, due to the nature of random assortment of alleles from parents to offspring during gamete formation [8, 9]. Inspired by transcriptome-wide association study (TWAS), one may build comprehensive genetic prediction models for each protein to capture the prediction value of multiple single-nucleotide polymorphisms (SNPs). Unlike conventional TWAS type of methods, which typically focus solely on *cis*-acting variants, our study enhanced statistical power by integrating both *cis*- and *trans*-acting elements into our genetic prediction models. Furthermore, as TWAS or proteome-wide association study (PWAS) results imply causality under stringent valid instrumental variable assumptions, we further functionally validated two novel proteins.

In the current study, we applied such a study design to identify novel proteins associated with PDAC risk. To our knowledge, this is the first large-scale PWAS using comprehensive protein genetic prediction models as instruments to assess the associations between genetically predicted blood concentrations of proteins and PDAC risk. We used data for 8,275 cases and 6,723 controls of European descent from the Pancreatic Cancer Cohort Consortium (PanScan) and the Pancreatic Cancer Case-Control Consortium (PanC4). Beyond identifying novel proteins, we functionally validated 2 of them. Moreover, we generated a list of drugs targeting the identified proteins that may serve as candidates for drug repurposing of PDAC.

## Methods

### Protein genetic prediction model development and validation

We leveraged the genome and plasma proteome data of healthy European subjects included in the INTERVAL study to establish (subcohort 1) and validate (subcohort 2) protein genetic prediction models. The details of the INTERVAL study data have been published previously [10–14]. Briefly, participants were generally healthy. The SOMAscan assay was used to collect the relative levels of 3,620 plasma proteins or complexes. Quality control (QC) was performed at both the sample and SOMAmer level. Approximately ∼830,000 genetic variants were measured on the Affymetrix Axiom UK Biobank genotyping array. Standard sample and variant QC were conducted. SNPs were phased using SHAPEIT3 and imputed using a combined 1000 Genomes Phase 3-UK10K reference panel, which resulted in over 87 million imputed variants. The SNPs were further filtered using criteria of (i) imputation quality of at least 0.7, (ii) minor allele count of at least 5%, (iii) Hardy–Weinberg equilibrium (HWE) *P ≥* 5 × 10^−6^, (iv) missing rates <5%, and (v) presenting in the 1000 Genome Project data for European populations. Overall there were 4,662,360 variants passing these criteria.

In subcohort 1 (*N* = 2,481), as described elsewhere [10], protein concentrations were log transformed and adjusted for age, sex, duration between blood draw and processing, and the top 3 principal components. For the rank-inverse normalized residuals of each protein, we followed the TWAS/FUSION framework to establish prediction models, using nearby variants (within 100 kb) of potentially associated SNPs as candidate predictors [15]. A false discovery rate (FDR) <0.05 was used to determine potentially associated SNPs in *cis* regions (within 1 Mb of the transcriptional start site [TSS] of the gene encoding the target protein of interest), and *P* ≤ 5 × 10^−8^ was used to determine potentially associated SNPs in *trans* regions. We only included strand unambiguous SNPs. Four methods of best linear unbiased predictor (blup), elastic net, least absolute shrinkage and selection operator (LASSO), and top1 were used to develop the models. For each protein of interest, the model showing the most significant cross-validation *P* value among those developed using the 4 methods was selected. *R*^2^ ≥ 0.01 was used as the threshold for selecting satisfactory prediction models, which is commonly used in relevant omics integration studies [16–30]. For protein prediction models with *R*^2^ ≥ 0.01, external validation was conducted using genetic and protein data of subcohort 2 (*N* = 820). Briefly, predicted protein expression levels were estimated by applying the developed protein prediction models to the genetic data, which were further compared with the measured levels for each protein of interest. Proteins with a model prediction *R*^2^ of *≥*0.01 in subcohort 1 and a correlation coefficient of *≥*0.1 in subcohort 2 were selected for association analysis with PDAC risk. We also estimated the genetic heritability of plasma proteins (the proportion of the variation of protein levels that could be explained by potential predictors) using GCTA [31]. We compared the heritability of plasma proteins when using *cis* + *trans* SNPs versus only *cis* SNPs to assess whether it could capture more heritability when involving *trans* SNPs.

### Examine associations of genetically predicted protein levels with PDAC risk

To investigate the associations between genetically predicted circulating protein levels and PDAC risk, the validated protein genetic prediction models were applied to the summary statistics from a large genome-wide association study (GWAS) of PDAC risk. In the present work, we used data from a GWAS conducted in the PanScan and PanC4 consortia downloaded from the database of Genotypes and Phenotypes (dbGaP), including 8,275 PDAC cases and 6,723 controls of European ancestry. Detailed information on this dataset has been included elsewhere [17, 20, 32]. Briefly, 4 GWASs (PanScan I, PanScan II, PanScan III, and PanC4) were genotyped using the Illumina HumanHap550,610-Quad, OmniExpress, and OmniExpressExome arrays, respectively. Standard QC procedures were performed according to the consortia guidelines [32]. Study participants who were related to each other, had sex discordance, had genetic ancestry other than Europeans, had a low call rate (less than 98% and 94% in PanC4 and PanScan, respectively), or had missing information on age or sex were excluded. Duplicated SNPs and those with a high missing call rate (at least 2% and 6% in PanC4 and PanScan, respectively) or with violations of HWE (*P* < 1 × 10^−4^ and *P* < 1 × 10^−7^ in PanC4 and PanScan, respectively) were also removed. Regarding SNP data from PanC4, those with minor allele frequency <0.005, with more than 2 discordant calls in duplicate samples, with more than 1 Mendelian error in HapMap control trios, and with a sex difference in allele frequency >0.2 or in heterozygosity >0.3 for autosomes/XY in European descendants were further removed. We performed genotype imputation using Minimac3 after prephasing with SHAPEIT from a reference panel of the Haplotype Reference Consortium (r1.1 2016) [33, 34]. We retained imputed SNPs with an imputation quality of ≥0.3. The associations between individual genetic variants and PDAC risk were further estimated adjusting for age, sex, and top principal components. The TWAS/FUSION framework was used to assess the protein–PDAC risk associations by leveraging correlations between variants included in the prediction models based on the phase III 1000 Genomes Project data for European populations [15]. We calculated the PWAS test statistic *z*-score = *w“Z*/(*w”*Σ_s,s_*w*)^1/2^, where the *Z* is a vector of standardized effect sizes of SNPs for a given protein (Wald *z*-scores), *w* is a vector of prediction weights for the abundance feature of the protein being tested, and the Σ_s,s_ is the linkage disequilibrium (LD) matrix of the SNPs estimated from the 1000 Genomes Project as the LD reference panel. We used the FDR-corrected *P* value threshold of ≤0.05 to determine significant associations between genetically predicted protein concentrations and risk of PDAC.

### Robustness analyses

To further examine whether the identified significant associations from the main analyses may be robust to different strategies, 3 alternative strategies were used to test these proteins under different scenarios. First, we established prediction models using the bslmm method embedded in TWAS/FUSION software. This method was not enabled by the default parameter due to the intensive Markov chain Monte Carlo (MCMC) computation, although bslmm has some advantages and might increase prediction accuracy in some conditions. Second, we pruned the highly correlated SNPs, and only SNPs weakly correlated with each other were used as potential predictors. In the current analysis, we pruned SNPs using pruning parameters *r*^2^ = 0.1 and distance = 250 kb. Third, we assessed the robustness of the significant association results by examining different *P* value cutoffs for selecting informative *trans* regions (*P* < 5 × 10^−7^, *P* < 5 × 10^−9^, and *P* < 5 × 10^−10^) as candidate predictors for model building. The association results with a nominal *P* < 0.05 and consistent effect direction were considered replicated.

### Somatic variants of genes encoding associated proteins

For each of the genes encoding the proteins that are identified to be associated with PDAC risk, we evaluated potentially deleterious somatic level mutations in 150 patients with PDAC included in The Cancer Genome Atlas (TCGA). The potentially deleterious somatic variants include missense mutations, splice site mutations, nonstop mutations, nonsense mutations, frameshift mutations, in-frame mutations, and translation start site mutations.

The somatic-level genetic changes were called using MuTect2 [35] and deposited to the TCGA data portal. The enrichment of the proportion of assessed genes containing such somatic-level genetic events compared with the proportion of all protein-coding genes across the genome was evaluated using socscistatistics online website [36].

### Ingenuity Pathway Analysis and protein–protein interaction analysis

To further assess whether genes encoding the identified PDAC-associated proteins are enriched in specific pathways, molecular and cellular functions, and networks, we performed the enrichment analysis using Ingenuity Pathway Analysis (IPA) software [37]. The “enrichment” score (Fisher exact test *P* value) that measures overlap of observed and predicted regulated gene sets was generated for each of the tested gene sets. The most significant pathways and functions with an enrichment *P* value less than 0.05 were reported. We also built a protein–protein interaction (PPI) network using STRING database version 11.5 with a 0.400 confidence level [38]. The STRING database integrates different curated databases containing information on known and predicted functional protein–protein associations.

### Drug repurposing analysis

For the identified proteins, we further assessed whether there is any evidence supporting their potential roles in PDAC by using the OpenTargets [39]. Focusing on those showing a potential relevance, we further mined evidence of their targeting drugs using the DrugBank [40] database. We also conducted molecular docking analysis for the identified proteins and corresponding candidate drug agents [41]. Specifically, we downloaded the 3-dimensional structure of targeted proteins from the Protein Data Bank (PDB) [42] with source code 1CPB, 3CDZ, 1IGR, 3DFK, 5NO06, and drug agents from the PubChem database [43]. We further worked out molecular docking between each of the proteins and the corresponding meta-drug agents to calculate the binding affinity scores (kcal/mol) for each pair of proteins and drugs.

### *In vitro* functional validation of genes encoding selected associated novel proteins

#### Cell lines and culture condition

Human pancreatic cancer cell lines PANC-1 and SU.86.86 were obtained from ATCC (American Type Culture Collection). All cells were cultured *in vitro* in Dulbecco’s modified Eagle medium (DMEM) high-glucose medium (Gibco) supplemented with 10% (v/v) fetal bovine serum (FBS) (Gibco). Cells were incubated at 37°C with 5% CO_2_.

#### Gene expression and survival analysis with TCGA database

The examination of *GOLM1* and *B4GALT1* gene expressions in pancreatic adenocarcinoma (PAAD) was conducted using the Gene Expression Profiling Interactive Analysis (GEPIA). The platform, accessible at [44], facilitated analysis with a dataset consisting of 179 tumor samples and 171 normal controls. The focus of survival analysis was exclusively on PAAD, leveraging TCGA data through the GEPIA web server.

Customized gene selection, normalization, and survival methodologies were implemented to suit the unique characteristics of PAAD. Cohort thresholds were defined, restricting dataset selection to PAAD, and survival plots were generated. These measures were designed to precisely identify the correlation between gene expression and survival outcomes specific to this type of cancer.

#### Western blotting

Post 72-hour silencing, we processed control, B4GALT1-silenced, and GOLM1-silenced cells for Western blotting. Cells were lysed using RIPA buffer, and equal protein amounts were separated on 10% or 12% SDS polyacrylamide gels, then transferred onto PVDF membranes. To prevent nonspecific antibody binding, membranes were blocked with 5% milk in TBS with 0.1% Tween for an hour. They were then probed with anti-B4GALT1, anti-GOLM1, and anti-GAPDH antibodies, followed by their respective horseradish peroxidase–conjugated secondary antibodies. Signal detection was performed using Pierce ECL Western Blotting Substrate, and images were captured and analyzed using Odyssey FC and ImageStudio software.

#### Quantitative real-time PCR

Total RNA was extracted from cells using TRNzol reagent according to the manufacturer’s protocol. The concentration of RNA was determined using a UV spectrophotometer. Subsequently, 2 mg total RNA was reverse transcribed into complementary DNA (cDNA) using the iScript cDNA Synthesis Kit. Quantitative PCR (qPCR) analysis was performed on the CFX96 Real-Time PCR Detection System using the iTaq Universal SYBR Green Supermix. The aim was to detect the expression levels of 3 genes: B4GALT1, GOLM1, and GAPDH messenger RNAs (mRNAs). Specific primer pairs were used for each gene. For B4GALT1, the forward sequence was GTATTTTGGAGGTGTCTCTGCTC, and the reverse sequence was GGGCGAGATATAGACATGCCTC. For GOLM1, the forward sequence was ATCACCACAGGTGAGAGGCTCA, and the reverse sequence was ACTTCCTCTCCAGGTTGGTCTG. For the housekeeping gene GAPDH, the forward sequence was GTCTCCTCTGACTTCAACAGCG, and the reverse sequence was ACCACCCTGTTGCTGTAGCCAA. During the qPCR analysis, melting curves were generated to detect primer–dimer formation and confirm the specificity of the gene-specific peaks for each target. To ensure accurate quantification, the expression data were normalized to the amount of GAPDH mRNA expressed.

#### Transfection of small interfering RNA

The transfection of small interfering RNA (siRNA) was performed using specific human siRNAs targeting GOLM1 (SASI_Hs01_00,223,155), B4GALT1 (SASI_Hs01_00,080,445), and the MISSION siRNA universal negative control, all of which were obtained from Sigma-Aldrich. Cells were seeded in 6-well plates at a density of 1.5 × 10^5^ cells per well and subsequently transfected with the siRNAs at a concentration of 40 nM. The transfection procedure utilized the Lipofectamine 2000 reagent (Invitrogen) following the manufacturer’s recommended guidelines. Gene silencing at both mRNA and protein levels was typically observed 72 hours posttransfection. As such, the cells were collected and subjected to assays at the 72-hour time point to assess the efficacy of gene silencing.

#### Cell proliferation assay

To observe cell proliferation, cells were transfected with mock siRNA, siGOLM1, and siB4GALT1 (40 nM). At 24 hours after transfection, the cells were trypsinized and seeded into 96-well plates (Corning) at a density of 5,000 cells/well in 200 µL media. The plates were incubated in a 37°C humidified incubator. Cell proliferation was monitored daily by the [3-(4,5-dimethylthiazol-2-yl)-5-(3-carboxymethoxyphenyl)-2-(4-sulfophenyl)-2H-tetrazolium] (MTS) assay.

#### *In vitro* invasion assay

Cell invasion was assessed following transfection with mock siRNA, siGOLM1, and siB4GALT1 (40 nM). A modified Boyden chamber method was employed. Matrigel (BD Biosciences) was coated on the upper chamber of Transwell inserts (Corning, 8-µm pore size) at a concentration of 300 µg/mL, allowing gel formation for 2 hours at 37°C. Cells (5 × 10^4^) were then suspended in 200 µL serum-free medium and added to the upper chamber. The lower chamber contained 600 µL medium with 10% FBS, acting as a chemoattractant. Following 24 hours of incubation at 37°C, noninvading cells on the upper membrane surface were gently removed using a cotton swab. Cells that invaded the lower membrane surface were fixed with 4% paraformaldehyde and stained with 0.1% crystal violet. Invasion was quantified by counting the stained cells on the underside of the membrane using a light microscope (10 random fields at 200× magnification). All experiments were performed in triplicate to ensure robustness of the findings.

#### Wound scratch assay

After 24 hours of transfection with mock siRNA, siGOLM1, and siB4GALT1, PANC-1 and SU.86.86 cells were cultured in a 96-well plate to form a monolayer. Using BioTek’s AutoScratch Wound Making Tool, straight scratches were carefully created on the cell monolayer to mimic wounds, following the equipment manual’s instructions. Time-lapse images of the scratches were captured at specific intervals (e.g., 0 hours, 12 hours, 24 hours, etc.) using the Cytation 5 Cell Imaging Multi-Mode Reader. Subsequently, image analysis software was employed to quantify the closure of the wounds at each time point. Statistical analysis was performed to compare the wound closure rates at different time points, and the results were presented graphically.

## Results

**Figure 1:**
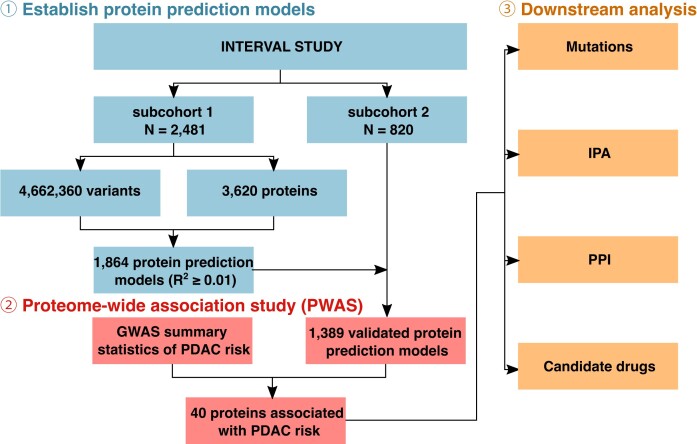
The overall design of this study.

**Figure 2:**
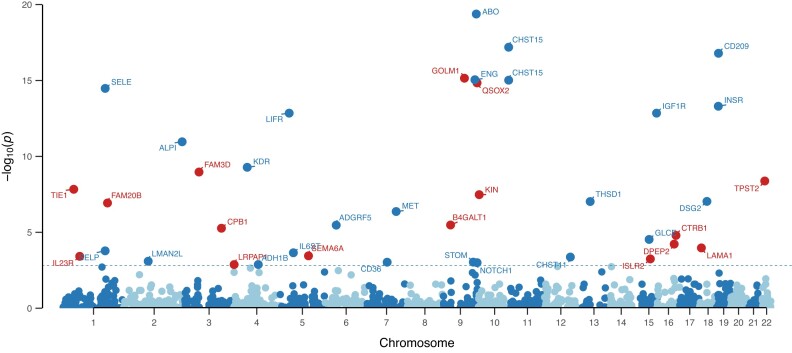
Manhattan plot of 40 identified proteins associated with PDAC risk. Proteins in blue represent those identified in our previous work using pQTL as instruments, and proteins in red represent novel ones identified in the current study.

The overall workflow of this study is shown in Fig. 1. Of the proteins assessed, we were able to develop prediction models for 1,864 proteins with a prediction performance *R*^2^ ≥ 0.01. In the external validation step, 1,389 of them further demonstrated a correlation coefficient of *≥*0.1 for predicted expression and measured expression levels. The heritability of the proteins ranged from 0.001 to 0.87, with an average value of 0.14. Of such proteins, we observed significant associations between genetically predicted expression levels of 40 proteins and PDAC risk at an FDR *P* value of ≤0.05 (Fig. 2, Tables 1 and 2). Of the associated proteins, 16 are novel ones that have not been reported in previous studies (Table 1). Positive associations were observed for 10 of these proteins, and inverse associations were observed for 6 proteins (Table 1). The other 24 associated proteins have been previously reported in our study using pQTL as instruments [45] (Table 2). These include 10 that demonstrated positive associations and 14 that showed inverse associations.

**Table 1:** Novel proteins with genetically predicted concentrations in plasma to be associated with pancreatic cancer risk

Protein	SOMAmer ID	Protein full name	Protein-encoding gene	Region for protein encoding gene	Prediction model method	Heritability	Number of predicting SNPs	Number of predicting SNPs-cis*	Number of predicting SNPs-*trans*	Model internal cross-validation *R*^2^	Model external validation *R*^2^	*z* value^a^	*P* value^a^	FDR *P* value^b^
IL-23 R	IL23R.5088.175.3	Interleukin-23 receptor	*IL23R*	1p31.3	Elastic net	0.06	24	24	0	0.04	0.04	3.55	3.80 × 10^−4^	0.02
sTie-1	TIE1.2844.53.2	Tyrosine-protein kinase receptor tie-1, soluble	*TIE1*	1p34.2	LASSO	0.2	18	7	11	0.22	0.28	5.67	1.46 × 10^−8^	1.22 × 10^−6^
FA20B	FAM20B.7198.197.3	Glycosaminoglycan xylosylkinase	*FAM20B*	1q25.2	LASSO	0.05	8	5	3	0.02	0.04	5.30	1.17 × 10^−7^	7.82 × 10^−6^
FAM3D	FAM3D.13102.1.3	Protein FAM3D	*FAM3D*	3p14.2	Elastic net	0.27	58	16	42	0.37	0.36	6.10	1.07 × 10^−9^	1.02 × 10^−7^
Carboxypeptidase B1	CPB1.6356.3.3	Carboxypeptidase B	*CPB1*	3q24	LASSO	0.07	7	3	4	0.04	0.03	−4.55	5.38 × 10^−6^	3.00 × 10^−4^
RAP	LRPAP1.3640.14.3	alpha-2-macroglobulin receptor-associated protein	*LRPAP1*	4p16.3	Elastic net	0.47	168	23	145	0.27	0.22	3.21	0.001	0.04
Semaphorin-6A	SEMA6A.7945.10.3	Semaphorin-6A	*SEMA6A*	5q23.1	Elastic net	0.11	66	44	22	0.05	0.05	−3.57	3.54 × 10^−4^	0.02
B4GT1	B4GALT1.13381.49.3	Beta-1,4-galactosyltransferase 1	*B4GALT1*	9p21.1	Elastic net	0.10	39	16	23	0.08	0.10	4.65	3.29 × 10^−6^	1.96 × 10^−4^
GOLM1	GOLM1.8983.7.3	Golgi membrane protein 1	*GOLM1*	9q21.33	LASSO	0.11	10	0	10	0.14	0.17	8.07	7.12 × 10^−16^	2.14 × 10^−13^
QSOX2	QSOX2.8397.147.3	Sulfhydryl oxidase 2	*QSOX2*	9q34.3	Elastic net	0.31	28	10	18	0.40	0.40	7.98	1.44 × 10^−15^	2.75 × 10^−13^
KIN17	KIN.14643.27.3	DNA/RNA-binding protein KIN17	*KIN*	10p14	Elastic net	0.08	29	0	29	0.05	0.07	−5.52	3.31 × 10^−8^	2.60 × 10^−6^
ISLR2	ISLR2.13124.20.3	Immunoglobulin superfamily containing leucine-rich repeat protein 2	*ISLR2*	15q24.1	Elastic net	0.17	77	32	45	0.14	0.13	−3.45	5.65 × 10^−4^	0.02
DPEP2	DPEP2.8327.26.3	Dipeptidase 2	*DPEP2*	16q22.1	Elastic net	0.07	36	0	36	0.06	0.05	−4.01	5.97 × 10^−5^	0.003
Chymotrypsin	CTRB1.5671.1.3	Chymotrypsinogen B	*CTRB1*	16q23.1	Elastic net	0.35	85	69	16	0.23	0.24	−4.32	1.59 × 10^−5^	8.50 × 10^−4^
Laminin	LAMA1.LAMB1.LAMC1.2728.62.2	Laminin	*LAMA1, LAMB1, LAMC1*	18p11.31, 7q31.1, 1q25.3	Elastic net	0.09	62	14	48	0.08	0.05	3.88	1.06 × 10^−4^	0.005
TPST2	TPST2.8024.64.3	Protein-tyrosine sulfotransferase 2	*TPST2*	22q12.1	Elastic net	0.08	52	28	24	0.07	0.08	5.88	4.16 × 10^−9^	3.71 × 10^−7^

* SNPs within 1 MB of the protein-encoding gene.

a Associations between genetically predicted protein levels and PDAC risk after adjustment for age, sex, and top 10 principal components.

b FDR *P* value: FDR-adjusted *P* value; associations with an FDR *P* ≤ 0.05 considered statistically significant.

**Table 2:** Previously reported proteins with genetically predicted concentrations in plasma to be associated with pancreatic cancer risk

Protein	SOMAmer ID	Protein full name	Protein-encoding gene	Region for protein encoding gene	Prediction model method	Heritability	Number of predicting SNPs	Number of predicting SNPs-cis*	Number of predicting SNPs-*trans*	Model internal cross-validation *R*^2^	Model external validation *R*^2^	*z* value^a^	*P* value^a^	FDR *P* value^b^
sE-Selectin	SELE.3470.1.2	E-selectin	*SELE*	1q24.2	LASSO	0.30	6	0	6	0.39	0.44	−7.88	3.33 × 10^−15^	5.47 × 10^−13^
P-Selectin	SELP.4154.57.2	P-selectin	*SELP*	1q24.2	LASSO	0.33	11	7	4	0.26	0.27	−3.77	1.66 × 10^−4^	0.008
LMA2L	LMAN2L.8013.9.3	VIP36-like protein	*LMAN2L*	2q11.2	top1	0.04	1	1	0	0.03	0.02	3.35	8.01 × 10^−4^	0.03
Alkaline phosphatase, intestine	ALPI.10463.23.3	Intestinal-type alkaline phosphatase	*ALPI*	2q37.1	LASSO	0.03	8	0	8	0.03	0.06	−6.79	1.09 × 10^−11^	1.21 × 10^−9^
VEGF sR2	KDR.3651.50.5	Vascular endothelial growth factor receptor 2	*KDR*	4q12	Elastic net	0.29	56	18	38	0.18	0.12	−6.21	5.22 × 10^−10^	5.37 × 10^−8^
ADH1B	ADH1B.9834.62.3	Alcohol dehydrogenase 1B	*ADH1B*	4q23	LASSO	0.12	6	0	6	0.08	0.03	3.21	0.001	0.04
LIF sR	LIFR.5837.49.3	Leukemia inhibitory factor receptor	*LIFR*	5p13.1	top1	0.04	1	0	1	0.03	0.02	−7.39	1.42 × 10^−13^	1.73 × 10^−11^
gp130, soluble	IL6ST.2620.4.2	Interleukin-6 receptor subunit beta	*IL6ST*	5q11.2	Elastic net	0.08	51	21	30	0.06	0.05	−3.69	2.22 × 10^−4^	0.01
GP116	ADGRF5.6409.57.3	Adhesion G protein-coupled receptor F5	*ADGRF5*	6p12.3	LASSO	0.42	22	15	7	0.46	0.43	−4.65	3.37 × 10^−6^	1.96 × 10^−4^
CD36 ANTIGEN	CD36.2973.15.2	Platelet glycoprotein 4	*CD36*	7q21.11	top1	0.04	1	0	1	0.03	0.05	3.31	9.25 × 10^−4^	0.03
Met	MET.2837.3.2	Hepatocyte growth factor receptor	*MET*	7q31	blup	0.09	1,668	603	1,065	0.07	0.04	−5.06	4.27 × 10^−7^	2.72 × 10^−5^
STOM	STOM.8261.51.3	Erythrocyte band 7 integral membrane protein	*STOM*	9q33.2	LASSO	0.10	5	0	5	0.11	0.05	3.31	9.18 × 10^−4^	0.03
BGAT	ABO.9253.52.3	Histo-blood group ABO system transferase	*ABO*	9q34.2	blup	0.55	2,473	2,347	126	0.72	0.72	9.18	4.20 × 10^−20^	5.62 × 10^−17^
Notch 1	NOTCH1.5107.7.2	Neurogenic locus notch homolog protein 1	*NOTCH1*	9q34.3	top1	0.02	1	0	1	0.01	0.02	3.29	9.97 × 10^−4^	0.04
Endoglin	ENG.4908.6.1	Endoglin	*ENG*	9q34.11	top1	0.02	1	0	1	0.01	0.01	−8.04	8.93 × 10^−16^	2.14 × 10^−13^
ST4S6	CHST15.4469.78.2	Carbohydrate sulfotransferase 15	*CHST15*	10q26.13	LASSO	0.05	5	1	4	0.05	0.03	−8.62	6.46 × 10^−18^	4.32 × 10^−15^
	CHST15.14097.86.3				LASSO	0.06	9	2	7	0.04	0.02	−8.03	9.60 × 10^−16^	2.14 × 10^−13^
CHSTB	CHST11.7779.86.3	Carbohydrate sulfotransferase 11	*CHST11*	12q23.3	Elastic net	0.15	69	46	23	0.11	0.07	3.52	4.25 × 10^−4^	0.02
THSD1	THSD1.5621.64.3	Thrombospondin type 1 domain-containing protein 1	*THSD1*	13q14.3	Elastic net	0.07	44	27	17	0.04	0.03	−5.34	9.41 × 10^−8^	6.62 × 10^−6^
GLCE	GLCE.7808.5.3	D-glucuronyl C5-epimerase	*GLCE*	15q23	LASSO	0.27	11	6	5	0.36	0.34	4.18	2.94 × 10^−5^	0.002
IGF-I sR	IGF1R.4232.19.2	Insulin-like growth factor 1 receptor	*IGF1R*	15q26.3	top1	0.01	1	0	1	0.01	0.02	−7.39	1.42 × 10^−13^	1.73 × 10^−11^
Desmoglein-2	DSG2.9484.75.3	Desmoglein-2	*DSG2*	18q12.1	Elastic net	0.06	66	44	22	0.04	0.06	5.34	9.18 × 10^−8^	6.62 × 10^−6^
DC-SIGN	CD209.3029.52.2	CD209 antigen	*CD209*	19p13.2	Elastic net	0.30	58	26	32	0.39	0.38	8.52	1.62 × 10^−17^	7.22 × 10^−15^
IR	INSR.3448.13.2	Insulin receptor	*INSR*	19p13.2	LASSO	0.09	7	0	7	0.09	0.12	−7.53	4.98 × 10^−14^	7.40 × 10^−12^

* SNPs within 1 MB of the protein-encoding gene.

a Associations between genetically predicted protein levels and PDAC risk after adjustment for age, sex, and top 10 principal components.

b FDR *P* value: FDR-adjusted *P* value; associations with an FDR *P* ≤ 0.05 considered statistically significant.

For the other proteins that were reported in our previous study using pQTL as instruments [45], while did not show a significant association after FDR correction in the current study (Supplementary Table S1), except for sTie-2, the directions of effect were consistent in the current study compared with those in the published work. Among them, for 8 proteins, their associations were at *P* < 0.05 in the current work using protein genetic prediction models as instruments (Supplementary Table S1).

We compared the heritability of the prediction models established using *cis* + *trans* and *cis*-only predictors strategies. Here, we focused on the 490 models established using both *cis* and *trans* SNPs in the main analysis. The results showed that 250 out of the 490 (51.02%) models have higher estimated heritability with the *cis* + *trans* strategy (Supplementary Table S2), and 215 proteins (43.88%) showed the same estimated heritability between *cis* + *trans* and *cis*-only strategies (Supplementary Table S2). Only 25 proteins (5.10%) showed lower estimated heritability when using the *cis* + *trans* strategy (Supplementary Table S2). These results showed that *trans* SNPs could in general increase heritability of the prediction models.

The robustness analysis showed that all the 40 PDAC-associated proteins had the same effect directions (Supplementary Table S3). A total of 39 proteins could be tested using the bslmm method and 37 out of the 39 (94.87%) could be replicated (except for SEMA6A and CHST11 proteins). When we removed highly correlated SNPs and only weakly correlated SNPs were used for establishing prediction models, a total of 39 prediction models were established. The association results showed that associations of 38 out of the 39 (97.44%) proteins could be replicated (Supplementary Table S3). In addition, 3 different *P* value thresholds (*P* < 5 × 10^−7^, *P* < 5 × 10^−9^, and *P* < 5 × 10^−10^) for selecting *trans* SNPs were examined (Supplementary Table S3). All the association results were consistent with those in our main analysis. The above results showed the robustness of our main results.

Based on a comparison of exome-sequencing data of tumor tissue and tumor-adjacent normal tissue obtained from 150 TCGA PDAC patients, the somatic-level changes of potentially functional variants/mutations were observed in at least 1 patient for 10 of the 39 genes encoding identified associated proteins (Supplementary Table S4). This proportion (10/39 = 25.64%) is significantly higher (enrichment *P* < 0.00001) than the overall observed proportion of potentially functional changes across the genes encoding the proteins tested for association analyses (95/1,218 = 7.80%; here 1,218 represents the number of the genes available in TCGA analysis as part of the genes encoding the 1,389 assessed proteins).

**Figure 3:**
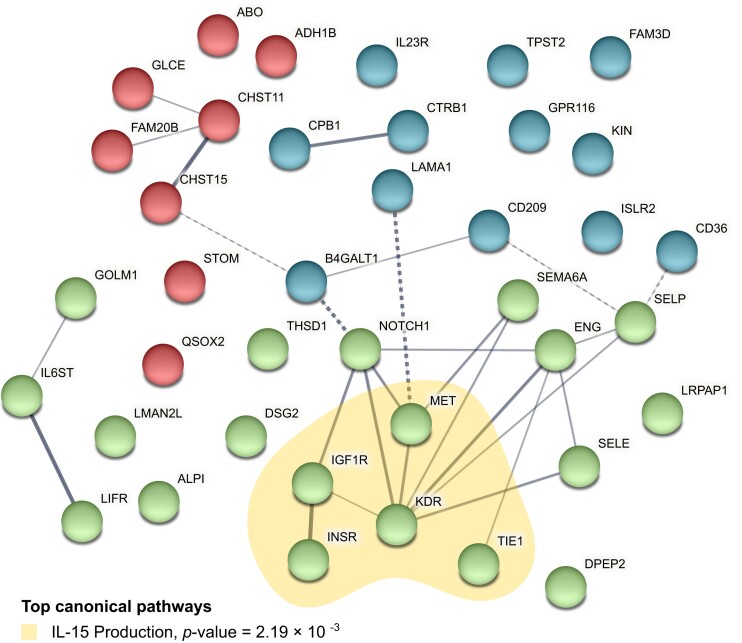
PPI network and canonical pathways of 40 identified proteins associated with PDAC risk. Network nodes represent proteins, edge thickness is proportional to the evidence for the PPI, and dashed lines represent the interaction among clusters. The enrichment of canonical pathways was determined using IPA software.

According to the IPA analysis, several cancer-related functions were enriched for the genes encoding our identified proteins (Supplementary Table S5). The top canonical pathways identified included IL-15 production (*P* = 2.21 × 10^−3^), Heparan Sulfate Biosynthesis (Late Stages) (*P* = 2.97 × 10^−3^), Heparan Sulfate Biosynthesis (*P* = 3.99 × 10^−3^), Sperm Motility (*P* = 7.73 × 10^−3^), and Dermatan Sulfate Biosynthesis (Late Stages) (*P* = 0.01) (Fig. 3). Among the related networks, the top network was cell-to-cell signaling and interaction, cardiovascular system development and function, and organismal development (Supplementary Fig. S1), followed by cancer, organismal injury and abnormalities, respiratory disease, free radical scavenging, cell death and survival, organismal injury and abnormalities, carbohydrate metabolism, small molecule biochemistry, cell cycle, and cancer, cell-to-cell signaling and interaction, and cellular assembly and organization. Interactions among identified proteins were investigated based on STRING database (Fig. 3). In the network, KDR was predicted to interact with IGF1R, NOTCH1, MET, SEMA6A, ENG, SELP, and SELE.

Based on interrogation using the OpenTargets and DrugBank database, 10 of the identified proteins are supported to be relevant to PDAC (overall score >0 in OpenTargets) and are targets of existing drugs approved to be used to treat human conditions (Table 3). Our work indicates potential drug repurposing opportunities of these drug targets to other indications. The scores of molecular docking between each of the proteins and the corresponding meta-drug agents are included in Table 3.

**Table 3:** Drug repurposing opportunities

Protein	Protein full name	Protein-encoding gene	OpenTargets information (overall score)	Drugbank ID	Drug name	Molecular action	Molecular docking score*
sTie-1	Tyrosine-protein kinase receptor Tie-1, soluble	*TIE1*	0.006	DB12010	Fostamatinib	Inhibitor	−6.1
Carboxypeptidase B1	Carboxypeptidase B	*CPB1*	0.159	DB04272	Citric acid	NA	−3.9
Chymotrypsin	Chymotrypsinogen B	*CTRB1*	0.078	DB06692	Aprotinin	NA	MDNA
sE-Selectin	E-selectin	*SELE*	0.023	DB01136	Carvedilol	Inhibitor	−6.9
P-Selectin	P-Selectin	*SELP*	0.008	DB01109	Heparin	Inhibitor	−4.9
				DB08813	Nadroparin	Inhibitor	−4.9
				DB06779	Dalteparin	Inhibitor	−4.9
				DB15271	Crizanlizumab	Inhibitor	3DSNA
VEGF sR2	Vascular endothelial growth factor receptor 2	*KDR*	0.367	DB06589	Pazopanib	Inhibitor	−6.3
				DB08896	Regorafenib	Inhibitor	−6.5
				DB09079	Nintedanib	Inhibitor	−5.8
				DB14840	Ripretinib	Inhibitor	−6.6
				DB00398	Sorafenib	Antagonist	−6.6
				DB01268	Sunitinib	Inhibitor	−5.6
				DB06595	Midostaurin	Antagonist inhibitor	−5.1
				DB06626	Axitinib	Inhibitor	−6.0
				DB08875	Cabozantinib	Antagonist	−**7.0**
				DB08901	Ponatinib	Inhibitor	−6.9
				DB09078	Lenvatinib	Inhibitor	−6.1
				DB05578	Ramucirumab	Antagonist	3DSNA
				DB12010	Fostamatinib	Inhibitor	−5.3
				DB12147	Erdafitinib	Substrate	−5.5
				DB15822	Pralsetinib	Inhibitor	−6.9
				DB11800	Tivozanib	Inhibitor	−6.4
ADH1B	Alcohol dehydrogenase 1B	*ADH1B*	0.001	DB00898	Ethanol	Substrate	−2.8
				DB09462	Glycerin	NA	−3.7
				DB00157	NADH	Substrate	−**9.6**
				DB01213	Fomepizole	Inhibitor	−3.9
Met	Hepatocyte growth factor receptor	*MET*	0.304	DB08865	Crizotinib	Inhibitor	−**8.1**
				DB08875	Cabozantinib	Antagonist	−**8**
				DB12267	Brigatinib	Inhibitor	−**8.2**
				DB12010	Fostamatinib	Inhibitor	−6.7
				DB11791	Capmatinib	Inhibitor	−**8.7**
				DB15133	Tepotinib	Inhibitor	−**8.3**
				DB11800	Tivozanib	Inhibitor	−**8.2**
				DB16695	Amivantamab	Antagonist antibody	3DSNA
IGF-I sR	Insulin-like growth factor 1 receptor	*IGF1R*	0.099	DB00071	Insulin pork	NA	MDNA
				DB00046	Insulin lispro	Activator	MDNA
				DB01307	Insulin detemir	Activator	MDNA
				DB00047	Insulin glargine	Activator	MDNA
				DB01306	Insulin aspart	Activator	MDNA
				DB01309	Insulin glulisine	Activator	MDNA
				DB09564	Insulin degludec	Activator	MDNA
				DB14751	Mecasermin rinfabate	Agonist	MDNA
				DB09456	Insulin beef	Activator	MDNA
				DB08804	Nandrolone decanoate	Inducer	−5.8
				DB01277	Mecasermin	Agonist	3DSNA
				DB00030	Insulin human	Activator	MDNA
				DB06343	Teprotumumab	Binder, antibody	3DSNA
				DB12267	Brigatinib	Inhibitor	−5.7
IR	Insulin receptor	*INSR*	0.013	DB00047	Insulin glargine	Agonist	MDNA
				DB00071	Insulin pork	Binder	MDNA
				DB01307	Insulin detemir	Agonist	MDNA
				DB00046	Insulin lispro	Agonist	MDNA
				DB01306	Insulin aspart	Agonist	MDNA
				DB01309	Insulin glulisine	Agonist	MDNA
				DB09564	Insulin degludec	Agonist	MDNA
				DB09129	Chromic chloride	Activator	MDNA
				DB14751	Mecasermin rinfabate	NA	MDNA
				DB09456	Insulin beef	Agonist	MDNA
				DB00030	Insulin human	Agonist	MDNA
				DB01277	Mecasermin	NA	3DSNA
				DB12267	Brigatinib	Binding	−**8.4**
				DB12010	Fostamatinib	Inhibitor	−**7.5**

* A score of ≤−7 represents a good interaction between the protein and corresponding drug agent and is bolded.

MDNA: molecular docking not applicable; 3DSNA: 3D structure not available.

**Figure 4:**
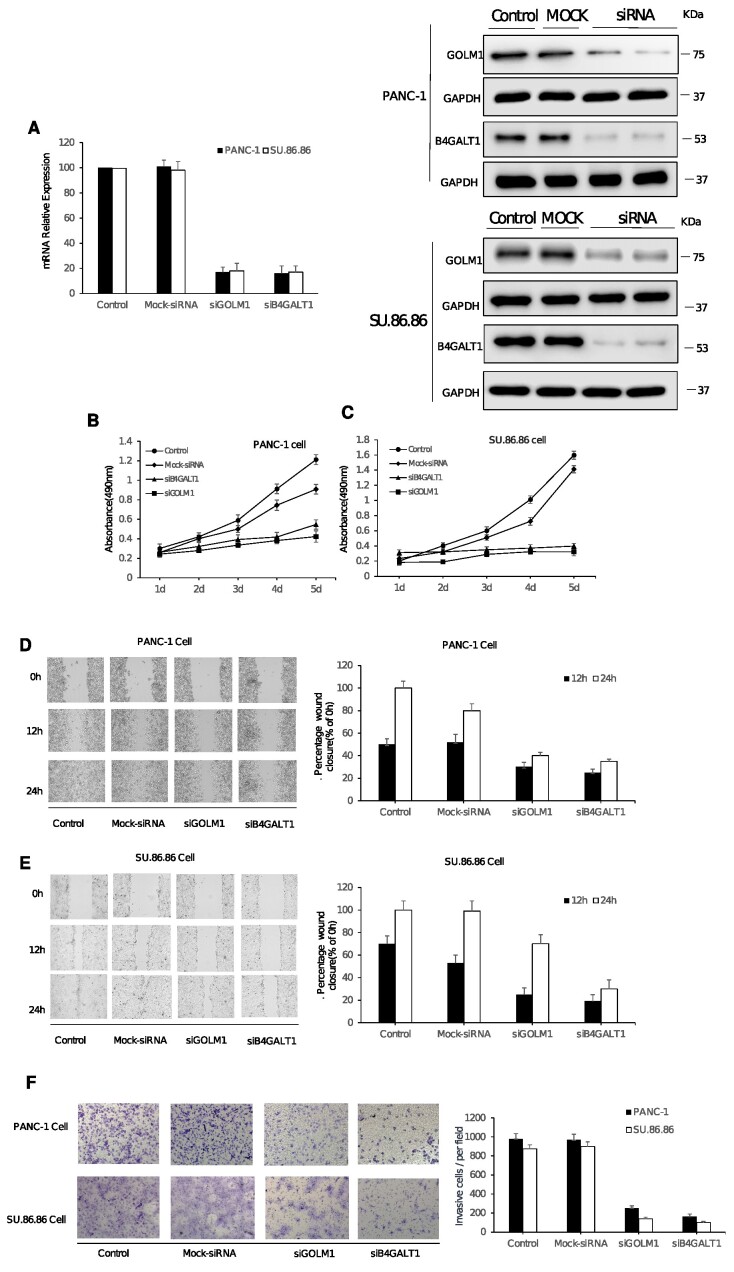
The analysis of cell proliferation, migration, and invasion on PANC-1 and SU.86.86 cells with siB4GALT1 and siGOLM1 transfection. The quantitative real-time PCR (qPCR) assay and the Western blot assay (A) were used to investigate the RNAi effect of siB4GALT1 and siGOLM1 (40 nM, 72h) in PANC-1 and SU.86.86 cells. GAPDH was used as an internal control for qPCR analyses and Western blot analyses, respectively. (B, C) The effect of transfection with siB4GALT1 and siGOLM1 (40 nM) on cell proliferation. The cells were detected by MTS [3-(4,5-dimethylthiazol-2-yl)-5-(3-carboxymethoxyphenyl)-2-(4-sulfophenyl)-2H-tetrazolium] assay on each day for 5 consecutive days. (D, E) Silencing of *B4GALT1* and *GOLM1* inhibited migration of PANC-1 and SU.86.86 cells. Representative images of wound scratch assay were performed to evaluate the motility of cells after silencing *B4GALT1* and *GOLM1*. After transfection, a scratch was made on the cell monolayer and was monitored with microscopy every 12 hours (0, 12, and 24h). Bar graphs show normalized wound area, calculated using Gen 5. Representative images of invasion assay. Data are represented as mean ± SD from triplicate samples, where *P < 0.01 compared to the control. (F) Effect of siB4GALT1 and siGOLM1 transfection on the invasion of PANC-1 and SU.86.86 cells. After siB4GALT1 and siGOLM1 transfection for 48h, the invasive ability of PANC-1 and SU.86.86 cells were identified by Transwell assay. **P < 0.01 compared with the control cells; ^##^*P* < 0.01 compared with the mock cells; data are expressed as the mean ± SD, *n* = 3.

Among the 16 novel associated proteins, analysis of TGCA data also revealed potential relevance of B4GT1 and GOLM1 with tumor development (Supplementary Figs. S2 and S3). The examination of *GOLM1* and *B4GALT1* gene expression in PAAD cancer was conducted using Gene Expression Profiling Interactive Analysis (GEPIA). The analysis involved a dataset consisting of 179 tumor samples and 171 normal controls. The boxplot analysis revealed a statistically significant increase in *GOLM1* (Supplementary Fig. S2A) and *B4GALT1* (Supplementary Fig. S3A) expression in the tumor samples as compared with the normal control group. GEPIA, accessible through [44], served as the platform for this investigation. The survival analysis of *GOLM1* and *B4GALT1* gene expression in PAAD cancer was conducted using GEPIA. Survival plots revealed a significant decrease in overall survival (OS) and disease-free survival (DFS) among tumor samples exhibiting elevated *GOLM1* or *B4GALT1* expression (*n* = 89) compared with those with low expression (*n* = 89). Employing the log-rank test for hypothesis testing, our findings emphasize a noteworthy correlation between heightened gene expression and reduced OS and DFS in the PAAD cancer cohort (Supplementary Fig. S2B, C and Supplementary Fig. 3B, C). Consequently, these 2 proteins were selected as the targets for experimental validation to further investigate their potential roles in PDAC development. Two gene-specific siRNAs (siGOLM1 and siB4GALT1) were employed for posttranscriptional gene silencing of *GOLM1* and *B4GALT1*, resulting in the knockdown of these 2 genes. As depicted in Fig. 4A, qPCR analysis demonstrated a significant reduction in the mRNA expression of *GOLM1* and *B4GALT1* in PANC-1 and SU.86.86 cells at 72 hours after transfection with siGOLM1 or siB4GALT1 (40 nM) when compared with the untreated control group (*P* < 0.05). No significant difference was observed between the negative control group (NC, mock-siRNA transfection) and the control groups (Fig. 4A). This trend was also consistent in the Western blot analysis (Fig. 4B) in comparison with the qPCR assay, indicating that siGOLM1 and siB4GALT1 effectively reduce the expression of *GOLM1* and *B4GALT1* at both mRNA and protein levels in PANC-1 and SU.86.86 cells.

To assess the biological impact of *GOLM1* and *B4GALT1* silencing in PANC-1 and SU.86.86 cells, cell proliferation was examined using the MTS assay over a span of 5 consecutive days. As shown in Fig. 4C and D, transfection of siGOLM1 and siB4GALT1 inhibited cell proliferation in both PANC-1 and SU.86.86 cells compared with the control (untransfected) and NC (mock-siRNA transfected) groups. Furthermore, a wound-healing assay demonstrated that at 12 and 24 hours postscratch treatment, the open wound area in *GOLM1* and *B4GALT1* siRNA-transfected cells was significantly larger than that in mock siRNA-transfected or untransfected cells (Fig. 4D, E), implying that knockdown of *GOLM1* and *B4GALT1* in PANC-1 and SU.86.86 cells effectively inhibited cell migration *in vitro*. To investigate whether the downregulation of *GOLM1* and *B4GALT1* affects the invasive capabilities of PANC-1 and SU.86.86 cells, a Transwell analysis was performed. The results revealed a significant inhibition of cell invasion in PANC-1 and SU.86.86 cells upon *GOLM1* or *B4GALT1* silencing. The number of siGOLM1- or siB4GALT1-transfected cells invading through the membrane was markedly lower than that of control-siRNA transfected cells (Fig.   4F, *P* < 0.05). Together, our findings suggest that GOLM1 and B4GT1 play crucial roles in PDAC cell proliferation, migration, and invasion, and their suppression could potentially serve as a therapeutic strategy for PDAC.

## Discussion

This is the first PWAS study using comprehensive protein genetic prediction models to assess the associations between genetically predicted circulating protein concentrations and PDAC risk. Overall, we identified 40 proteins that were significantly associated with PDAC risk after FDR correction, including 16 novel proteins that have not been previously reported. Our results suggest new knowledge on the genetics and etiology of PDAC, and the newly identified proteins could serve as candidate blood biomarkers for risk assessment of PDAC, a highly fatal malignancy. We also identified potential drug repurposing opportunities targeting the identified proteins, which warrant further investigations.

In previous studies, blood concentrations of specific proteins such as CA242, PIVKA-II, PAM4, S100A6, OPN, RBM6, EphA2, and OPG have been reported to be potentially associated with PDAC risk [4–7]. In the INTERVAL dataset, proteins S100A6 and OPG were captured, and we were able to develop satisfactory prediction models for their levels in blood [17]. We observed a significant association with the same direction for OPG (*P* = 0.03, *z* score = 2.23) but not for S100A6 (*P* = 0.93) with PDAC risk. Such inconsistent findings with previous studies might be explained by potential biases in previous epidemiological studies and warrant further exploration.

In this large study, we identified 16 novel proteins that were associated with PDAC risk. Previous studies have suggested potential roles for some of the novel proteins in pancreatic tumorigenesis. Tie1 deficiency is reported to induce endothelial–mesenchymal transition (EndMT) and promote a motile phenotype [46]. EndMT is known to present in human pancreatic tumors [46]. Another study reports that TNF-α, which is abundantly present in PDAC, induces EndMT and acts at least partially through TIE1 regulation in murine pancreatic tumors [47]. For CPB1, immunohistochemistry of tissue microarray from patients with PDAC showed that it was significantly downregulated in pancreatic tumor compared with adjacent normal pancreatic tissues [48]. This aligns with the negative association between genetically predicted levels of carboxypeptidase B1 and PDAC risk observed in this study. In another study, it was reported that mutations in *CPB1* were associated with pancreatic cancer [49]. Regarding GOLM1, 1 study supported that long noncoding RNA TP73-AS1 could promote pancreatic cancer progression through GOLM1 upregulation by competitively binding to miR-128–3p [50]. Further investigations are warranted to clarify roles of the identified proteins in pancreatic cancer development.

Based on drug repurposing analyses, we prioritized several drugs that may serve as promising candidates for treating PDAC, such as crizotinib, cabozantinib, brigatinib, capmatinib, tepotinib, and tivozanib targeting Hepatocyte growth factor receptor (Met). Previous research has supported potential link between these drugs and PDAC. For example, earlier research found that crizotinib and cabozantinib could decrease PDAC cell line viability *in vitro* [51]. Cabozantinib together with photodynamic therapy had been shown to achieve local control and decrease in tumor metastases in preclinical PDAC models [52]. A translational mathematical modeling study revealed that tepotinib at a dose selection of 500 mg once daily could be effective for PDAC [53]. Further work is needed to assess potential efficacy of these drug candidates in PDAC treatment.

There are several strengths of this study for detecting proteins associated with PDAC risk. We developed comprehensive protein genetic prediction models as instruments, which not only potentially minimize biases commonly encountered in conventional observational study design but also bring improved statistical power compared with the design of only using pQTLs as instruments. However, several limitations of this study need to be recognized when interpreting our findings. First, our results may still be susceptible to potential pleiotropic effects and may not necessarily infer causality. Similar to the design of the TWAS, our PWAS should be useful for prioritizing causal proteins; however, we cannot completely exclude the possibility of false-positive findings for some of the identified associations [54]. Several likely reasons may induce these, such as correlated protein expression across participants, correlated genetically predicted protein expression, and shared genetic variants [54]. Future functional investigation will better characterize whether the identified proteins play a causal role in PDAC development. Second, since in this work, the genetically regulated components of plasma protein levels were studied but not the overall measured levels, the utility of the identified proteins as risk biomarkers for PDAC remains unclear. Additional work for measuring circulating protein levels in prediagnostic blood samples is needed to evaluate the prediction role of these proteins in PDAC risk. Third, for our current model development design, the candidate predictors for each protein of interest merely rely on the potentially associated SNPs at a specific statistical threshold. A small proportion of proteins were excluded for downstream model construction because of the lack of such SNPs. Future work considering additional potential predictors beyond such statistics-based selection would be needed to improve the ability to evaluate additional proteins. Fourth, previous work has supported that covariates of smoking and body mass index are related to blood protein levels [55, 56]. In the current study using INTERVAL resources, we were not able to adjust for these covariates during model construction. Further study is thus needed to validate our results. Lastly, the current study largely focuses on Europeans for both protein genetic prediction model development and downstream association analyses with PDAC risk. Future research is warranted to study proteins associated with PDAC risk in other non-European ancestries.

Our TGCA data analysis has revealed potential relevance of B4GT1 and GOLM1 in tumorigenesis and tumor progression. B4GT1 (beta-1,4-galactosyl transferase 1) is an enzyme primarily responsible for catalyzing the galactose transfer to specific receptor molecules within organisms [57]. Its significance lies in its involvement in various essential biological processes, such as intercellular communication and cell adhesion. Furthermore, alterations in the expression level of B4GT1 have been observed in certain cancers, suggesting its potential implication in tumor initiation and development [58]. This intriguing finding has led us to select B4GT1 as a priority target for further exploration of its role in PDAC using experimental techniques. Similarly, our attention was drawn to GOLM1 (Golgi membrane protein 1), a membrane protein predominantly located in the Golgi apparatus, which plays a pivotal role in cellular secretion and transport processes. Recent investigations have demonstrated an upregulation of GOLM1 expression in multiple cancer types, including liver cancer, lung cancer, and pancreatic cancer. Such evidence strongly suggests that GOLM1 might exert a significant influence on the onset and progression of these malignancies [59]. Consequently, we selected GOLM1 as an additional focus for verification to gain deeper insights into its involvement in PDAC. By utilizing RNA interference (RNAi) technology to silence these genes, our experimental results corroborated the critical roles of GOLM1 and B4GT1 in driving PDAC cell proliferation, migration, and invasion. Subduing these genes holds promise as a potential therapeutic approach for PDAC treatment.

In summary, using protein genetic prediction models, we identified 16 novel protein biomarker candidates for which the genetically predicted circulating levels were significantly associated with PDAC risk. Future work is needed to better characterize the potential roles of these proteins in the etiology of PDAC development, assess the predictive role of such markers in risk assessment of PDAC, and evaluate whether the potential drug repurposing opportunities we identified may improve PDAC outcomes.

## Supplementary Material

giae012_GIGA-D-23-00321_Original_Submission

giae012_GIGA-D-23-00321_Revision_1

giae012_Response_to_Reviewer_Comments_Original_Submission

giae012_Reviewer_1_Report_Original_Submission Jian Sang -- 12/18/2023 Reviewed

giae012_Reviewer_1_Report_Revision_1 Jian Sang -- 1/30/2024 Reviewed

giae012_Reviewer_2_Report_Original_Submission Huang Peng -- 1/2/2024 Reviewed

giae012_Reviewer_2_Report_Revision_1 Huang Peng -- 1/22/2024 Reviewed

giae012_Supplemental_File

## Data Availability

The pancreatic cancer genetic datasets used for the association analyses described in this article can be obtained from dbGaP [60] (accession numbers phs000206.v5.p3 and phs000648.v1.p1). The INTERVAL individual-level genotype and protein data, as well as full summary association results from the genetic analysis, are available through the European Genotype Archive (accession number EGAS00001002555). Summary association results are also publicly available at the NHGRI-EBI GWAS Catalog (https://www.ebi.ac.uk/gwas/downloads/summary-statistics) [61]. Other data further supporting this work are openly available in the *GigaScience* repository, GigaDB [62].

## Additional Files

**Supplementary Fig. S1**. The top networks identified by IPA. (A) Network 1 and (B) network 2. The nodes marked with red indicate proteins associated with pancreatic cancer risk. A solid line represents a direct interaction between 2 nodes, and a dotted line indicates an indirect interaction.

**Supplementary Fig. S2**. (A) Boxplot analysis of *GOLM1* gene expression in PAAD cancer using GEPIA. The plot compares expression levels between tumor (*n* = 179) and normal control (*n* = 171) samples. The analysis was conducted based on RNA sequencing data from TCGA and GTEx projects (**P* < 0.01). (B, C) Survival analysis of *GOLM1* gene expression in PAAD cancer using GEPIA. The survival plot compares the overall survival (OS) (B) and disease-free survival (DFS) (C) between tumor samples with high *GOLM1* expression (*n* = 89) and low *GOLM1* expression (*n* = 89). The analysis utilized the log-rank test for hypothesis testing, with a significant finding indicating a shorter OS and DFS in the high *GOLM1* expression group compared with the low *GOLM1* expression group. Cohort thresholds and expression cutoffs were set based on user-defined parameters in the GEPIA platform.

**Supplementary Fig. S3**. (A) Boxplot analysis of *B4GALT1* gene expression in PAAD cancer using GEPIA. The plot compares expression levels between tumor (*n* = 179) and normal control (*n* = 171) samples. The analysis was conducted based on RNA sequencing data from TCGA and GTEx projects (**P* < 0.01). (B, C) Survival analysis of *B4GALT1* gene expression in PAAD cancer using GEPIA. The survival plot compares the overall survival (OS) (A) and the disease-free survival (DFS) (B) between tumor samples with high *B4GALT1* expression (*n* = 89) and low *B4GALT1* expression (*n* = 89). The analysis utilized the log-rank test for hypothesis testing, with a significant finding indicating a shorter OS and DFS in the high *B4GALT1* expression group compared to the low *B4GALT1* expression group. Cohort thresholds and expression cutoffs were set based on user-defined parameters in the GEPIA platform.

**Supplementary Table S1**. Associations of proteins identified using pQTL as instruments but not show a significant association with pancreatic cancer risk in the current study.

**Supplementary Table S2**. Comparison of heritability between *cis* + *trans* models and *cis*-only models.

**Supplementary Table S3**. Robustness analysis.

**Supplementary Table S4**. Somatic-level potentially deleterious changes of genes encoding identified proteins in TCGA pancreatic adenocarcinoma patients.

**Supplementary Table S5**. Top diseases, biofunctions, and networks associated with the genes encoding identified pancreatic cancer risk-associated proteins.

## Abbreviations

FDR: false discovery rate; GWAS: genome-wide association studies; HWE: Hardy–Weinberg equilibrium; PAAD: pancreatic adenocarcinoma; PanC4: Pancreatic Cancer Case-Control Consortium; PanScan: Pancreatic Cancer Cohort Consortium; PDAC: pancreatic ductal adenocarcinoma; pQTL: protein quantitative trait loci; QC: quality control.
