# Supplementary material for: Proteome-wide association study and functional validation identify novel protein markers for pancreatic ductal adenocarcinoma
Source: Gigascience. 2024 Apr 12;13:giae012. doi: 10.1093/gigascience/giae012 (PMC11010651; doi:10.1093/gigascience/giae012)
Supplement: giae012_GIGA-D-23-00321_Original_Submission [file giae012_giga-d-23-00321_original_submission.pdf]

GigaScience

Proteome-wide association study and functional validation identify novel protein markers for pancreatic ductal adenocarcinoma

--Manuscript Draft--

**Manuscript Number:**	GIGA-D-23-00321
**Full Title:**	Proteome-wide association study and functional validation identify novel protein markers for pancreatic ductal adenocarcinoma
**Article Type:**	Research
**Funding Information:**	

Funder	Recipient
NIH/NCI (9 SC1 GM135050-05)	Dr. Yong Wu
University of Hawaii Cancer Center and V Foundation V Scholar Award	Not applicable
National Cancer Institute (R01CA263494)	Dr. Chong Wu<br>Associated Professor Lang Wu
National Human Genome Research Institute (U54HG013243)	Associated Professor Lang Wu
National Cancer Institute (R00CA218892)	Associated Professor Lang Wu
National Institute on Minority Health and Health Disparities (U54MD007598)	Not applicable
NIH/NCI (1U54CA14393)	Not applicable
NIH/NCI (U56 CA101599-01)	Not applicable
Department-of-Defense Breast Cancer Research Program (BC043180)	Dr. Jaydutt V. Vadgama
NIH/NCATS (CTSI UL1TR000124)	Dr. Jaydutt V. Vadgama
Accelerating Excellence in Translational Science Pilot Grants (G0812D05)	Dr. Yong Wu
NIH/NCI (SC1CA200517)	Dr. Yong Wu
VA Merit Award (1 I01 CX001822-01A2)	Dr. Qizhi Yao
National Cancer Institute (NCI), US National Institutes of Health (NIH) (HHSN261200800001E)	Not applicable
NIH/NCI (K07 CA140790)	Not applicable
the American Society of Clinical Oncology Conquer Cancer Foundation	Not applicable
the Howard Hughes Medical Institute	Not applicable
the Lustgarten Foundation	Not applicable
he Robert T. and Judith B. Hale Fund for Pancreatic Cancer Research	Not applicable
Promises for Purple	Not applicable
NCI (R01CA154823)	Not applicable
National Institutes of Health to The Johns Hopkins University (HHSN2682011000111)	Not applicable
National Institute for Health Research (NIHR)	Not applicable
NIHR BioResource	Not applicable

Powered by Editorial Manager® and ProduXion Manager® from Aries Systems Corporation

	NIHR Cambridge Biomedical Research Centre (BRC-1215-20014)	Not applicable
	NIHR Blood and Transplant Research Unit in Donor Health and Genomics (NIHR BTRU-2014-10024)	Not applicable
	UK Medical Research Council (MR/L003120/1)	Not applicable
	British Heart Foundation (SP/09/002; RG/13/13/30194; RG/18/13/33946)	Not applicable
	NIHR Cambridge BRC (BRC-1215-20014)	Not applicable

Abstract:

Abstract
Pancreatic ductal adenocarcinoma (PDAC) remains a lethal malignancy, largely due to the paucity of reliable biomarkers for early detection and therapeutic targeting. Existing blood protein biomarkers for PDAC often suffer from replicability issues, arising from inherent limitations such as unmeasured confounding factors in conventional epidemiologic study designs. To circumvent these limitations, we use genetic instruments to identify proteins with genetically predicted levels to be associated with PDAC risk. Leveraging genome and plasma proteome data from the INTERVAL study, we established and validated models to predict protein levels using genetic variants. By examining 8,275 PDAC cases and 6,723 controls, we identified 40 associated proteins, of which 16 are novel. Functionally validating these candidates by focusing on two selected novel protein-encoding genes, GOLMA1 and B4GALT1, we demonstrated their pivotal roles in driving PDAC cell proliferation, migration, and invasion. Furthermore, we also identified potential drug repurposing opportunities for treating PDAC.

Significance:
PDAC is a notoriously difficult-to-treat malignancy, and our limited understanding of causal protein markers hampers progress in developing effective early detection strategies and treatments. Our study identifies novel causal proteins using genetic instruments and subsequently functionally validates selected novel proteins. This dual approach enhances our understanding of PDAC etiology and potentially opens new avenues for therapeutic interventions.

Keywords: Biomarkers, protein, genetics, pancreatic cancer, risk

**Corresponding Author:**	Lang Wu<br>University of Hawai'i at Manoa<br>Honolulu, UNITED STATES
**Corresponding Author Secondary Information:**	
**Corresponding Author's Institution:**	University of Hawai'i at Manoa
**Corresponding Author's Secondary Institution:**	
**First Author:**	Jingjing Zhu
**First Author Secondary Information:**	
**Order of Authors:**	Jingjing Zhu
	Ke Wu
	Shuai Liu
	Alexandra Masca
	Hua Zhong
	Tai Yang
	Dalia H Ghoneim
	Praveen Surendran
	Tanxin Liu

Powered by Editorial Manager® and ProduXion Manager® from Aries Systems Corporation

	Qizhi Yao
	Tao Liu
	Sarah Fahle
	Adam Butterworth
	Md Ashad Alam
	Jaydutt V. Vadgama
	Youping Deng
	Hong-Wen Deng
	Chong Wu
	Yong Wu
	Lang Wu
**Order of Authors Secondary Information:**	
**Additional Information:**	
**Question**	**Response**
Are you submitting this manuscript to a special series or article collection?	No
**Experimental design and statistics**<br><br>Full details of the experimental design and statistical methods used should be given in the Methods section, as detailed in our Minimum Standards Reporting Checklist. Information essential to interpreting the data presented should be made available in the figure legends.<br><br>Have you included all the information requested in your manuscript?	Yes
**Resources**<br><br>A description of all resources used, including antibodies, cell lines, animals and software tools, with enough information to allow them to be uniquely identified, should be included in the Methods section. Authors are strongly encouraged to cite Research Resource Identifiers (RRIDs) for antibodies, model organisms and tools, where possible.<br><br>Have you included the information requested as detailed in our Minimum	Yes

Standards Reporting Checklist?	
**Availability of data and materials**<br><br>All datasets and code on which the conclusions of the paper rely must be either included in your submission or deposited in publicly available repositories (where available and ethically appropriate), referencing such data using a unique identifier in the references and in the “Availability of Data and Materials” section of your manuscript.<br><br>Have you have met the above requirement as detailed in our Minimum Standards Reporting Checklist?	Yes

Manuscript Click here to access/download;Manuscript;Manuscript.docx

Proteome-wide association study and functional validation identify novel protein markers for pancreatic ductal adenocarcinoma

Jingjing Zhu[1*], Ke Wu[2*], Shuai Liu[1*], Alexandra Masca[1], Hua Zhong[1], Tai Yang[3], Dalia H Ghoneim[1], Praveen Surendran[4], Tanxin Liu[5], Qizhi Yao[6,7], Tao Liu[8], Sarah Fahle[4], Adam Butterworth[4,9], Md Ashad Alam[10], Jaydutt V. Vadgama[2], Youping Deng[11], Hong-Wen Deng[10], Chong Wu[12#], Yong Wu[2#], Lang Wu[1#]

1. Cancer Epidemiology Division, Population Sciences in the Pacific Program, University of Hawaii Cancer Center, University of Hawaii at Manoa, Honolulu, HI, USA

2. Division of Cancer Research and Training, Department of Internal Medicine, Charles R. Drew University of Medicine and Science, David Geffen UCLA School of Medicine and UCLA Jonsson Comprehensive Cancer Center, Los Angeles, CA 90095, USA

3. Department of Biostatistics, University of Michigan - Ann Arbor, MI, USA

4. MRC/BHF Cardiovascular Epidemiology Unit, Department of Public Health and Primary Care, University of Cambridge, Cambridge, UK

5. Department of Epidemiology, Johns Hopkins Bloomberg School of Public Health, Baltimore, MD, USA

6. Division of Surgical Oncology, Michael E. DeBakey Department of Surgery, Baylor College of Medicine, Houston, Texas

7. Center for Translational Research on Inflammatory Diseases (CTRID), Michael E. DeBakey VA Medical Center, Houston, Texas

8. Biological Sciences Division, Pacific Northwest National Laboratory, Richland, WA 99354, USA

9. NIHR Blood and Transplant Research Unit in Donor Health and Genomics, Department of Public Health and Primary Care, University of Cambridge, Cambridge, UK

10. Tulane Center for Biomedical Informatics and Genomics, Division of Biomedical Informatics and Genomics, Deming Department of Medicine, Tulane University, 1440 Canal Street, New Orleans, 70112, LA, USA

11. Department of Quantitative Health Sciences, John A. Burns School of Medicine, University of Hawaii at Manoa, Honolulu, HI, USA.

12. Department of Biostatistics, The University of Texas MD Anderson Cancer Center, Houston, TX, USA

[*] these authors contributed equally to this work and are co-first authors

# these authors jointly supervised this work and are co-senior authors

Running title: Predicted protein biomarkers for pancreatic cancer

Abbreviations list:

Pancreatic ductal adenocarcinoma (PDAC)

protein quantitative trait loci (pQTL)

Genome-wide association studies (GWAS)

the Pancreatic Cancer Cohort Consortium (PanScan)

the Pancreatic Cancer Case-Control Consortium (PanC4)

quality control (QC)

Hardy-Weinberg equilibrium (HWE)

false discovery rate (FDR)

Corresponding to: Lang Wu, Cancer Epidemiology Division, Population Sciences in the Pacific Program, University of Hawaii Cancer Center, University of Hawaii at Manoa, Honolulu, HI, 96813, USA. Email: lwu@cc.hawaii.edu. Phone: (808)564-5965; or Yong Wu, Department of Internal Medicine, Charles Drew University of Medicine and Science, Los Angeles, CA 90059, USA. Email: yongwu@cdrewu.edu; or Chong Wu, Department of Biostatistics, The University of Texas MD Anderson Cancer Center, Houston, TX, USA. Email: cwu18@mdanderson.org

Competing financial interests

L.W. provided consulting service to Pupil Bio Inc. and received honorarium. No potential conflicts of interest were disclosed by the other authors.

Author contributions

L.W. conceived the study. Y.W. designed the functional experiments and supervised the *in vitro* functional work. C.W. and J.Z. contributed to the study design and/or prediction model building. S.L. performed model building and statistical analyses. D.H.G. contributed to statistical analyses.

K.W. conducted *in vitro* functional work. J.Z. performed the drug repurposing curation. M.A.A. performed molecular docking analysis. H.Z. and S. L. contributed to the bioinformatics and pathway analyses. L.W., J.Z., K.W., Y.W., A.M., H.Z., and T.Y. wrote the first version of manuscript. D.H.G., P.S., T.L., E.P., Q.Y., T.L., S.F., J.V.V., H-W. D., Y.D., H.Z., S.L., and A.B. contributed to manuscript revision and/or INTERVAL data management. All authors have reviewed and approved the final manuscript.

Abstract

Pancreatic ductal adenocarcinoma (PDAC) remains a lethal malignancy, largely due to the paucity of reliable biomarkers for early detection and therapeutic targeting. Existing blood protein biomarkers for PDAC often suffer from replicability issues, arising from inherent limitations such as unmeasured confounding factors in conventional epidemiologic study designs. To circumvent these limitations, we use genetic instruments to identify proteins with genetically predicted levels to be associated with PDAC risk. Leveraging genome and plasma proteome data from the INTERVAL study, we established and validated models to predict protein levels using genetic variants. By examining 8,275 PDAC cases and 6,723 controls, we identified 40 associated proteins, of which 16 are novel. Functionally validating these candidates by focusing on two selected novel protein-encoding genes, *GOLM1* and *B4GALT1*, we demonstrated their pivotal roles in driving PDAC cell proliferation, migration, and invasion. Furthermore, we also identified potential drug repurposing opportunities for treating PDAC.

Significance:

PDAC is a notoriously difficult-to-treat malignancy, and our limited understanding of causal protein markers hampers progress in developing effective early detection strategies and treatments. Our study identifies novel causal proteins using genetic instruments and subsequently functionally validates selected novel proteins. This dual approach enhances our understanding of PDAC etiology and potentially opens new avenues for therapeutic interventions.

Keywords: Biomarkers, protein, genetics, pancreatic cancer, risk

Introduction

Pancreatic cancer is the seventh leading cause of cancer deaths in industrialized countries with pancreatic ductal adenocarcinoma (PDAC) making up over 90% of pancreatic cancer cases (1). According to GLOBOCAN 2020 cancer statistics, pancreatic cancer is the 14th most common cancer type with 495,773 new cases in 2020. There are almost the same number of deaths caused by pancreatic cancer (466,003 deaths) in 2020, accounting for 4.7% of all cancer related deaths (2). Owing to its often asymptomatic or non-specific symptoms during early stages, a majority of patients are usually diagnosed in advanced stages. This results in 80-90% of pancreatic tumors being unresectable upon diagnosis, leading to a dismal prognosis: a mere 9% five-year survival rate after diagnosis (1). Given these dire statistics, there is an urgent need to identify effective biomarkers for screening or early detection in high-risk populations. Equally crucial is the development of improved therapeutic strategies to improve PDAC outcome.

Currently, serum cancer antigen (CA) 19-9 is the only diagnostic biomarker for pancreatic cancer approved by the U.S. FDA. However, elevated levels of CA 19-9 are related to other conditions, and its performance as a diagnostic tool for pancreatic cancer is far from ideal (3): it has a poor positive predictive value (0.5-0.9%), along with restricted specificity (82-90%) and sensitivity (79-81%). Previous studies have also reported several other circulating blood protein biomarkers that are potentially associated with pancreatic cancer risk, such as CA242, PIVKA-II, and PAM4 (4-7). However, results from existing studies often involving small sample sizes and findings are inconsistent. It is well known that the conventional epidemiologic study design measuring levels of proteins directly may be subject to selection bias and residual or

unmeasured confounding, which could also contribute to the inconsistent findings in the existing literature.

An alternative design of using genetic instruments may decrease many limitations of existing studies, due to the nature of random assortment of alleles from parents to offspring during gamete formation (8,9). Inspired by transcriptome-wide association study (TWAS), one may build comprehensive genetic prediction models for each protein to capture the prediction value of multiple single nucleotide polymorphisms (SNPs). Unlike conventional TWAS type of methods, which typically focus solely on cis-acting variants, our study enhanced statistical power by integrating both cis- and trans-acting elements into our genetic prediction models. Furthermore, as TWAS or PWAS results imply causality under stringent valid instrumental variable assumptions, we further functionally validated two novel proteins.

In the current study, we applied such a study design to identify novel proteins associated with PDAC risk. To our knowledge, this is the first large-scale proteome wide association study (PWAS) using comprehensive protein genetic prediction models as instruments to assess the associations between genetically predicted blood concentrations of proteins and PDAC risk. We used data for 8,275 cases and 6,723 controls of European descent from the Pancreatic Cancer Cohort Consortium (PanScan) and the Pancreatic Cancer Case-Control Consortium (PanC4). Beyond identifying novel proteins, we functionally validated two of them. Moreover, we generated a list of drugs targeting the identified proteins which may serve as candidates for drug repurposing of PDAC.

Methods

Protein genetic prediction model development and validation

We leveraged the genome and plasma proteome data of healthy European subjects included in the INTERVAL study to establish (subcohort1) and validate (subcohort2) protein genetic prediction models. The details of the INTERVAL study data have been published previously (10-14). Briefly, participants were generally healthy. The SOMAscan assay was used to collect the relative levels of 3,620 plasma proteins or complexes. Quality control (QC) was performed at both the sample and SOMAmer level. Approximately ~830,000 genetic variants were measured on the Affymetrix Axiom UK Biobank genotyping array. Standard sample and variant QC were conducted. SNPs were phased using SHAPEIT3 and imputed using a combined 1000 Genomes Phase 3-UK10K reference panel, which resulted in over 87 million imputed variants. The SNPs were further filtered using criteria of 1) imputation quality of at least 0.7, 2) minor allele count of at least 5%, 3) Hardy Weinberg Equilibrium (HWE) $p \geq 5\times10^{-6}$, (4) missing rates < 5%, and (5) presenting in the 1000 Genome Project data for European populations. Overall there were 4,662,360 variants passing these criteria.

In subcohort 1 (N=2,481), as described elsewhere (10), protein concentrations were log transformed and adjusted for age, sex, duration between blood draw and processing, and the top three principal components. For the rank-inverse normalized residuals of each protein, we followed the TWAS/FUSION framework to establish prediction models, using nearby variants (within 100kb) of potentially associated SNPs as candidate predictors (15). A false discovery rate (FDR) < 0.05 was used to determine potentially associated SNPs in cis regions (within 1 Mb of the transcriptional start site (TSS) of the gene encoding the target protein of interest) and *P*-value $\leq 5\times10^{-8}$ was used to determine potentially associated SNPs in trans regions. We only included strand unambiguous SNPs. Four methods of best linear unbiased predictor (blup), elastic net, LASSO, and top1 were used to develop the models. For each protein of interest, the model

showing the most significant cross-validation *P*-value among those developed using the four methods was selected. For protein prediction models with $R^2 \geq 0.01$, external validation was conducted using genetic and protein data of subcohort 2 (N=820). Briefly, predicted protein expression levels were estimated by applying the developed protein prediction models to the genetic data, which were further compared with the measured levels for each protein of interest. Proteins with a model prediction R^2 of ≥ 0.01 in subcohort1 and a correlation coefficient of ≥ 0.1 in subcohort2 were selected for association analysis with PDAC risk.

Examine associations of genetically predicted protein levels with PDAC risk

To investigate the associations between genetically predicted circulating protein levels and PDAC risk, the validated protein genetic prediction models were applied to the summary statistics from a large GWAS of PDAC risk. In the present work, we used data from GWAS conducted in the PanScan and PanC4 consortia downloaded from the database of Genotypes and Phenotypes (dbGaP), including 8,275 PDAC cases and 6,723 controls of European ancestry. Detailed information on this dataset has been included elsewhere (16-18). Briefly, four GWAS studies, namely, PanScan I, PanScan II, PanScan III, and PanC4, were genotyped using the Illumina HumanHap550, 610-Quad, OmniExpress, and OmniExpressExome arrays, respectively. Standard QC procedures were performed according to the consortia guidelines (17). Study participants who were related to each other, had sex discordance, had genetic ancestry other than Europeans, had a low call rate (less than 98% and 94% in PanC4 and PanScan, respectively), or had missing information on age or sex were excluded. Duplicated SNPs, and those with a high missing call rate (at least 2% and 6% in PanC4 and PanScan, respectively) or with violations of Hardy-Weinberg equilibrium (HWE) ($P < 1\times10^{-4}$ and $P < 1\times10^{-7}$ in PanC4 and PanScan,

respectively), were also removed. Regarding SNP data from PanC4, those with minor allele frequency < 0.005, with more than two discordant calls in duplicate samples, with more than one Mendelian error in HapMap control trios, and those with sex difference in allele frequency > 0.2 or in heterozygosity > 0.3 for autosomes/XY in European descendants were further removed. We performed genotype imputation using Minimac3 after prephasing with SHAPEIT from a reference panel of the Haplotype Reference Consortium (r1.1 2016) (19,20). We retained imputed SNPs with an imputation quality of ≥0.3. The associations between individual genetic variants and PDAC risk were further estimated adjusting for age, sex and top principal components. The TWAS/FUSION framework was used to assess the protein-PDAC risk associations, by leveraging correlations between variants included in the prediction models based on the phase 3, 1000 Genomes Project data for European populations (15). We used the false discovery rate (FDR) corrected *P*-value threshold of ≤ 0.05 to determine significant associations between genetically predicted protein concentrations and risk of PDAC.

Somatic variants of genes encoding associated proteins

For each of the genes encoding the proteins that are identified to be associated with PDAC risk, we evaluated potentially deleterious somatic level mutations in 150 PDAC patients included in The Cancer Genome Atlas (TCGA). The potentially deleterious somatic variants include missense mutations, splice site mutations, nonstop mutations, nonsense mutations, frameshift mutations, in-frame mutations and translation start site mutations.

The somatic level genetic changes were called using MuTect2 (doi: https://doi.org/10.1101/861054) and deposited to the TCGA data portal. The enrichment of proportion of assessed genes containing such somatic level genetic events compared with the

proportion of all protein-coding genes across the genome was evaluated using socscistatistics online website (https://www.socscistatistics.com/tests/ztest/default2.aspx).

Ingenuity Pathway Analysis (IPA) and Protein-Protein Interaction (PPI) analysis

To further assess whether genes encoding the identified PDAC associated proteins are enriched in specific pathways, molecular and cellular functions, and networks, we performed the enrichment analysis using Ingenuity Pathway Analysis (IPA) software (21). The "enrichment" score (Fisher exact test *P* value) that measures overlap of observed and predicted regulated gene sets was generated for each of the tested gene sets. The most significant pathways and functions with an enrichment *P* value less than 0.05 were reported. We also built protein-protein interaction (PPI) network using STRING database version 11.5 (https://string-db.org/) with 0.400 confidence level (22). The STRING database integrates different curated databases containing information on known and predicted functional protein–protein associations.

Drug repurposing analysis

For the identified proteins, we further assessed whether there is any evidence supporting their potential roles in PDAC by using the OpenTargets (23). Focusing on those showing a potential relevance, we further mined evidence of their targeting drugs using the DrugBank (24) database. We also conducted molecular docking analysis for the identified proteins and corresponding candidate drug agents (25). Specifically, we downloaded the 3D structure of targeted proteins from Protein Data Bank (PDB) (26) with source code 1CPB, 3CDZ, 1IGR, 3DFK, 5NO06, and drug agents from the PubChem database (27). We further worked out molecular docking between each of the proteins and the corresponding meta-drug agents to calculate the binding affinity scores (kcal/mol) for each pair of proteins and drugs.

In vitro functional validation of genes encoding selected associated novel proteins

Cell Lines and Culture Condition

Human pancreatic cancer cell lines PANC-1 and SU.86.86 were obtained from ATCC (American Type Culture Collection). All cells were cultured in vitro in DMEM (Dulbecco's modified eagle medium) high glucose medium (Gibco, Novato, CA, United States) supplemented with 10% (v/v) fetal bovine serum (FBS) (Gibco). Cells were incubated at 37°C with 5% CO2.

Western blotting

Post 72-hour silencing, we processed control, B4GALT1-silenced, and GOLM1-silenced cells for Western blotting. Cells were lysed using RIPA buffer, and equal protein amounts were separated on 10% or 12% SDS polyacrylamide gels, then transferred onto PVDF membranes. To prevent non-specific antibody binding, membranes were blocked with 5% milk in TBS with 0.1% Tween for an hour. They were then probed with anti-B4GALT1, anti-GOLM1, and anti-GAPDH antibodies, followed by their respective HRP-conjugated secondary antibodies. Signal detection was performed using Pierce™ ECL Western Blotting Substrate and images were captured and analyzed using Odyssey FC and ImageStudio Software.

Quantitative Real-Time PCR (qPCR)

Total RNA was extracted from cells using TRNzol reagent according to the manufacturer's protocol. The concentration of RNA was determined using a UV spectrophotometer. Subsequently, 2 mg of total RNA was reverse transcribed into cDNA using the iScript™ cDNA Synthesis Kit. qPCR analysis was performed on the CFX96™ Real-Time PCR Detection System

using the iTaq™ Universal SYBR® Green Supermix. The aim was to detect the expression levels of three genes: B4GALT1, GOLM1, and GAPDH mRNAs. Specific primer pairs were used for each gene. For B4GALT1, the forward sequence was GTATTTTGGAGGTGTCTCTGCTC and the reverse sequence was GGGCGAGATATAGACATGCCTC. For GOLM1, the forward sequence was ATCACCACAGGTGAGAGGCTCA and the reverse sequence was ACTTCCTCTCCAGGTTGGTCTG. For the housekeeping gene GAPDH, the forward sequence was GTCTCCTCTGACTTCAACAGCG and the reverse sequence was ACCACCCTGTTGCTGTAGCCAA. During the qPCR analysis, melting curves were generated to detect primer-dimer formation and confirm the specificity of the gene-specific peaks for each target. To ensure accurate quantification, the expression data were normalized to the amount of GAPDH mRNA expressed.

Transfection of siRNA

The transfection of small-interfering RNA (siRNA) was performed using specific human siRNAs targeting GOLM1 (SASI_Hs01_00223155), B4GALT1 (SASI_Hs01_00080445), and the MISSION siRNA universal negative control, all of which were obtained from Sigma-Aldrich (St. Louis, MO). Cells were seeded in 6-well plates at a density of 1.5x105 cells per well and subsequently transfected with the siRNAs at a concentration of 40 nM. The transfection procedure utilized the lipofectamine 2000 reagent (Invitrogen, Carlsbad, CA, United States) following the manufacturer's recommended guidelines. Gene silencing at both mRNA and protein levels was typically observed 72 h post-transfection. As such, the cells were collected and subjected to assays at the 72-hour time point to assess the efficacy of gene silencing.

Cell Proliferation Assay

To observe cell proliferation, cells were transfected with Mock siRNA, siGOLM11 and siB4GAL1 (40 nM). At 24 h after transfection, the cells were trypsinized and seeded into 96-well plates (Corning, NY, United States) at a density of 5000 cells/well in 200 ul media. The plates were incubated in a 37°C humidified incubator. On each day for [3-(4,5-dimethylthiazol-2-yl)-5-(3-carboxymethoxyphenyl)-2-(4-sulfophenyl)-2H-tetrazolium] (MTS) assay.

***In vitro* invasion assay**

Cell invasion was assessed following transfection with Mock siRNA, siGOLM11, and siB4GAL1 (40 nM). A modified Boyden chamber method was employed. Matrigel (BD Biosciences) was coated on the upper chamber of Transwell inserts (Corning, 8 μm pore size) at a concentration of 300 μg/ml, allowing gel formation for 2 hours at 37°C. Cells (5 x 10^4) were then suspended in 200 μl of serum-free medium and added to the upper chamber. The lower chamber contained 600 μl of medium with 10% FBS, acting as a chemoattractant. Following 24 hours of incubation at 37°C, non-invading cells on the upper membrane surface were gently removed using a cotton swab. Cells that invaded the lower membrane surface were fixed with 4% paraformaldehyde and stained with 0.1% crystal violet. Invasion was quantified by counting the stained cells on the underside of the membrane using a light microscope (10 random fields at 200x magnification). All experiments were performed in triplicate to ensure robustness of the findings.

Wound Scratch assay

After 24 hours of transfection with Mock siRNA, siGOLM11, and siB4GAL1, PANC-1 and SU.86.86 cells were cultured in a 96-well plate to form a monolayer. Using BioTek's

AutoScratchTM Wound Making Tool, straight scratches were carefully created on the cell monolayer to mimic wounds, following the equipment manual's instructions. Time-lapse images of the scratches were captured at specific intervals (e.g., 0 hours, 12 hours, 24 hours, etc.) using the CytationTM 5 Cell Imaging Multi-Mode Reader. Subsequently, image analysis software was employed to quantify the closure of the wounds at each time point. Statistical analysis was performed to compare the wound closure rates at different time points, and the results were presented graphically.

Results

The overall workflow of this study is shown in **Figure 1**. Of the proteins assessed, we were able to develop prediction models for 1,864 proteins with a prediction performance $R^2 \geq 0.01$. In the external validation step, 1,389 of them further demonstrated a correlation coefficient of ≥ 0.1 for predicted expression and measured expression levels. Of such proteins, we observed significant associations between genetically predicted expression levels of 40 proteins and PDAC risk at a false discovery rate (FDR) *p*-value of ≤ 0.05 (**Figure 2, Tables 1 and 2**). Of the associated proteins, 16 are novel ones that have not been reported in previous studies (**Table 1**). Positive associations were observed for 10 of these proteins, and inverse associations were observed for six proteins (**Table 1**). The other 24 associated proteins have been previously reported in our study using pQTL as instruments (28) (**Table 2**). These include 10 that demonstrated positive associations and 14 that showed inverse associations.

For the other proteins that were reported in our previous study using pQTL as instruments (28), while did not show a significant association after FDR correction in the current study (**Supplementary Table 1**), except for sTie-2, the directions of effect were consistent in the

current study compared with those in the published work. Among them, for eight proteins, their associations were at $P<0.05$ in the current work using protein genetic prediction models as instruments (**Supplementary Table 1**).

Based on a comparison of exome-sequencing data of tumor tissue and tumor-adjacent normal tissue obtained from 150 TCGA PDAC patients, the somatic level changes of potentially functional variants/mutations were observed in at least one patient for 10 of the 39 genes encoding identified associated proteins (**Supplementary Table 2**). This proportion (10/39=25.64%) is significantly higher (enrichment P value < 0.00001) than the overall observed proportion of potentially functional changes across the genes encoding the proteins tested for association analyses (95/1,218 = 7.80%; here 1,218 represents the number of the genes available in TCGA analysis as part of the genes encoding the 1,389 assessed proteins).

According to the IPA analysis, several cancer-related functions were enriched for the genes encoding our identified proteins (**Supplementary Table 3**). The top canonical pathways identified included IL-15 production ($P=2.21\times10^{-3}$), Heparan Sulfate Biosynthesis (Late Stages) ($P=2.97\times10^{-3}$), Heparan Sulfate Biosynthesis ($P=3.99\times10^{-3}$), Sperm Motility ($P=7.73\times10^{-3}$), and Dermatan Sulfate Biosynthesis (Late Stages) ($P=0.01$) (**Figure 3**). Among the related networks, the top network was cell-to-cell signaling and interaction, cardiovascular system development and function, organismal development (**Supplementary Figure 1**), followed by cancer, organismal injury and abnormalities, respiratory disease, free radical scavenging, cell death and survival, organismal injury and abnormalities, carbohydrate metabolism, small molecule biochemistry, cell cycle, and cancer, cell-to-cell signaling and interaction, cellular assembly and organization. Interactions among identified proteins were investigated based on STRING

database (**Figure 3**). In the network, KDR was predicted to interact with IGF1R, NOTCH1, MET, SEMA6A, ENG, SELP, and SELE.

Based on interrogation using the OpenTargets and DrugBank database, ten of the identified proteins are supported to be relevant to PDAC (overall score >0 in OpenTargets) and are targets of existing drugs approved to be used to treat human conditions (**Table 3**). Our work indicates potential drug repurposing opportunities of these drug targets to other indications. The scores of molecular docking between each of the proteins and the corresponding meta-drug agents were included in **Table 3**.

Among the 16 novel associated proteins, analysis of TGCA data also revealed potential relevance of B4GT1 and GOLM1 with tumor development (data not shown). Consequently, these two proteins were selected as the targets for experimental validation to further investigate their potential roles in PDAC development. Two gene-specific siRNAs (siGOML1 and siB4GAL1) were employed for post-transcriptional gene silencing of *GOML1* and *B4GAL1*, resulting in the knockdown of these two genes. As depicted in **Figure 4A**, qPCR analysis demonstrated a significant reduction in the mRNA expression of *GOML1* and *B4GAL1* in PANC-1 and SU.86.86 cells at 72 hours after transfection with siGOML1 or siB4GAL1 (40 nM) when compared with the untreated control group ($P < 0.05$). No significant difference was observed between the negative control group (NC, Mock-siRNA transfection) and the control groups (**Figure 4A**). This trend was also consistent in the western blot analysis (**Figure 4B**) in comparison with the qPCR assay, indicating that siGOML1 and siB4GAL1 effectively reduce the expression of *GOML1* and *B4GAL1* at both mRNA and protein levels in PANC-1 and SU.86.86 cells.

To assess the biological impact of *GOLM11* and *B4GAL1* silencing in PANC-1 and SU.86.86 cells, cell proliferation was examined using the MTS assay over a span of five

consecutive days. As shown in **Figures 4C** and **4D**, transfection of siGOML1 and siB4GAL1 inhibited cell proliferation in both PANC-1 and SU.86.86 cells compared with the control (untransfected) and NC (Mock-siRNA transfected) groups. Furthermore, a wound healing assay demonstrated that at 12- and 24-hours post-scratch treatment, the open wound area in *GOLM11* and *B4GAL1* siRNA-transfected cells was significantly larger than that in mock siRNA-transfected or untransfected cells (**Figure 4D, 4E**), implying that knockdown of *GOLM11* and *B4GAL1* in PANC-1 and SU.86.86 cells effectively inhibited cell migration *in vitro*. To investigate whether the down-regulation of *GOLML1* and *B4GAL1* affects the invasive capabilities of PANC-1 and SU.86.86 cells, a transwell analysis was performed. The results revealed a significant inhibition of cell invasion in PANC-1 and SU.86.86 cells upon *GOLML1* or *B4GAL1* silencing. The number of siGOML1 or siB4GAL1-transfected cells invading through the membrane was markedly lower than that of control-siRNA transfected cells (**Fig. 4F**, $P < 0.05$). Together, our findings suggest that GOLM1 and B4GT1 play crucial roles in PDAC cell proliferation, migration, and invasion, and their suppression could potentially serve as a therapeutic strategy for PDAC.

Discussion

This is the first PWAS study using comprehensive protein genetic prediction models to assess the associations between genetically predicted circulating protein concentrations and PDAC risk. Overall, we identified 40 proteins that were significantly associated with PDAC risk after FDR correction, including 16 novel proteins that have not been previously reported. Our results suggest new knowledge on the genetics and etiology of PDAC, and the newly identified proteins could serve as candidate blood biomarkers for risk assessment of PDAC, a highly fatal

malignancy. We also identified potential drug repurposing opportunities targeting the identified proteins which warrant further investigations.

In previous studies, blood concentrations of specific proteins such as CA242, PIVKA-II, PAM4, S100A6, OPN, RBM6, EphA2, and OPG have been reported to be potentially associated with PDAC risk (4-7). In the INTERVAL dataset, proteins S100A6 and OPG were captured, and we were able to develop satisfactory prediction models for their levels in blood (17). We observed a significant association with the same direction for OPG (*P*-value = 0.03, Z-score = 2.23) but not for S100A6 (*P*-value=0.93) with PDAC risk. Such inconsistent findings with previous studies might be explained by potential biases in previous epidemiological studies and warrant further exploration.

In this large study, we identified 16 novel proteins that were associated with PDAC risk. Previous studies have suggested potential roles for some of the novel proteins in pancreatic tumorigenesis. Tie1 deficiency is reported to induce endothelial–mesenchymal transition (EndMT) and promote a motile phenotype (29). EndMT is known to present in human pancreatic tumors (29). Another study reports that TNF-α that is abundantly present in PDAC, induces EndMT and acts at least partially through TIE1 regulation in murine pancreatic tumors (30). For CPB1, immunohistochemistry of tissue microarray from PDAC patients showed that it was significantly downregulated in pancreatic tumor compared with adjacent normal pancreatic tissues (31). This aligns with the negative association between genetically predicted levels of carboxypeptidase B1 and PDAC risk observed in this study. In another study it was reported that mutations in *CPB1* were associated with pancreatic cancer (32). Regarding GOLM1, one study supported that long non-coding RNA TP73-AS1 could promote pancreatic cancer progression through GOLM1 upregulation by competitively binding to miR-128-3p (33). Further

investigations are warranted to clarify roles of the identified proteins in pancreatic cancer development.

Based on drug repurposing analyses, we prioritized several drugs that may serve as promising candidates for treating PDAC, such as Crizotinib, Cabozantinib, Brigatinib, Capmatinib, Tepotinib, and Tivozanib targeting Met. Previous research has supported potential link between these drugs and PDAC. For example, earlier research found that Crizotinib and Cabozantinib could decrease PDAC cell line viability *in vitro* (34). Cabozantinib together with photodynamic therapy had been shown to achieve local control and decrease in tumor metastases in preclinical PDAC models (35). A translational mathematical modeling study revealed that Tepotinib at a dose selection of 500 mg once daily could be effective for PDAC (36). Further work is needed to assess potential efficacy of these drug candidates in PDAC treatment.

There are several strengths of this study for detecting proteins associated with PDAC risk. We developed comprehensive protein genetic prediction models as instruments, which not only potentially minimize biases commonly encountered in conventional observational study design, but also bring improved statistical power compared with the design of only using pQTLs as instruments. However, several limitations of this study need to be recognized when interpreting our findings. First, our results may still be susceptible to potential pleiotropic effects and may not necessarily infer causality. Similar to the design of transcriptome-wide association study (TWAS), our PWAS should be useful for prioritizing causal proteins; however we cannot completely exclude the possibility of false positive findings for some of the identified associations (37). Several likely reasons may induce these, such as correlated protein expression across participants, correlated genetically predicted protein expression, as well as shared genetic variants (37). Future functional investigation will better characterize whether the identified

proteins play a causal role in PDAC development. Second, since in this work the genetically regulated components of plasma protein levels were studied but not the overall measured levels, the utility of the identified proteins as risk biomarkers for PDAC remains unclear. Additional work for measuring circulating protein levels in pre-diagnostic blood samples are needed to evaluate the prediction role of these proteins in PDAC risk. Third, for our current model development design, the candidate predictors for each protein of interest merely rely on the potentially associated SNPs at a specific statistical threshold. A small proportion of proteins were excluded for downstream model construction because of the lack of such SNPs. Future work considering additional potential predictors beyond such statistics-based selection would be needed to improve the ability to evaluate additional proteins. Fourth, previous work has supported that covariates of smoking and body mass index are related to blood protein levels (38,39). In the current study using INTERVAL resources, we were not able to adjust for these covariates during model construction. Further study is thus needed to validate our results. Lastly, the current study largely focuses on Europeans for both protein genetic prediction model development and downstream association analyses with PDAC risk. Future research is warranted to study proteins associated with PDAC risk in other non-European ancestries.

Our TGCA data analysis has revealed potential relevance of B4GT1 and GOLM1 in tumorigenesis and tumor progression. B4GT1 (Beta-1,4-Galactosyl transferase 1) is an enzyme primarily responsible for catalyzing the galactose transfer to specific receptor molecules within organisms (40). Its significance lies in its involvement in various essential biological processes, such as intercellular communication and cell adhesion. Furthermore, alterations in the expression level of B4GT1 have been observed in certain cancers, suggesting its potential implication in tumor initiation and development (41). This intriguing finding has led us to select B4GT1 as a priority

target for further exploration of its role in PDAC using experimental techniques. Similarly, our attention was drawn to GOLM1 (Golgi Membrane Protein 1), a membrane protein predominantly located in the Golgi apparatus, which plays a pivotal role in cellular secretion and transport processes. Recent investigations have demonstrated an upregulation of GOLM1 expression in multiple cancer types, including liver cancer, lung cancer, and pancreatic cancer. Such evidence strongly suggests that GOLM1 might exert a significant influence on the onset and progression of these malignancies (42). Consequently, we selected GOLM1 as an additional focus for verification to gain deeper insights into its involvement in PDAC. By utilizing RNAi technology to silence these genes, our experimental results corroborated the critical roles of GOLM1 and B4GT1 in driving PDAC cell proliferation, migration, and invasion. Subduing these genes holds promise as a potential therapeutic approach for PDAC treatment.

In summary, using protein genetic prediction models, we identified 16 novel protein biomarker candidates for which the genetically predicted circulating levels were significantly associated with PDAC risk. Future work is needed to better characterize the potential roles of these proteins in the etiology of PDAC development, assess the predictive role of such markers in risk assessment of PDAC, and evaluate whether the potential drug repurposing opportunities we identified may improve PDAC outcomes.

Roles of the funders

The funders had no role in the design and conduct of the study; collection, management, analysis, and interpretation of the data; preparation, review, or approval of the manuscript; and decision to submit the manuscript for publication.

Any prior presentations

A preliminary of this work was presented at the American Association for Cancer Research Annual Meeting 2021.

Acknowledgements

The pancreatic cancer genetic datasets used for the association analyses described in this manuscript were obtained from dbGaP at http://www.ncbi.nlm.nih.gov/sites/entrez?db=gap through dbGaP accession phs000206.v5.p3 and phs000648.v1.p1. The authors also would like to thank all the individuals for their participation in the parent studies and all the researchers, clinicians, technicians and administrative staff for their contribution to the studies. This study is supported by the University of Hawaii Cancer Center and V Foundation V Scholar Award. Lang Wu and Chong Wu are supported by NCI R01CA263494. Lang Wu is also supported by NHGRI/NIMHD U54HG013243 and NCI R00CA218892. This work was supported in part by NIH-NIMHD U54MD007598, NIH/NCI1U54CA14393, U56 CA101599-01; Department-of-Defense Breast Cancer Research Program grant BC043180, NIH/NCATS CTSI UL1TR000124 to J.V. Vadgama; Accelerating Excellence in Translational Science Pilot Grants G0812D05, NIH/NCI SC1CA200517 and 9 SC1 GM135050-05 to Y. Wu. Qizhi Yao is supported by VA Merit Award 1 I01 CX001822-01A2 (PI: Yao). The PanScan study was funded in whole or in part with federal funds from the National Cancer Institute (NCI), US National Institutes of Health (NIH) under contract number HHSN261200800001E. Additional support was received from NIH/NCI K07 CA140790, the American Society of Clinical Oncology Conquer Cancer Foundation, the Howard Hughes Medical Institute, the Lustgarten Foundation, the Robert T. and Judith B. Hale Fund for Pancreatic Cancer Research and Promises for Purple. A full list of

acknowledgments for each participating study is provided in the Supplementary Note of the manuscript with PubMed ID: 25086665. For the PanC4 GWAS study, the patients and controls were derived from the following PANC4 studies: Johns Hopkins National Familial Pancreas Tumor Registry, Mayo Clinic Biospecimen Resource for Pancreas Research, Ontario Pancreas Cancer Study (OPCS), Yale University, MD Anderson Case Control Study, Queensland Pancreatic Cancer Study, University of California San Francisco Molecular Epidemiology of Pancreatic Cancer Study, International Agency of Cancer Research and Memorial Sloan Kettering Cancer Center. This work is supported by NCI R01CA154823 Genotyping services were provided by the Center for Inherited Disease Research (CIDR). CIDR is fully funded through a federal contract from the National Institutes of Health to The Johns Hopkins University, contract number HHSN2682011000111. The content is solely the responsibility of the authors and does not necessarily represent the official views of the National Institutes of Health. Participants in the INTERVAL randomised controlled trial were recruited with the active collaboration of NHS Blood and Transplant England (www.nhsbt.nhs.uk), which has supported field work and other elements of the trial. DNA extraction and genotyping were co-funded by the National Institute for Health Research (NIHR), the NIHR BioResource (http://bioresource.nihr.ac.uk) and the NIHR Cambridge Biomedical Research Centre (BRC-1215-20014) [*]. The academic coordinating centre for INTERVAL was supported by core funding from the: NIHR Blood and Transplant Research Unit in Donor Health and Genomics (NIHR BTRU-2014-10024), UK Medical Research Council (MR/L003120/1), British Heart Foundation (SP/09/002; RG/13/13/30194; RG/18/13/33946) and NIHR Cambridge BRC (BRC-1215-20014) [*]. A complete list of the investigators and contributors to the INTERVAL trial is

provided in reference [**]. The academic coordinating centre would like to thank blood donor centre staff and blood donors for participating in the INTERVAL trial.

*The views expressed are those of the author(s) and not necessarily those of the NIHR or the Department of Health and Social Care.

**Di Angelantonio E, Thompson SG, Kaptoge SK, Moore C, Walker M, Armitage J, Ouwehand WH, Roberts DJ, Danesh J, INTERVAL Trial Group. Efficiency and safety of varying the frequency of whole blood donation (INTERVAL): a randomised trial of 45 000 donors. Lancet. 2017 Nov 25;390(10110):2360-2371.

Data sharing statement

The pancreatic cancer genetic datasets used for the association analyses described in this manuscript can be obtained from dbGaP at http://www.ncbi.nlm.nih.gov/sites/entrez?db=gap through dbGaP accession phs000206.v5.p3 and phs000648.v1.p1. The INTERVAL individual-level genotype and protein data, and full summary association results from the genetic analysis, are available through the European Genotype Archive (accession number EGAS00001002555). Summary association results are also publicly available at http://www.phpc.cam.ac.uk/ceu/proteins/, through PhenoScanner (http://www.phenoscanner.medschl.cam.ac.uk) and from the NHGRI-EBI GWAS Catalog (https://www.ebi.ac.uk/gwas/downloads/summary-statistics).

Figure legends

Figure 1. The overall design of this study.

Figure 2. Manhattan plot of 40 identified proteins associated with PDAC risk. Proteins with blue color represent those identified in our previous work using pQTL as instruments, and proteins with red color represent novel ones identified in the current study.

Figure 3. PPI network and canonical pathways of 40 identified proteins associated with PDAC risk. Network nodes represent proteins; edge thickness is proportional to the evidence for the PPI; and dashed lines represent the interaction among clusters. The enrichment of canonical pathways was determined using IPA software.

Figure 4. The analysis of cell proliferation, migration and invasion on PANC-1 and SU.86.86 cells with siB4GLAT1 and siGOLM1 transfection. The quantitative real-time PCR (qPCR) assay and the western blot assay (A) were used to investigate the RNAi effect of siB4GLAT1 and siGOLM1 (40 nM, 72 h) in PANC-1 and SU.86.86 cells. GAPDH were used as an internal control for qPCR analyses and western blot analyses, respectively (B,C) The effect of transfection with siB4GLAT1 and siGOLM1 (40 nM) on cell proliferation. The cells were detected by MTS [3-(4,5-dimethylthiazol-2-yl)-5-(3-carboxymethoxyphenyl)-2-(4-sulfophenyl)-2H-tetrazolium] assay on each day for 5 consecutive days. (D,E) Silencing of *B4GLAT1* and *GOLM1* inhibited migration of PANC-1 and SU.86.86 cells. Representative images of wound scratch assay performed to evaluate the motility of cells after silencing *B4GLAT1* and *GOLM1*. After transfection, a scratch was made on cells monolayer and was monitored with microscopy every 12 hours (0, 12, and 24 h). Bar graphs show normalized wound area, calculated using Gen

5. Representative images of invasion assay. Data are represented as mean ± SD from triplicate samples, where $*p < 0.01$ compared to the control. (F) Effect of siB4GLAT1 and siGOLM1 transfection on the invasion of PANC-1 and SU.86.86 cells. After siB4GLAT1 and siGOLM1 transfection for 48 h, invasive ability of PANC-1 and SU.86.86 cells was identified by transwell assay. $^{**}P < 0.01$ compared with the control cells; $^{\#\#}P < 0.01$ compared with the mock cells; data are expressed as the mean ± SD, n = 3.

References

1. Sung H, Ferlay J, Siegel RL, Laversanne M, Soerjomataram I, Jemal A, *et al.* Global Cancer Statistics 2020: GLOBOCAN Estimates of Incidence and Mortality Worldwide for 36 Cancers in 185 Countries. CA Cancer J Clin **2021**;71(3):209-49 doi 10.3322/caac.21660.
2. Rawla P, Sunkara T, Gaduputi V. Epidemiology of Pancreatic Cancer: Global Trends, Etiology and Risk Factors. World J Oncol **2019**;10(1):10-27 doi 10.14740/wjon1166.
3. Ballehaninna UK, Chamberlain RS. The clinical utility of serum CA 19-9 in the diagnosis, prognosis and management of pancreatic adenocarcinoma: An evidence based appraisal. J Gastrointest Oncol **2012**;3(2):105-19 doi 10.3978/j.issn.2078-6891.2011.021.
4. Tartaglione S, Pecorella I, Zarrillo SR, Granato T, Viggiani V, Manganaro L, *et al.* Protein Induced by Vitamin K Absence II (PIVKA-II) as a potential serological biomarker in pancreatic cancer: a pilot study. Biochem Med (Zagreb) **2019**;29(2):020707 doi 10.11613/BM.2019.020707.
5. Duan B, Hu X, Fan M, Xiong X, Han L, Wang Z, *et al.* RNA-Binding Motif Protein 6 is a Candidate Serum Biomarker for Pancreatic Cancer. Proteomics Clin Appl **2019**;13(5):e1900048 doi 10.1002/prca.201900048.
6. Koshikawa N, Minegishi T, Kiyokawa H, Seiki M. Specific detection of soluble EphA2 fragments in blood as a new biomarker for pancreatic cancer. Cell Death Dis **2017**;8(10):e3134 doi 10.1038/cddis.2017.545.
7. Loosen SH, Neumann UP, Trautwein C, Roderburg C, Luedde T. Current and future biomarkers for pancreatic adenocarcinoma. Tumour Biol **2017**;39(6):1010428317692231 doi 10.1177/1010428317692231.
8. Didelez V, Sheehan N. Mendelian randomization as an instrumental variable approach to causal inference. Stat Methods Med Res **2007**;16(4):309-30 doi 10.1177/0962280206077743.
9. Lawlor DA, Harbord RM, Sterne JA, Timpson N, Davey Smith G. Mendelian randomization: using genes as instruments for making causal inferences in epidemiology. Stat Med **2008**;27(8):1133-63 doi 10.1002/sim.3034.
10. Sun BB, Maranville JC, Peters JE, Stacey D, Staley JR, Blackshaw J, *et al.* Genomic atlas of the human plasma proteome. Nature **2018**;558(7708):73-9 doi 10.1038/s41586-018-0175-2.
11. Wu L, Shu X, Bao J, Guo X, Kote-Jarai Z, Haiman CA, *et al.* Analysis of Over 140,000 European Descendants Identifies Genetically Predicted Blood Protein Biomarkers Associated with Prostate Cancer Risk. Cancer Res **2019**;79(18):4592-8 doi 10.1158/0008-5472.CAN-18-3997.
12. Zhu J, Wu C, Wu L. Associations Between Genetically Predicted Protein Levels and COVID-19 Severity. J Infect Dis **2021**;223(1):19-22 doi 10.1093/infdis/jiaa660.
13. Zhu J, O'Mara TA, Liu D, Setiawan VW, Glubb D, Spurdle AB, *et al.* Associations between Genetically Predicted Circulating Protein Concentrations and Endometrial Cancer Risk. Cancers (Basel) **2021**;13(9) doi 10.3390/cancers13092088.

14. Shu X, Bao J, Wu L, Long J, Shu XO, Guo X, *et al.* Evaluation of associations between genetically predicted circulating protein biomarkers and breast cancer risk. Int J Cancer **2020**;146(8):2130-8 doi 10.1002/ijc.32542.
15. Gusev A, Ko A, Shi H, Bhatia G, Chung W, Penninx BW, *et al.* Integrative approaches for large-scale transcriptome-wide association studies. Nat Genet **2016**;48(3):245-52 doi 10.1038/ng.3506.
16. Liu D, Zhou D, Sun Y, Zhu J, Ghoneim D, Wu C, *et al.* A Transcriptome-Wide Association Study Identifies Candidate Susceptibility Genes for Pancreatic Cancer Risk. Cancer Res **2020**;80(20):4346-54 doi 10.1158/0008-5472.CAN-20-1353.
17. Klein AP, Wolpin BM, Risch HA, Stolzenberg-Solomon RZ, Mocci E, Zhang M, *et al.* Genome-wide meta-analysis identifies five new susceptibility loci for pancreatic cancer. Nat Commun **2018**;9(1):556 doi 10.1038/s41467-018-02942-5.
18. Zhu J, Yang Y, Kisiel JB, Mahoney DW, Michaud DS, Guo X, *et al.* Integrating Genome and Methylome Data to Identify Candidate DNA Methylation Biomarkers for Pancreatic Cancer Risk. Cancer Epidemiol Biomarkers Prev **2021**;30(11):2079-87 doi 10.1158/1055-9965.EPI-21-0400.
19. McCarthy S, Das S, Kretzschmar W, Delaneau O, Wood AR, Teumer A, *et al.* A reference panel of 64,976 haplotypes for genotype imputation. Nat Genet **2016**;48(10):1279-83 doi 10.1038/ng.3643.
20. Howie BN, Donnelly P, Marchini J. A flexible and accurate genotype imputation method for the next generation of genome-wide association studies. PLoS Genet **2009**;5(6):e1000529 doi 10.1371/journal.pgen.1000529.
21. Kramer A, Green J, Pollard J, Jr., Tugendreich S. Causal analysis approaches in Ingenuity Pathway Analysis. Bioinformatics **2014**;30(4):523-30 doi 10.1093/bioinformatics/btt703.
22. Szklarczyk D, Gable AL, Nastou KC, Lyon D, Kirsch R, Pyysalo S, *et al.* The STRING database in 2021: customizable protein-protein networks, and functional characterization of user-uploaded gene/measurement sets. Nucleic Acids Res **2021**;49(D1):D605-D12 doi 10.1093/nar/gkaa1074.
23. Koscielny G, An P, Carvalho-Silva D, Cham JA, Fumis L, Gasparyan R, *et al.* Open Targets: a platform for therapeutic target identification and validation. Nucleic Acids Res **2017**;45(D1):D985-D94 doi 10.1093/nar/gkw1055.
24. Wishart DS, Knox C, Guo AC, Shrivastava S, Hassanali M, Stothard P, *et al.* DrugBank: a comprehensive resource for in silico drug discovery and exploration. Nucleic Acids Res **2006**;34(Database issue):D668-72 doi 10.1093/nar/gkj067.
25. Alam MA SH, Deng H-W. A robust kernel machine regression towards biomarker selection in multi-omics datasets of osteoporosis for drug discovery. In: University T, editor. arXiv2022.
26. Kim S, Chen J, Cheng T, Gindulyte A, He J, He S, *et al.* PubChem 2019 update: improved access to chemical data. Nucleic Acids Res **2019**;47(D1):D1102-D9 doi 10.1093/nar/gky1033.
27. Kim SY, Jeong HH, Kim J, Moon JH, Sohn KA. Robust pathway-based multi-omics data integration using directed random walks for survival prediction in multiple cancer studies. Biol Direct **2019**;14(1):8 doi 10.1186/s13062-019-0239-8.

28. Zhu J, Shu X, Guo X, Liu D, Bao J, Milne RL, *et al.* Associations between Genetically Predicted Blood Protein Biomarkers and Pancreatic Cancer Risk. Cancer Epidemiol Biomarkers Prev **2020**;29(7):1501-8 doi 10.1158/1055-9965.EPI-20-0091.
29. Garcia J, Sandi MJ, Cordelier P, Binetruy B, Pouyssegur J, Iovanna JL, *et al.* Tie1 deficiency induces endothelial-mesenchymal transition. EMBO Rep **2012**;13(5):431-9 doi 10.1038/embor.2012.29.
30. Adjuto-Saccone M, Soubeyran P, Garcia J, Audebert S, Camoin L, Rubis M, *et al.* TNF-alpha induces endothelial-mesenchymal transition promoting stromal development of pancreatic adenocarcinoma. Cell Death Dis **2021**;12(7):649 doi 10.1038/s41419-021-03920-4.
31. Song Y, Wang Q, Wang D, Junqiang L, Yang J, Li H, *et al.* Label-Free Quantitative Proteomics Unravels Carboxypeptidases as the Novel Biomarker in Pancreatic Ductal Adenocarcinoma. Transl Oncol **2018**;11(3):691-9 doi 10.1016/j.tranon.2018.03.005.
32. Tamura K, Yu J, Hata T, Suenaga M, Shindo K, Abe T, *et al.* Mutations in the pancreatic secretory enzymes CPA1 and CPB1 are associated with pancreatic cancer. Proc Natl Acad Sci U S A **2018**;115(18):4767-72 doi 10.1073/pnas.1720588115.
33. Wang B, Sun X, Huang KJ, Zhou LS, Qiu ZJ. Long non-coding RNA TP73-AS1 promotes pancreatic cancer growth and metastasis through miRNA-128-3p/GOLM1 axis. World J Gastroenterol **2021**;27(17):1993-2014 doi 10.3748/wjg.v27.i17.1993.
34. Escorcia FE, Houghton JL, Abdel-Atti D, Pereira PR, Cho A, Gutsche NT, *et al.* ImmunoPET Predicts Response to Met-targeted Radioligand Therapy in Models of Pancreatic Cancer Resistant to Met Kinase Inhibitors. Theranostics **2020**;10(1):151-65 doi 10.7150/thno.37098.
35. Broekgaarden M, Alkhateeb A, Bano S, Bulin AL, Obaid G, Rizvi I, *et al.* Cabozantinib Inhibits Photodynamic Therapy-Induced Auto- and Paracrine MET Signaling in Heterotypic Pancreatic Microtumors. Cancers (Basel) **2020**;12(6) doi 10.3390/cancers12061401.
36. Xiong W, Friese-Hamim M, Johne A, Stroh C, Klevesath M, Falchook GS, *et al.* Translational pharmacokinetic-pharmacodynamic modeling of preclinical and clinical data of the oral MET inhibitor tepotinib to determine the recommended phase II dose. CPT Pharmacometrics Syst Pharmacol **2021**;10(5):428-40 doi 10.1002/psp4.12602.
37. Wainberg M, Sinnott-Armstrong N, Mancuso N, Barbeira AN, Knowles DA, Golan D, *et al.* Opportunities and challenges for transcriptome-wide association studies. Nature genetics **2019**;51(4):592-9.
38. Madhuvanthi M, Lathadevi GV. Serum Proteins Alteration in Association with Body Mass Index in Human Volunteers. J Clin Diagn Res **2016**;10(6):CC05-7 doi 10.7860/JCDR/2016/18278.8047.
39. Gallus S, Lugo A, Suatoni P, Taverna F, Bertocchi E, Boffi R, *et al.* Effect of Tobacco Smoking Cessation on C-Reactive Protein Levels in A Cohort of Low-Dose Computed Tomography Screening Participants. Sci Rep **2018**;8(1):12908 doi 10.1038/s41598-018-29867-9.
40. Morokuma D, Xu J, Hino M, Mon H, Merzaban JS, Takahashi M, *et al.* Expression and Characterization of Human beta-1, 4-Galactosyltransferase 1 (beta4GalT1) Using Silkworm-Baculovirus Expression System. Mol Biotechnol **2017**;59(4-5):151-8 doi 10.1007/s12033-017-0003-1.

41. Cui Y, Li J, Zhang P, Yin D, Wang Z, Dai J, *et al.* B4GALT1 promotes immune escape by regulating the expression of PD-L1 at multiple levels in lung adenocarcinoma. J Exp Clin Cancer Res **2023**;42(1):146 doi 10.1186/s13046-023-02711-3.
42. Liu Y, Hu X, Liu S, Zhou S, Chen Z, Jin H. Golgi Phosphoprotein 73: The Driver of Epithelial-Mesenchymal Transition in Cancer. Front Oncol **2021**;11:783860 doi 10.3389/fonc.2021.783860.

Table 1. Novel proteins with genetically predicted concentrations in plasma to be associated with pancreatic cancer risk

Protein	SOMAmer ID	Protein full name	Protein-encoding gene	Region for protein encoding gene	Prediction model method	Number of Predicting SNPs	Number of Predicting SNPs-Cis*	Number of Predicting SNPs-Trans	Model internal cross validation R^2	Model external validation R^2	Z-value[a]	*P*-value[a]	FDR *P*-value[b]
IL-23 R	IL23R.5088.175.3	Interleukin-23 receptor	*IL23R*	1p31.3	elastic net	24	24	0	0.04	0.04	3.55	3.80×10^{-4}	0.02
sTie-1	TIE1.2844.53.2	Tyrosine-Protein Kinase Receptor Tie-1, Soluble	*TIE1*	1p34.2	lasso	18	7	11	0.22	0.28	5.67	1.46×10^{-8}	1.22×10^{-6}
FA20B	FAM20B.7198.197.3	Glycosaminoglycan Xylosylkinase	*FAM20B*	1q25.2	lasso	8	5	3	0.02	0.04	5.30	1.17×10^{-7}	7.82×10^{-6}
FAM3D	FAM3D.13102.1.3	Protein FAM3D	*FAM3D*	3p14.2	elastic net	58	16	42	0.37	0.36	6.10	1.07×10^{-9}	1.02×10^{-7}
Carboxypeptidase B1	CPB1.6356.3.3	Carboxypeptidase B	*CPB1*	3q24	lasso	7	3	4	0.04	0.03	-4.55	5.38×10^{-6}	3.00×10^{-4}
RAP	LRPAP1.3640.14.3	alpha-2-macroglobulin receptor-associated protein	*LRPAP1*	4p16.3	elastic net	168	23	145	0.27	0.22	3.21	0.001	0.04
Semaphorin-6A	SEMA6A.7945.10.3	Semaphorin-6A	*SEMA6A*	5q23.1	elastic net	66	44	22	0.05	0.05	-3.57	3.54×10^{-4}	0.02
B4GT1	B4GALT1.13381.49.3	Beta-1,4-galactosyltransferase 1	*B4GALT1*	9p21.1	elastic net	39	16	23	0.08	0.10	4.65	3.29×10^{-6}	1.96×10^{-4}
GOLM1	GOLM1.8983.7.3	Golgi Membrane Protein 1	*GOLM1*	9q21.33	lasso	10	0	10	0.14	0.17	8.07	7.12×10^{-16}	2.14×10^{-13}
QSOX2	QSOX2.8397.147.3	Sulfhydryl oxidase 2	*QSOX2*	9q34.3	elastic net	28	10	18	0.40	0.40	7.98	1.44×10^{-15}	2.75×10^{-13}
KIN17	KIN.14643.27.3	DNA/RNA-binding protein KIN17	*KIN*	10p14	elastic net	29	0	29	0.05	0.07	-5.52	3.31×10^{-8}	2.60×10^{-6}
ISLR2	ISLR2.13124.20.3	Immunoglobulin superfamily containing leucine-rich repeat protein 2	*ISLR2*	15q24.1	elastic net	77	32	45	0.14	0.13	-3.45	5.65×10^{-4}	0.02
DPEP2	DPEP2.8327.26.3	Dipeptidase 2	*DPEP2*	16q22.1	elastic net	36	0	36	0.06	0.05	-4.01	5.97×10^{-5}	0.003
Chymotrypsin	CTRB1.5671.1.3	Chymotrypsinogen B	*CTRB1*	16q23.1	elastic net	85	69	16	0.23	0.24	-4.32	1.59×10^{-5}	8.50×10^{-4}

Laminin	LAMA1.LAMB1.LAMC1.<br>2728.62.2	Laminin	*LAMA1*,<br>*LAMB1*,<br>*LAMC1*	18p11.31,<br>7q31.1,<br>1q25.3	elastic net	62	14	48	0.08	0.05	3.88	1.06×10^{-4}	0.005
TPST2	TPST2.8024.64.3	Protein-Tyrosine Sulfotransferase 2	*TPST2*	22q12.1	elastic net	52	28	24	0.07	0.08	5.88	4.16×10^{-9}	3.71×10^{-7}

***** SNPs within 1MB of the protein-encoding gene

a Associations between genetically predicted protein levels and PDAC risk after adjustment for age, sex, and top 10 principle components.

b FDR *P*-value: false discovery rate (FDR) adjusted *P*-value; associations with a FDR $p\leq0.05$ considered statistically significant

Table 2. Previously reported proteins with genetically predicted concentrations in plasma to be associated with pancreatic cancer risk

Protein	SOMAmer ID	Protein full name	Protein-encoding gene	Region for protein encoding gene	Prediction model method	Number of Predicting SNPs	Number of Predicting SNPs-Cis*	Number of Predicting SNPs-Trans	Model internal cross validation R^2	Model external validation R^2	Z-value[a]	*P*-value[a]	FDR *P*-value[b]
sE-Selectin	SELE.3470.1.2	E-selectin	*SELE*	1q24.2	lasso	6	0	6	0.39	0.44	-7.88	$3.33×10^{-15}$	$5.47×10^{-13}$
P-Selectin	SELP.4154.57.2	P-Selectin	*SELP*	1q24.2	lasso	11	7	4	0.26	0.27	-3.77	$1.66×10^{-4}$	0.008
LMA2L	LMAN2L.8013.9.3	VIP36-like protein	*LMAN2L*	2q11.2	top1	1	1	0	0.03	0.02	3.35	$8.01×10^{-4}$	0.03
Alkaline phosphatase, intestine	ALPI.10463.23.3	Intestinal-type alkaline phosphatase	*ALPI*	2q37.1	lasso	8	0	8	0.03	0.06	-6.79	$1.09×10^{-11}$	$1.21×10^{-9}$
VEGF sR2	KDR.3651.50.5	Vascular endothelial growth factor receptor 2	*KDR*	4q12	elastic net	56	18	38	0.18	0.12	-6.21	$5.22×10^{-10}$	$5.37×10^{-8}$
ADH1B	ADH1B.9834.62.3	Alcohol dehydrogenase 1B	*ADH1B*	4q23	lasso	6	0	6	0.08	0.03	3.21	0.001	0.04
LIF sR	LIFR.5837.49.3	Leukemia inhibitory factor receptor	*LIFR*	5p13.1	top1	1	0	1	0.03	0.02	-7.39	$1.42×10^{-13}$	$1.73×10^{-11}$
gp130, soluble	IL6ST.2620.4.2	Interleukin-6 receptor subunit beta	*IL6ST*	5q11.2	elastic net	51	21	30	0.06	0.05	-3.69	$2.22×10^{-4}$	0.01
GP116	ADGRF5.6409.57.3	Adhesion G protein-coupled receptor F5	*ADGRF5*	6p12.3	lasso	22	15	7	0.46	0.43	-4.65	$3.37×10^{-6}$	$1.96×10^{-4}$
CD36 ANTIGEN	CD36.2973.15.2	Platelet glycoprotein 4	*CD36*	7q21.11	top1	1	0	1	0.03	0.05	3.31	$9.25×10^{-4}$	0.03
Met	MET.2837.3.2	Hepatocyte growth factor receptor	*MET*	7q31	blup	1,668	603	1,065	0.07	0.04	-5.06	$4.27×10^{-7}$	$2.72×10^{-5}$
STOM	STOM.8261.51.3	Erythrocyte band 7 integral membrane protein	*STOM*	9q33.2	lasso	5	0	5	0.11	0.05	3.31	$9.18×10^{-4}$	0.03
BGAT	ABO.9253.52.3	Histo-blood group ABO system transferase	*ABO*	9q34.2	blup	2,473	2,347	126	0.72	0.72	9.18	$4.20×10^{-20}$	$5.62×10^{-17}$
Notch 1	NOTCH1.5107.7.2	Neurogenic locus notch homolog protein 1	*NOTCH1*	9q34.3	top1	1	0	1	0.01	0.02	3.29	$9.97×10^{-4}$	0.04

Endoglin	ENG.4908.6.1	Endoglin	*ENG*	9q34.11	top1	1	0	1	0.01	0.01	-8.04	8.93×10^{-16}	2.14×10^{-13}
ST4S6	CHST15.4469.78.2	Carbohydrate sulfotransferase 15	*CHST15*	10q26.13	lasso	5	1	4	0.05	0.03	-8.62	6.46×10^{-18}	4.32×10^{-15}
	CHST15.14097.86.3				lasso	9	2	7	0.04	0.02	-8.03	9.60×10^{-16}	2.14×10^{-13}
CHSTB	CHST11.7779.86.3	Carbohydrate sulfotransferase 11	*CHST11*	12q23.3	elastic net	69	46	23	0.11	0.07	3.52	4.25×10^{-4}	0.02
THSD1	THSD1.5621.64.3	Thrombospondin type-1 domain-containing protein 1	*THSD1*	13q14.3	elastic net	44	27	17	0.04	0.03	-5.34	9.41×10^{-8}	6.62×10^{-6}
GLCE	GLCE.7808.5.3	D-glucuronyl C5-epimerase	*GLCE*	15q23	lasso	11	6	5	0.36	0.34	4.18	2.94×10^{-5}	0.002
IGF-I sR	IGF1R.4232.19.2	Insulin-like growth factor 1 receptor	*IGF1R*	15q26.3	top1	1	0	1	0.01	0.02	-7.39	1.42×10^{-13}	1.73×10^{-11}
Desmoglein-2	DSG2.9484.75.3	Desmoglein-2	*DSG2*	18q12.1	elastic net	66	44	22	0.04	0.06	5.34	9.18×10^{-8}	6.62×10^{-6}
DC-SIGN	CD209.3029.52.2	CD209 Antigen	*CD209*	19p13.2	elastic net	58	26	32	0.39	0.38	8.52	1.62×10^{-17}	7.22×10^{-15}
IR	INSR.3448.13.2	Insulin receptor	*INSR*	19p13.2	lasso	7	0	7	0.09	0.12	-7.53	4.98×10^{-14}	7.40×10^{-12}

***** SNPs within 1MB of the protein-encoding gene

a Associations between genetically predicted protein levels and PDAC risk after adjustment for age, sex, and top 10 principle components.

b FDR *P*-value: false discovery rate (FDR) adjusted *P*-value; associations with a FDR $p \leq 0.05$ considered statistically significant

Table 3. Drug repurposing opportunities

Protein	Protein full name	Protein-encoding gene	OpenTargets information (overall score)	Drugbank ID	Drug name	Molecular action	Molecular docking score*
sTie-1	Tyrosine-Protein Kinase Receptor Tie-1, Soluble	*TIE1*	0.006	DB12010	Fostamatinib	inhibitor	-6.1
Carboxypeptidase B1	Carboxypeptidase B	*CPB1*	0.159	DB04272	Citric acid	NA	-3.9
Chymotrypsin	Chymotrypsinogen B	*CTRB1*	0.078	DB06692	Aprotinin	NA	MDNA
sE-Selectin	E-selectin	*SELE*	0.023	DB01136	Carvedilol	inhibitor	-6.9
P-Selectin	P-Selectin	*SELP*	0.008	DB01109	Heparin	inhibitor	-4.9
				DB08813	Nadroparin	inhibitor	-4.9
				DB06779	Dalteparin	inhibitor	-4.9
				DB15271	Crizanlizumab	inhibitor	3DSNA
VEGF sR2	Vascular endothelial growth factor receptor 2	*KDR*	0.367	DB06589	Pazopanib	inhibitor	-6.3
				DB08896	Regorafenib	inhibitor	-6.5
				DB09079	Nintedanib	inhibitor	-5.8
				DB14840	Ripretinib	inhibitor	-6.6
				DB00398	Sorafenib	antagonist	-6.6
				DB01268	Sunitinib	inhibitor	-5.6
				DB06595	Midostaurin	antagonist inhibitor	-5.1
				DB06626	Axitinib	inhibitor	-6.0
				DB08875	Cabozantinib	antagonist	**-7.0**
				DB08901	Ponatinib	inhibitor	-6.9
				DB09078	Lenvatinib	inhibitor	-6.1

				DB05578	Ramucirumab	antagonist	3DSNA
				DB12010	Fostamatinib	inhibitor	-5.3
				DB12147	Erdafitinib	substrate	-5.5
				DB15822	Pralsetinib	inhibitor	-6.9
				DB11800	Tivozanib	inhibitor	-6.4
ADH1B	Alcohol dehydrogenase 1B	*ADH1B*	0.001	DB00898	Ethanol	substrate	-2.8
				DB09462	Glycerin	NA	-3.7
				DB00157	NADH	substrate	**-9.6**
				DB01213	Fomepizole	inhibitor	-3.9
Met	Hepatocyte growth factor receptor	*MET*	0.304	DB08865	Crizotinib	inhibitor	**-8.1**
				DB08875	Cabozantinib	antagonist	**-8**
				DB12267	Brigatinib	inhibitor	**-8.2**
				DB12010	Fostamatinib	inhibitor	-6.7
				DB11791	Capmatinib	inhibitor	**-8.7**
				DB15133	Tepotinib	inhibitor	**-8.3**
				DB11800	Tivozanib	inhibitor	**-8.2**
				DB16695	Amivantamab	antagonist antibody	3DSNA
IGF-I sR	Insulin-like growth factor 1 receptor	*IGF1R*	0.099	DB00071	Insulin pork	NA	MDNA
				DB00046	Insulin lispro	activator	MDNA
				DB01307	Insulin detemir	activator	MDNA
				DB00047	Insulin glargine	activator	MDNA
				DB01306	Insulin aspart	activator	MDNA
				DB01309	Insulin glulisine	activator	MDNA
				DB09564	Insulin degludec	activator	MDNA

				DB14751	Mecasermin rinfabate	agonist	MDNA
				DB09456	Insulin beef	activator	MDNA
				DB08804	Nandrolone decanoate	inducer	-5.8
				DB01277	Mecasermin	agonist	3DSNA
				DB00030	Insulin human	activator	MDNA
				DB06343	Teprotumumab	binder, antibody	3DSNA
				DB12267	Brigatinib	inhibitor	-5.7
IR	Insulin receptor	*INSR*	0.013	DB00047	Insulin glargine	agonist	MDNA
				DB00071	Insulin pork	binder	MDNA
				DB01307	Insulin detemir	agonist	MDNA
				DB00046	Insulin lispro	agonist	MDNA
				DB01306	Insulin aspart	agonist	MDNA
				DB01309	Insulin glulisine	agonist	MDNA
				DB09564	Insulin degludec	agonist	MDNA
				DB09129	Chromic chloride	activator	MDNA
				DB14751	Mecasermin rinfabate	NA	MDNA
				DB09456	Insulin beef	agonist	MDNA
				DB00030	Insulin human	agonist	MDNA
				DB01277	Mecasermin	NA	3DSNA
				DB12267	Brigatinib	binding	**-8.4**
				DB12010	Fostamatinib	inhibitor	**-7.5**

* a score of ≤-7 represents a good interaction between the protein and corresponding drug agent and is bolded.

MDNA: Molecular docking not applicable

3DSNA: 3D structure not available.

Figure 1

Click here to access/download;Figure;Figure 1.pdf

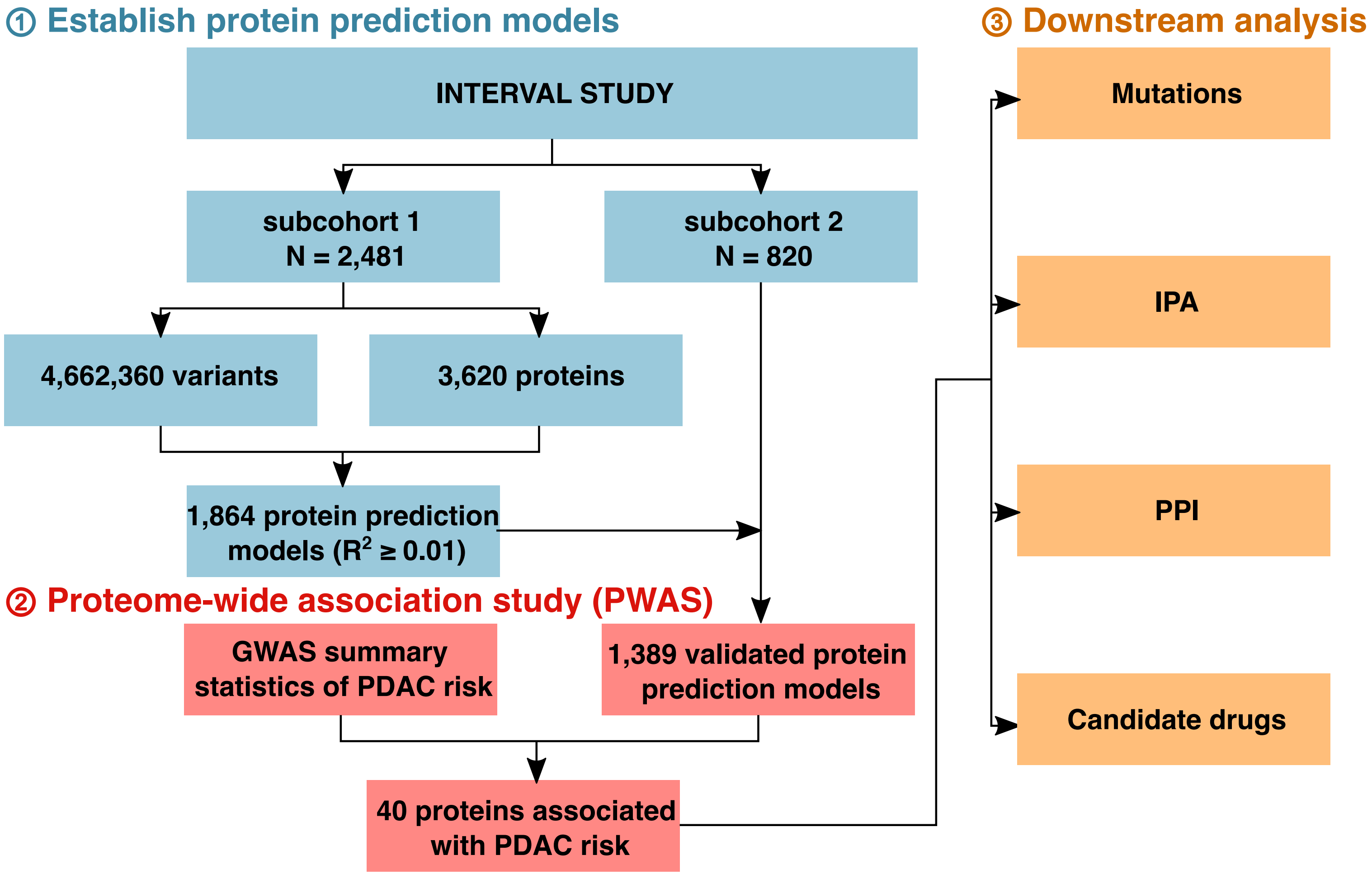

Figure 2

Click here to access/download;Figure;Figure 2.pdf

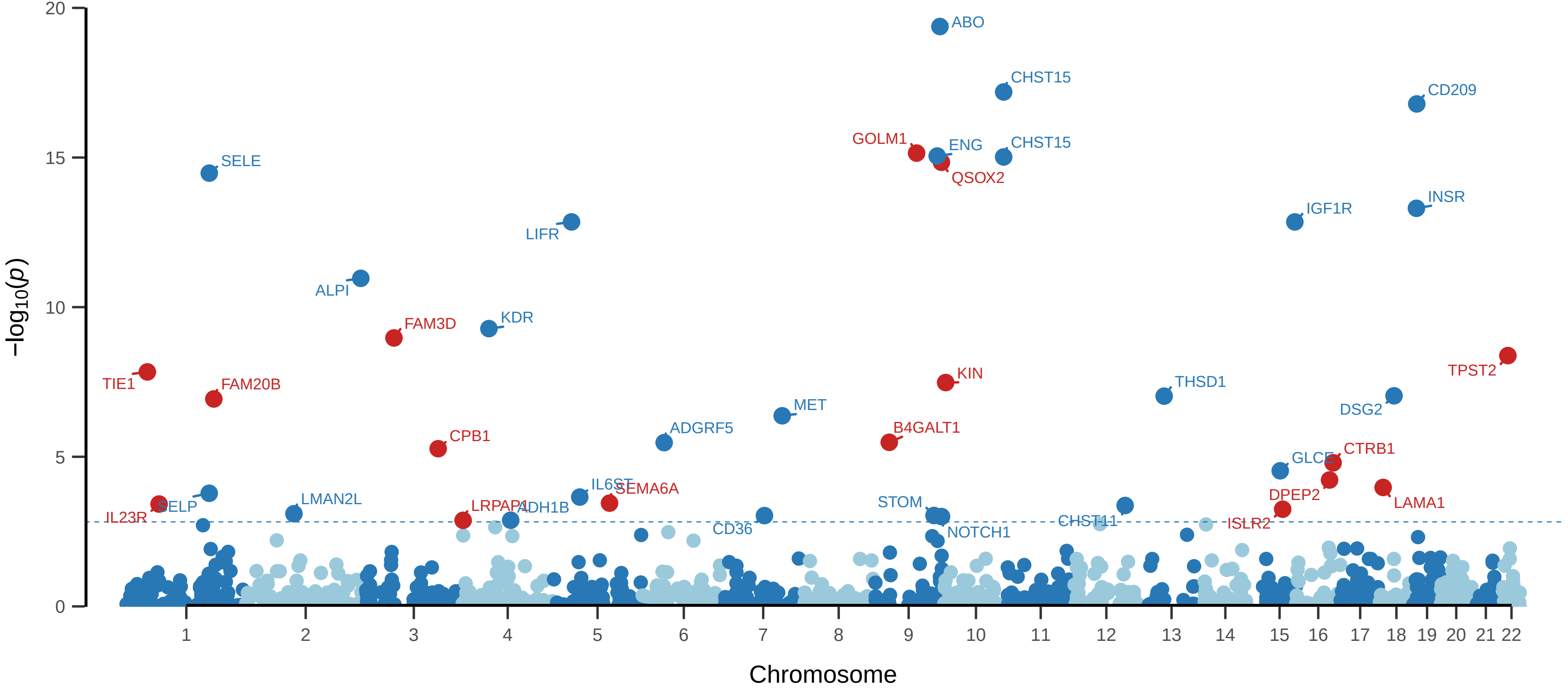

Figure 3

Click here to access/download;Figure;Figure 3.pdf

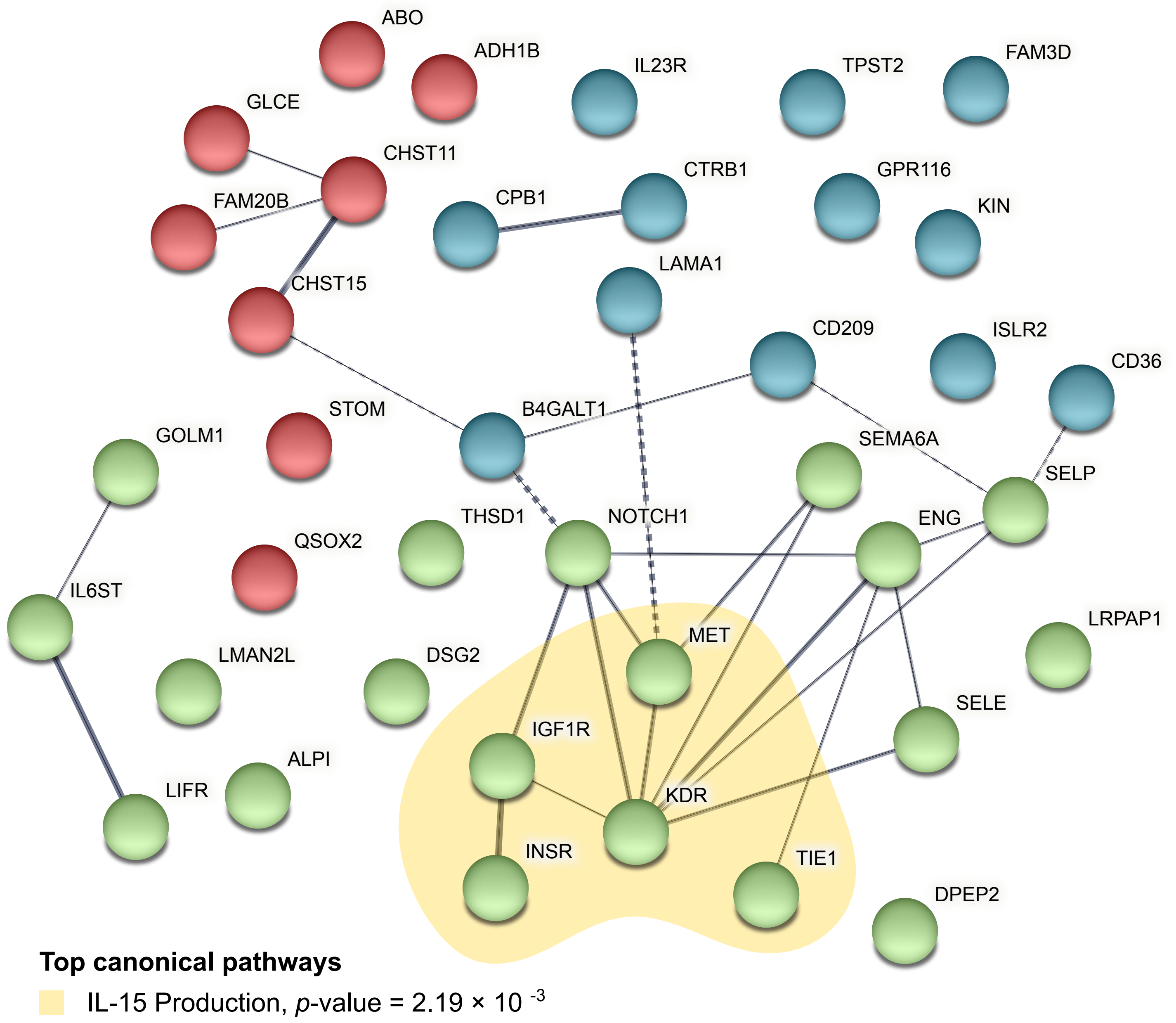

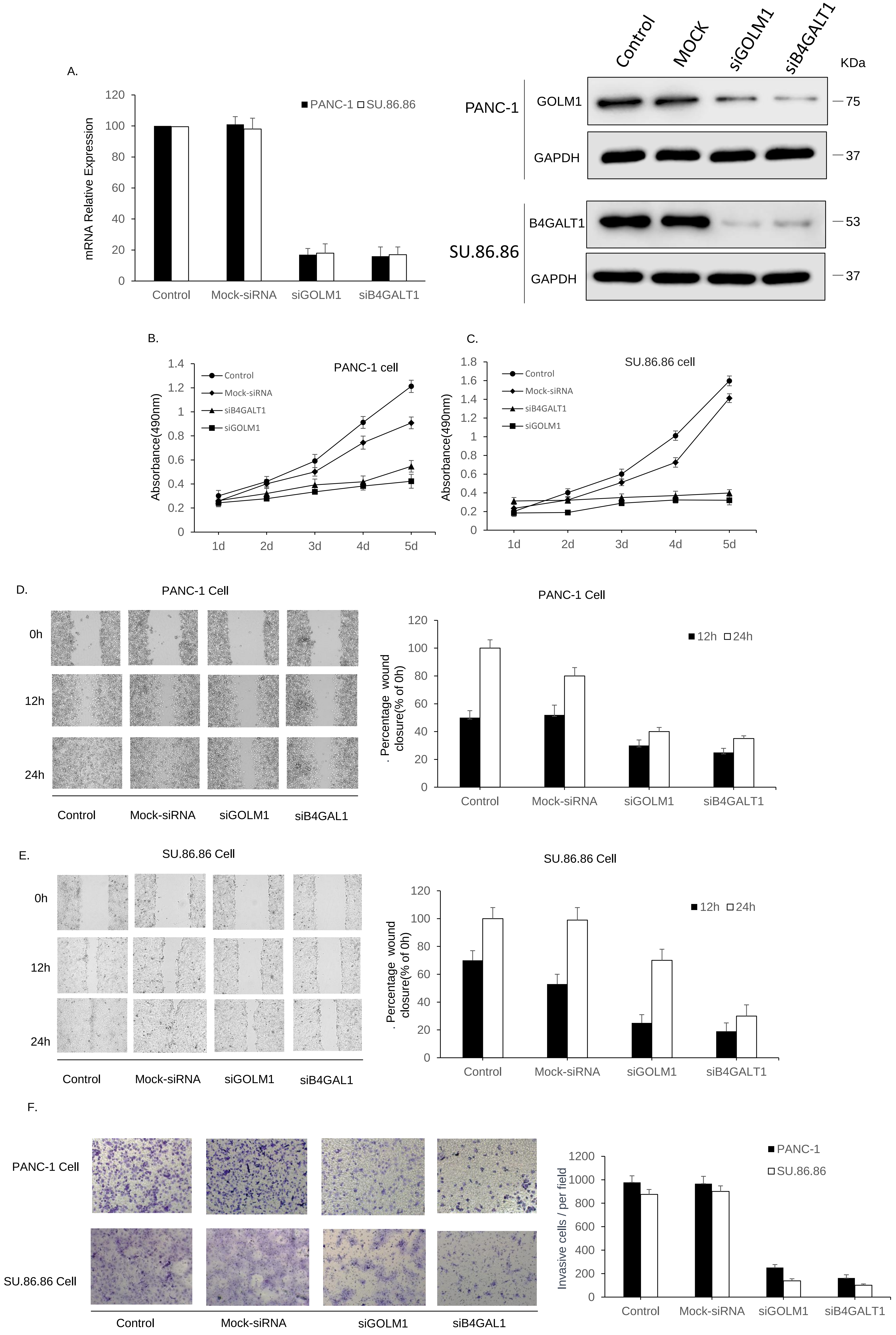
A.
mRNA Relative Expression
PANC-1 SU.86.86
Control
Mock-siRNA
siGOLM1
siB4GALT1
Control MOCK siGOLM1 siB4GALT1
KDa
PANC-1
GOLM1
75
GAPDH
37
SU.86.86
B4GALT1
53
GAPDH
37
B.
PANC-1 cell
Absorbance(490nm)
Control
Mock-siRNA
siB4GALT1
siGOLM1
1d 2d 3d 4d 5d
C.
SU.86.86 cell
D.
PANC-1 Cell
0h
12h
24h
Control Mock-siRNA siGOLM1 siB4GAL1
. Percentage wound closure(% of 0h)
12h 24h
Control Mock-siRNA siGOLM1 siB4GALT1
E.
SU.86.86 Cell
F.
PANC-1 Cell
SU.86.86 Cell
Control Mock-siRNA siGOLM1 siB4GAL1
Invasive cells / per field
PANC-1
SU.86.86

Click here to access/download
Supplementary Material
Supplementary File.docx

Personal Cover Click here to access/download;Personal Cover;cover letter.docx

UNIVERSITY OF HAWAI'I
CANCER CENTER

October 23, 2023

Dr. Scott Edmunds
Editor-in-Chief, *GigaScience*

Proteome-wide association study and functional validation identify novel protein markers for pancreatic ductal adenocarcinoma

Dear Dr. Edmunds:

My colleagues and I would like to submit this manuscript for publication consideration in *GigaScience.*

As you know, pancreatic ductal adenocarcinoma (PDAC) is a lethal malignancy with few known risk factors and biomarkers. Identifying biomarkers is critical for understanding the pathogenesis of this deadly cancer and developing novel therapeutic approaches. Several blood protein biomarkers have been reported to be linked to PDAC in previous studies, however, findings are often inconsistent, potentially due to common biases existing in the conventional epidemiologic study design. One alternative study design is to use genetic instruments to identify proteins whose genetically predicted levels in blood are associated with PDAC risk. This is a design similar to the popular transcriptome-wide association study (TWAS), but focusing on protein expression levels, a novel design that is rarely explored. It is challenging to construct satisfactory genetic perdition models for protein expression levels, because there are far more pQTLs in trans regions than in cis regions.

In this study, we applied a highly novel study to develop comprehensive protein genetic prediction models by considering both cis- and trans-acting elements as instruments for identifying novel PDAC related proteins. We leveraged genome and plasma proteome data of 2,481 healthy European descendants included in the INTERVAL study to establish such prediction models. We selected models with a prediction performance of >0.01 in both internal and external validation for association analyses with PDAC risk, by analyzing 8,275 cases and 6,723 controls of European descent from the Pancreatic Cancer Cohort Consortium and the Pancreatic Cancer Case-Control Consortium.

We identified significant associations between predicted concentrations of 40 proteins and PDAC risk at a false discovery rate of < 0.05, including 16 novel proteins. For 29 of the genes encoding identified proteins, somatic level potentially functional mutations were detected in PDAC patients in The Cancer Genome Atlas. Relevant protein-encoding genes were also significantly enriched in several cancer-related pathways. We further identified drugs targeting

the identified proteins, which may serve as candidates for drug repurposing for treating PDAC. We also silenced two of the novel protein-encoding genes and observed critical roles of *GOLM1* and *B4GALT1* in driving PDAC cell proliferation, migration, and invasion, by testing two independent cell lines. Our functional characterization further supported critical roles of identified novel proteins in pancreatic tumorigenesis.

We believe that our manuscript should be of great interest to the scientific community served by *GigaScience.* In particular, our study could serve as an excellent model for future research that integrates large genomics and proteomics data to understand the genetics and biology of diseases in the post-GWAS era. We hope that you will find our work interesting and would be willing to consider it for publication in your journal.

Sincerely,

Lang Wu, Ph.D.
Director, Pacific Center for Genome Research
Associate Professor, University of Hawaii Cancer Center
University of Hawaii
Email: lwu@cc.hawaii.edu
