# Supplementary material for: Proteome-wide association study and functional validation identify novel protein markers for pancreatic ductal adenocarcinoma
Source: Gigascience. 2024 Apr 12;13:giae012. doi: 10.1093/gigascience/giae012 (PMC11010651; doi:10.1093/gigascience/giae012)
Supplement: giae012_GIGA-D-23-00321_Revision_1 [file giae012_giga-d-23-00321_revision_1.pdf]

# GigaScience

## Proteome-wide association study and functional validation identify novel protein markers for pancreatic ductal adenocarcinoma

--Manuscript Draft--

| | |
|---|---|
| **Manuscript Number:** | GIGA-D-23-00321R1 |
| **Full Title:** | Proteome-wide association study and functional validation identify novel protein markers for pancreatic ductal adenocarcinoma |
| **Article Type:** | Research |
| **Funding Information:** | |


| Funder | Recipient |
|---|---|
| University of Hawaii Cancer Center and V Foundation V Scholar Award | Not applicable |
| National Cancer Institute (R01CA263494) | Dr. Chong Wu<br>Associated Professor Lang Wu |
| National Human Genome Research Institute (U54HG013243) | Associated Professor Lang Wu |
| National Cancer Institute (R00CA218892) | Associated Professor Lang Wu |
| National Institute on Minority Health and Health Disparities (U54MD007598) | Not applicable |
| NIH/NCI (1U54CA14393) | Not applicable |
| NIH/NCI (U56 CA101599-01) | Not applicable |
| Department-of-Defense Breast Cancer Research Program (BC043180) | Dr. Jaydutt V. Vadgama |
| NIH/NCATS (CTSI UL1TR000124) | Dr. Jaydutt V. Vadgama |
| Accelerating Excellence in Translational Science Pilot Grants (G0812D05) | Dr. Yong Wu |
| NIH/NCI (SC1CA200517) | Dr. Yong Wu |
| NIH/NCI (9 SC1 GM135050-05) | Dr. Yong Wu |
| VA Merit Award (1 I01 CX001822-01A2) | Dr. Qizhi Yao |
| National Cancer Institute (NCI), US National Institutes of Health (NIH) (HHSN261200800001E) | Not applicable |
| NIH/NCI (K07 CA140790) | Not applicable |
| the American Society of Clinical Oncology Conquer Cancer Foundation | Not applicable |
| the Howard Hughes Medical Institute | Not applicable |
| the Lustgarten Foundation | Not applicable |
| he Robert T. and Judith B. Hale Fund for Pancreatic Cancer Research | Not applicable |
| Promises for Purple | Not applicable |
| NCI (R01CA154823) | Not applicable |
| National Institutes of Health to The Johns Hopkins University (HHSN2682011000111) | Not applicable |
| National Institute for Health Research (NIHR) | Not applicable |
| NIHR BioResource | Not applicable |


*Powered by Editorial Manager® and ProduXion Manager® from Aries Systems Corporation*

| | | |
|---|---|---|
| | NIHR Cambridge Biomedical Research Centre (BRC-1215-20014) | Not applicable |
| | NIHR Blood and Transplant Research Unit in Donor Health and Genomics (NIHR BTRU-2014-10024) | Not applicable |
| | UK Medical Research Council (MR/L003120/1) | Not applicable |
| | British Heart Foundation (SP/09/002; RG/13/13/30194; RG/18/13/33946) | Not applicable |
| | NIHR Cambridge BRC (BRC-1215-20014) | Not applicable |

**Abstract:**

Abstract
Pancreatic ductal adenocarcinoma (PDAC) remains a lethal malignancy, largely due to the paucity of reliable biomarkers for early detection and therapeutic targeting. Existing blood protein biomarkers for PDAC often suffer from replicability issues, arising from inherent limitations such as unmeasured confounding factors in conventional epidemiologic study designs. To circumvent these limitations, we use genetic instruments to identify proteins with genetically predicted levels to be associated with PDAC risk. Leveraging genome and plasma proteome data from the INTERVAL study, we established and validated models to predict protein levels using genetic variants. By examining 8,275 PDAC cases and 6,723 controls, we identified 40 associated proteins, of which 16 are novel. Functionally validating these candidates by focusing on two selected novel protein-encoding genes, GOLMA1 and B4GALT1, we demonstrated their pivotal roles in driving PDAC cell proliferation, migration, and invasion. Furthermore, we also identified potential drug repurposing opportunities for treating PDAC.
Significance:
PDAC is a notoriously difficult-to-treat malignancy, and our limited understanding of causal protein markers hampers progress in developing effective early detection strategies and treatments. Our study identifies novel causal proteins using genetic instruments and subsequently functionally validates selected novel proteins. This dual approach enhances our understanding of PDAC etiology and potentially opens new avenues for therapeutic interventions.
Keywords: Biomarkers, protein, genetics, pancreatic cancer, risk

| | |
|---|---|
| **Corresponding Author:** | Lang Wu<br>University of Hawai'i at Manoa<br>Honolulu, UNITED STATES |
| **Corresponding Author Secondary Information:** | |
| **Corresponding Author's Institution:** | University of Hawai'i at Manoa |
| **Corresponding Author's Secondary Institution:** | |
| **First Author:** | Jingjing Zhu |
| **First Author Secondary Information:** | |
| **Order of Authors:** | Jingjing Zhu |
| | Ke Wu |
| | Shuai Liu |
| | Alexandra Masca |
| | Hua Zhong |
| | Tai Yang |
| | Dalia H Ghoneim |
| | Praveen Surendran |
| | Tanxin Liu |

| | |
|---|---|
| | Qizhi Yao |
| | Tao Liu |
| | Sarah Fahle |
| | Adam Butterworth |
| | Md Ashad Alam |
| | Jaydutt V. Vadgama |
| | Youping Deng |
| | Hong-Wen Deng |
| | Chong Wu |
| | Yong Wu |
| | Lang Wu |
| **Order of Authors Secondary Information:** | |

**Response to Reviewers:**

Re: GIGA-D-23-00321

Proteome-wide association study and functional validation identify novel protein markers for pancreatic ductal adenocarcinoma

Authors' responses to reviewers (Page and line numbers in our responses refer to the revised version of the manuscript with TRACK CHANGES)

Reviewer #1:

Proteome-Wide Association Study (PWAS) marks a significant advancement in biomedical research, bears great potential in identifying protein biomarkers linked to cancer's onset, progression, and treatment response, which are crucial for early detection, diagnosis, and monitoring. In the present study, Jingjing et al. leverage genome and plasma proteome data from 2,481 healthy individuals of European descent from the INTERVAL study to develop protein genetic prediction models. Their PWAS investigation, using these models, aims to identify potential protein markers for cancer. They notably pinpoint two novel proteomic markers, GOLM1 and B4GALT1, that may significantly influence pancreatic ductal adenocarcinoma cell behaviors.

In general, this pioneering PWAS work in exploring genetically predicted blood protein concentrations and their association with PDAC risk is undeniably a breakthrough in cancer research. However, the second part of this study, namely the process used to screen out GOLMA1 and B4GALT1 raised some questions and concerns.

Specifically In the words from 364 to line 367. The authors claimed that "Among the 16 novel associated proteins, analysis of TGCA data also revealed potential relevance of B4GT1 and GOLM1 with tumor development (data not shown). Consequently, these two proteins were selected as the targets for experimental validation to further investigate their potential roles in PDAC development." I don't understand why they addressed "data not shown". The absence of this crucial data and the rationale for prioritizing these two proteins over other 14 proteins are not clear. This omission is particularly concerning as neither B4GT1 nor GOLM1 is listed in Supplementary Table 2 as having relevant somatic mutations using TCGA data.

Response-1:
Thank you very much for your insightful comments and suggestions concerning our paper. We agree that these points are pivotal for understanding the unique significance of B4GT1 and GOLM1. Please allow us to provide further information to clarify these issues.

Regarding your point on "data not shown", to substantiate our selection of B4GT1 and GOLM1, we have now included the analysis result of TCGA data as supplementary figures (Supplementary Fig. 2 and 3). In brief, we have conducted a comprehensive

bioinformatic analysis leveraging data from TCGA, which clearly indicated the potential relevance of B4GALT1 and GOLM1 with pancreatic tumor development. We apologize for the omission in the previous version of the manuscript.

Page 12, Lines 274-286:
Gene Expression and Survival Analysis with TCGA Database
The examination of GOLM1 and B4GALT1 gene expressions in Pancreatic Adenocarcinoma (PAAD) was conducted using GEPIA (Gene Expression Profiling Interactive Analysis). The platform, accessible at the following web link: http://gepia.cancer-pku.cn/, facilitated analysis with a dataset consisting of 179 tumor samples and 171 normal controls. The focus of survival analysis was exclusively on PAAD, leveraging TCGA data through the GEPIA web server.
Customized gene selection, normalization, and survival methodologies were implemented to suit the unique characteristics of PAAD. Cohort thresholds were defined, restricting dataset selection to PAAD, and survival plots were generated. These measures were designed to precisely identify the correlation between gene expression and survival outcomes specific to this type of cancer.

Page 18, Lines 423-439:
Among the 16 novel associated proteins, analysis of TGCA data also revealed potential relevance of B4GT1 and GOLM1 with tumor development (Supplementary Figure 2 and 3). The examination of GOLM1 and B4GALT1 gene expression in PADD cancer was conducted using GEPIA (Gene Expression Profiling Interactive Analysis). The analysis involved a dataset consisting of 179 tumor samples and 171 normal controls. The box plot analysis revealed a statistically significant increase in GOLM1 (Supplementary Figure 2A) and B4GALT1 (Supplementary Figure 3A) expression in the tumor samples as compared with the normal control group. GEPIA, accessible through the following web link: http://gepia.cancer-pku.cn/, served as the platform for this investigation. The survival analysis of GOLM1 and B4GALT1 gene expression in PADD cancer was conducted using GEPIA. Survival plots revealed a significant decrease in overall survival (OS) and disease-free survival (DFS) among tumor samples exhibiting elevated GOLM1 or B4GALT1 expression (n=89) compared with those with low expression (n=89). Employing the Log-rank test for hypothesis testing, our findings emphasize a noteworthy correlation between heightened gene expression and reduced OS and DFS in the PADD cancer cohort (Supplementary Figure 2B, C, Supplementary Figure 3B, C).

I could understand that due to the novelty of PWAS, the authors are able to successfully identified B4GT1 and GOLM1 as important markers at proteomic level. However, through literature search, there is very limited published peer-reviewed papers to show them play any roles in Pancreatic ductal adenocarcinoma in other omics level, like genetics, genomics, transcriptomics.
Response-2:
Thanks for your comment. Your statement underlines a relevant point about the yet unclear roles of B4GT1 and GOLM1 at other omics levels in pancreatic ductal adenocarcinoma. We think that this indeed underscores the potential of our innovative PWAS design in uncovering novel proteins that could not have been identified if we use another design focusing on other omics level. As described above in another response, after we identified these two proteins, when we focused on their RNA expression levels, we could identify additional evidence at RNA levels showing their potential relevance with PDAC.

Were the other 14 proteins subjected to similar experimental protocols, and if so, what were the findings? This information is vital for understanding the unique significance of B4GT1 and GOLM1 in this context.
Response-3:
Thanks for your comment. We conducted a bioinformatics analysis using the GEPIA online TCGA tool to investigate the survival rates associated with the expression of the 16 genes encoding the novel proteins with genetically predicted concentrations in plasma linked to PDAC risk. The findings indicate that, in pancreatic adenocarcinoma (PAAD), GOLM1, B4GALT1, FAM20B, FAB3D, and LRPAP1 exhibit significantly higher expression in tumor tissues, and they are associated with noteworthy survival

rate differences among patients. Further validation through mRNA PCR tests in normal Human Pancreatic Duct Epithelial Cell Line and pancreatic cancer cell lines (PANC-1, SU.86.86) revealed that only GOLM1 and B4GALT1 displayed elevated expression in pancreatic cancer cell lines. Consequently, for subsequent biological investigations, GOLM1 and B4GALT1 were selected due to their distinct high expression in pancreatic cancer cell lines, suggesting their potential relevance to the pathogenesis of pancreatic cancer.

Experimental studies to validate the role of all 16 novel proteins would be exhaustive in terms of resources and time. Given the supportive associations of B4GALT1 and GOLM1 revealed by the TCGA data, it was prudent to prioritize these two for experimental validation, in the current stage of study. We believe this maybe the most efficient strategy to follow up on a large number of candidates generated from a high-throughput PWAS, but agree that the other 14 proteins certainly warrant further investigation.

Finally, concerning the other 14 proteins, although they were not subjected to the same experimental protocols, ongoing studies in our lab are focused on further analyzing these proteins in vitro and in vivo to better understand their roles in PDAC. As these studies were not included in the current manuscript, we would be delighted to share our findings in an appropriate future publication.

We hope these explanations address your concerns, and we thank you again for improving the quality of our work through your insightful comments.

Reviewer #2:

Zhu et al. constructed a series of pQTL models and used them to identify genetic predicted serum protein markers for pancreatic ductal adenocarcinoma, followed by a series of functional validations, which may provide valuable clues for prediction and treatment of PDAC. I have several concerns on this study.

Major concerns:
1. This study integrated both cis- and trans-acting elements to construct pQTL models. It would be better to provide the heritability of each pQTL model constructed and the comparison results (such as the h2 explained and predictive performance on gene expression) with those focus solely on cis-acting variants, as the author stated that the integration strategy has an enhanced statistical power.

Rsponse-1:
Thank you very much for your insightful comments. We have compared h2 of the prediction models between those with cis+trans factors and only cis genetic factors. The results indeed showed that when involving trans-acting elements, enhanced statistical power could be achieved.

Page 8, Lines 181-185:
We also estimated the genetic heritability of plasma proteins (the proportion of the variation of protein levels that could be explained by potential predictors) using GCTA1. We compared the heritability of plasma proteins when using cis+trans SNPs vs only cis SNPs to assess whether it could capture more heritability when involving trans-SNPs.

Page 16, Lines 376-383:
We compared the heritability of the prediction models established using cis+trans and vs cis-only predictors strategies. Here, we focused on the 490 models established using both cis and trans SNPs in the main analysis. The results showed that 250 out of the 490 (51.02%) models have higher estimated heritability with the cis+trans strategy (Supplementary Table 2), and 215 proteins (43.88%) showed the same estimated heritability between cis+trans and cis-only strategies (Supplementary Table 2). Only 25 proteins (5.10%) showed lower estimated heritability when using cis+trans strategy (Supplementary Table 2). These results showed that trans SNPs could in general increase heritability of the prediction models.

2. The integration strategy is somewhat like some PGS methods (such as C+T). Would the author consider to try some other strategies used in common PGS analysis? For example, using LD clumping for SNPs selection, trying some other P value threshold combinations to define and select gene- associated SNPs in cis and trans regions, and using the bslmm strategy, which seems to be demonstrated to have decent performance in the FUSION article.
Rsponse-2:
We thank the reviewer for the comments. We have now performed several additional robustness analyses, including using the bslmm method, LD clumping for SNP selection, and different p-value thresholds. The results show that our results are robust under different methods/thresholds.

Page 10, Lines 220-233:
Robustness analyses
To further examine whether the identified significant associations from the main analyses may be robust to different strategies, three alternative strategies were used to test these proteins under different scenorios. Firstly, we established prediction models using the bslmm method embedded in TWAS/FUSION software. This method was not enabled by the default parameter due to the intensive Markov chain Monte Carlo (MCMC) computation, although bslmm has some advantages and might increase prediction accuracy in some conditions. Secondly, we pruned the highly correlated SNPs and only SNPs that are weakly correlated with each other were used as potential predictors. In the current analysis, we pruned SNPs using pruning parameters r2 = 0.1 and distance = 250 kb. Thirdly, we assessed the robustness of the significant association results by examining different p-value cutoffs for selecting informative trans-regions (p-value < 5×10-7, p-value < 5×10-9, and p-value < 5×10-10) as candidate predictors for model building. The association results with a nominal p-value < 0.05 and consistent effect direction were considered to be replicated.

Page 16, Lines 384-393:
The robustness analysis showed that all the 40 significantly PDAC-associated proteins had the same effect directions (Supplementary Table 3). A total of 39 proteins could be tested using the bslmm method and 37 out of 39 (94.87%) could be replicated (except for SEMA6A and CHST11 proteins). When we removed highly correlated SNPs and only weak correlated SNPs were used for establishing prediction models, a total of 39 prediction models were established. The association results showed that associations of 38 out of the 39 (97.44%) proteins could be replicated (Supplementary Table 3). In addition, three different p-value thresholds (p-value < 5×10-7, p-value < 5×10-9, and p-value < 5×10-10) for selecting trans-SNPs were examined (Supplementary Table 3). All the association results were consistent with those in our main analysis. The above results showed the robustness of our main results.

3. This study selected proteins for pWAS analysis based on prediction R/R2 of pQTL models. Would the author take the h2 of each pQTL model into consideration as the FUSION article did?
Rsponse-3:
We thank the reviewer for the comments. The R2≥0.01was a common threshold used in previous relevant omics integration studies. Here we also added the information of h2 estimated using the GCTA software in the revised manuscript (main text as well as Tables 1 and 2) 1.

Page 8, Lines 174-175:
R2≥0.01 was used as the threshold for selecting satisfactory prediction models, which is commonly used in relevant omics integration studies.
Page 15, Lines 361-362:
The heritability of the proteins ranged from 0.001 to 0.87, with an average value of 0.14.

4. Although the author used the TWAS/FUSION framework for pQTL models construction and protein-PDAC association assessment, it would be better to add more description into the supplementary file on how this framework was applied to the

| | |
|---|---|
| | current study.<br>Rsponse-4:<br>We thank the reviewer for the comments. We have now added more descriptions of the way we performed the association assessment.<br><br>Page 9, Lines 212-216:<br>We calculated the PWAS test statistic Z-score = w'Z/(w'Σs,sw)1/2, where the Z is a vector of standardized effect sizes of SNPs for a given protein (Wald z-scores), w is a vector of prediction weights for the abundance feature of the protein being tested, and the Σs,s is the LD matrix of the SNPs estimated from the 1000 Genomes Project as the LD reference panel.<br><br>Reference<br>1.Yang, J., Lee, S. H., Goddard, M. E. & Visscher, P. M. GCTA: a tool for genome-wide complex trait analysis. Am J Hum Genet 88, 76–82 (2011). |
| **Additional Information:** | |
| **Question** | **Response** |
| Are you submitting this manuscript to a special series or article collection? | No |
| **Experimental design and statistics**<br><br>Full details of the experimental design and statistical methods used should be given in the Methods section, as detailed in our Minimum Standards Reporting Checklist. Information essential to interpreting the data presented should be made available in the figure legends.<br><br>Have you included all the information requested in your manuscript? | Yes |
| **Resources**<br><br>A description of all resources used, including antibodies, cell lines, animals and software tools, with enough information to allow them to be uniquely identified, should be included in the Methods section. Authors are strongly encouraged to cite Research Resource Identifiers (RRIDs) for antibodies, model organisms and tools, where possible.<br><br>Have you included the information requested as detailed in our Minimum Standards Reporting Checklist? | Yes |

| | |
|---|---|
| **Availability of data and materials**<br><br>All datasets and code on which the conclusions of the paper rely must be either included in your submission or deposited in publicly available repositories (where available and ethically appropriate), referencing such data using a unique identifier in the references and in the "Availability of Data and Materials" section of your manuscript.<br><br>Have you have met the above requirement as detailed in our Minimum Standards Reporting Checklist? | Yes |

Manuscript Click here to access/download;Manuscript;PWAS R1 with DOI.docx



# Proteome-wide association study and functional validation identify novel protein markers for pancreatic ductal adenocarcinoma

Jingjing Zhu[1*], Ke Wu[2*], Shuai Liu[3*], Alexandra Masca[3], Hua Zhong[3], Tai Yang[4], Dalia H Ghoneim[3], Praveen Surendran[5], Tanxin Liu[6], Qizhi Yao[7,8], Tao Liu[9], Sarah Fahle[5], Adam Butterworth[5,10], Md Ashad Alam[11], Jaydutt V. Vadgama[2], Youping Deng[1], Hong-Wen Deng[11], Chong Wu[12#], Yong Wu[2#], Lang Wu[3#]

1. Department of Quantitative Health Sciences, John A. Burns School of Medicine, University of Hawai‘i at Mānoa, Honolulu, HI, USA

2. Division of Cancer Research and Training, Department of Internal Medicine, Charles R. Drew University of Medicine and Science, David Geffen UCLA School of Medicine and UCLA Jonsson Comprehensive Cancer Center, Los Angeles, CA 90095, USA

3. Cancer Epidemiology Division, Population Sciences in the Pacific Program, University of Hawai‘i Cancer Center, University of Hawai‘i at Mānoa, Honolulu, HI, USA

4. Department of Biostatistics, University of Michigan - Ann Arbor, MI, USA

5. MRC/BHF Cardiovascular Epidemiology Unit, Department of Public Health and Primary Care, University of Cambridge, Cambridge, UK

6. Department of Epidemiology, Johns Hopkins Bloomberg School of Public Health, Baltimore, MD, USA

7. Division of Surgical Oncology, Michael E. DeBakey Department of Surgery, Baylor College of Medicine, Houston, Texas

8. Center for Translational Research on Inflammatory Diseases (CTRID), Michael E. DeBakey VA Medical Center, Houston, Texas

9. Biological Sciences Division, Pacific Northwest National Laboratory, Richland, WA 99354, USA

10. NIHR Blood and Transplant Research Unit in Donor Health and Genomics, Department of Public Health and Primary Care, University of Cambridge, Cambridge, UK

11. Tulane Center for Biomedical Informatics and Genomics, Division of Biomedical Informatics and Genomics, Deming Department of Medicine, Tulane University, 1440 Canal Street, New Orleans, 70112, LA, USA

12. Department of Biostatistics, The University of Texas MD Anderson Cancer Center, Houston, TX, USA

[*] these authors contributed equally to this work and are co-first authors

[#] these authors jointly supervised this work and are co-senior authors

**Running title:** Predicted protein biomarkers for pancreatic cancer

**Corresponding to:** Lang Wu, Cancer Epidemiology Division, Population Sciences in the Pacific Program, University of Hawai‘i Cancer Center, University of Hawai‘i at Mānoa, Honolulu, HI, 96813, USA. Email: lwu@cc.hawaii.edu. Phone: (808)564-5965; or Yong Wu, Department of Internal Medicine, Charles Drew University of Medicine and Science, Los Angeles, CA 90059, USA. Email: yongwu@cdrewu.edu; or Chong Wu, Department of Biostatistics, The University of Texas MD Anderson Cancer Center, Houston, TX, USA. Email: cwu18@mdanderson.org

**Abstract**

Pancreatic ductal adenocarcinoma (PDAC) remains a lethal malignancy, largely due to the paucity of reliable biomarkers for early detection and therapeutic targeting. Existing blood protein biomarkers for PDAC often suffer from replicability issues, arising from inherent limitations such as unmeasured confounding factors in conventional epidemiologic study designs. To circumvent these limitations, we use genetic instruments to identify proteins with genetically predicted levels to be associated with PDAC risk. Leveraging genome and plasma proteome data from the INTERVAL study, we established and validated models to predict protein levels using genetic variants. By examining 8,275 PDAC cases and 6,723 controls, we identified 40 associated proteins, of which 16 are novel. Functionally validating these candidates by focusing on two selected novel protein-encoding genes, *GOLM1* and *B4GALT1*, we demonstrated their pivotal roles in driving PDAC cell proliferation, migration, and invasion. Furthermore, we also identified potential drug repurposing opportunities for treating PDAC.

**Significance:**

PDAC is a notoriously difficult-to-treat malignancy, and our limited understanding of causal protein markers hampers progress in developing effective early detection strategies and treatments. Our study identifies novel causal proteins using genetic instruments and subsequently functionally validates selected novel proteins. This dual approach enhances our understanding of PDAC etiology and potentially opens new avenues for therapeutic interventions.

**Keywords:** Biomarkers, protein, genetics, pancreatic cancer, risk

## Introduction

Pancreatic cancer is the seventh leading cause of cancer deaths in industrialized countries with pancreatic ductal adenocarcinoma (PDAC) making up over 90% of pancreatic cancer cases (1). According to GLOBOCAN 2020 cancer statistics, pancreatic cancer is the 14th most common cancer type with 495,773 new cases in 2020. There are almost the same number of deaths caused by pancreatic cancer (466,003 deaths) in 2020, accounting for 4.7% of all cancer related deaths (2). Owing to its often asymptomatic or non-specific symptoms during early stages, a majority of patients are usually diagnosed in advanced stages. This results in 80-90% of pancreatic tumors being unresectable upon diagnosis, leading to a dismal prognosis: a mere 9% five-year survival rate after diagnosis (1). Given these dire statistics, there is an urgent need to identify effective biomarkers for screening or early detection in high-risk populations. Equally crucial is the development of improved therapeutic strategies to improve PDAC outcome.

Currently, serum cancer antigen (CA) 19-9 is the only diagnostic biomarker for pancreatic cancer approved by the U.S. FDA. However, elevated levels of CA 19-9 are related to other conditions, and its performance as a diagnostic tool for pancreatic cancer is far from ideal (3): it has a poor positive predictive value (0.5-0.9%), along with restricted specificity (82-90%) and sensitivity (79-81%). Previous studies have also reported several other circulating blood protein biomarkers that are potentially associated with pancreatic cancer risk, such as CA242, PIVKA-II, and PAM4 (4-7). However, results from existing studies often involving small sample sizes and findings are inconsistent. It is well known that the conventional epidemiologic study design measuring levels of proteins directly may be subject to selection bias and residual or

unmeasured confounding, which could also contribute to the inconsistent findings in the existing literature.

An alternative design of using genetic instruments may decrease many limitations of existing studies, due to the nature of random assortment of alleles from parents to offspring during gamete formation (8,9). Inspired by transcriptome-wide association study (TWAS), one may build comprehensive genetic prediction models for each protein to capture the prediction value of multiple single nucleotide polymorphisms (SNPs). Unlike conventional TWAS type of methods, which typically focus solely on cis-acting variants, our study enhanced statistical power by integrating both cis- and trans-acting elements into our genetic prediction models. Furthermore, as TWAS or PWAS results imply causality under stringent valid instrumental variable assumptions, we further functionally validated two novel proteins.

In the current study, we applied such a study design to identify novel proteins associated with PDAC risk. To our knowledge, this is the first large-scale proteome wide association study (PWAS) using comprehensive protein genetic prediction models as instruments to assess the associations between genetically predicted blood concentrations of proteins and PDAC risk. We used data for 8,275 cases and 6,723 controls of European descent from the Pancreatic Cancer Cohort Consortium (PanScan) and the Pancreatic Cancer Case-Control Consortium (PanC4). Beyond identifying novel proteins, we functionally validated two of them. Moreover, we generated a list of drugs targeting the identified proteins which may serve as candidates for drug repurposing of PDAC.

## Methods

### *Protein genetic prediction model development and validation*

We leveraged the genome and plasma proteome data of healthy European subjects included in the INTERVAL study to establish (subcohort1) and validate (subcohort2) protein genetic prediction models. The details of the INTERVAL study data have been published previously (10-14). Briefly, participants were generally healthy. The SOMAscan assay was used to collect the relative levels of 3,620 plasma proteins or complexes. Quality control (QC) was performed at both the sample and SOMAmer level. Approximately ~830,000 genetic variants were measured on the Affymetrix Axiom UK Biobank genotyping array. Standard sample and variant QC were conducted. SNPs were phased using SHAPEIT3 and imputed using a combined 1000 Genomes Phase 3-UK10K reference panel, which resulted in over 87 million imputed variants. The SNPs were further filtered using criteria of 1) imputation quality of at least 0.7, 2) minor allele count of at least 5%, 3) Hardy Weinberg Equilibrium (HWE) $p \geq 5\times10^{-6}$, (4) missing rates < 5%, and (5) presenting in the 1000 Genome Project data for European populations. Overall there were 4,662,360 variants passing these criteria.

In subcohort 1 (N=2,481), as described elsewhere (10), protein concentrations were log transformed and adjusted for age, sex, duration between blood draw and processing, and the top three principal components. For the rank-inverse normalized residuals of each protein, we followed the TWAS/FUSION framework to establish prediction models, using nearby variants (within 100kb) of potentially associated SNPs as candidate predictors (15). A false discovery rate (FDR) < 0.05 was used to determine potentially associated SNPs in cis regions (within 1 Mb of the transcriptional start site (TSS) of the gene encoding the target protein of interest) and *P*-value $\leq 5\times10^{-8}$ was used to determine potentially associated SNPs in trans regions. We only included strand unambiguous SNPs. Four methods of best linear unbiased predictor (blup), elastic net, LASSO, and top1 were used to develop the models. For each protein of interest, the model

showing the most significant cross-validation *P*-value among those developed using the four methods was selected. $R^2 \geq 0.01$ was used as the threshold for selecting satisfactory prediction models, which is commonly used in relevant omics integration studies (16-30). For protein prediction models with $R^2 \geq 0.01$, external validation was conducted using genetic and protein data of subcohort 2 (N=820). Briefly, predicted protein expression levels were estimated by applying the developed protein prediction models to the genetic data, which were further compared with the measured levels for each protein of interest. Proteins with a model prediction $R^2$ of $\geq 0.01$ in subcohort1 and a correlation coefficient of $\geq 0.1$ in subcohort2 were selected for association analysis with PDAC risk. We also estimated the genetic heritability of plasma proteins (the proportion of the variation of protein levels that could be explained by potential predictors) using GCTA (31). We compared the heritability of plasma proteins when using *cis*+*trans* SNPs vs only *cis* SNPs to assess whether it could capture more heritability when involving *trans*-SNPs.

***Examine associations of genetically predicted protein levels with PDAC risk***

To investigate the associations between genetically predicted circulating protein levels and PDAC risk, the validated protein genetic prediction models were applied to the summary statistics from a large GWAS of PDAC risk. In the present work, we used data from GWAS conducted in the PanScan and PanC4 consortia downloaded from the database of Genotypes and Phenotypes (dbGaP), including 8,275 PDAC cases and 6,723 controls of European ancestry. Detailed information on this dataset has been included elsewhere (17,20,32). Briefly, four GWAS studies, namely, PanScan I, PanScan II, PanScan III, and PanC4, were genotyped using the Illumina HumanHap550, 610-Quad, OmniExpress, and OmniExpressExome arrays,

respectively. Standard QC procedures were performed according to the consortia guidelines (32). Study participants who were related to each other, had sex discordance, had genetic ancestry other than Europeans, had a low call rate (less than 98% and 94% in PanC4 and PanScan, respectively), or had missing information on age or sex were excluded. Duplicated SNPs, and those with a high missing call rate (at least 2% and 6% in PanC4 and PanScan, respectively) or with violations of Hardy-Weinberg equilibrium (HWE) ($P < 1\times10^{-4}$ and $P < 1\times10^{-7}$ in PanC4 and PanScan, respectively), were also removed. Regarding SNP data from PanC4, those with minor allele frequency < 0.005, with more than two discordant calls in duplicate samples, with more than one Mendelian error in HapMap control trios, and those with sex difference in allele frequency > 0.2 or in heterozygosity > 0.3 for autosomes/XY in European descendants were further removed. We performed genotype imputation using Minimac3 after prephasing with SHAPEIT from a reference panel of the Haplotype Reference Consortium (r1.1 2016) (33,34). We retained imputed SNPs with an imputation quality of ≥0.3. The associations between individual genetic variants and PDAC risk were further estimated adjusting for age, sex and top principal components. The TWAS/FUSION framework was used to assess the protein-PDAC risk associations, by leveraging correlations between variants included in the prediction models based on the phase 3, 1000 Genomes Project data for European populations (15). We calculated the PWAS test statistic $Z$-score = $w'Z/(w'\Sigma_{s,s}w)^{1/2}$, where the $Z$ is a vector of standardized effect sizes of SNPs for a given protein (Wald $z$-scores), $w$ is a vector of prediction weights for the abundance feature of the protein being tested, and the $\Sigma_{s,s}$ is the LD matrix of the SNPs estimated from the 1000 Genomes Project as the LD reference panel. We used the false discovery rate (FDR) corrected $P$-value threshold of ≤ 0.05 to determine significant associations between genetically predicted protein concentrations and risk of PDAC.

***Robustness analyses***

To further examine whether the identified significant associations from the main analyses may be robust to different strategies, three alternative strategies were used to test these proteins under different scenarios. Firstly, we established prediction models using the bslmm method embedded in TWAS/FUSION software. This method was not enabled by the default parameter due to the intensive Markov chain Monte Carlo (MCMC) computation, although bslmm has some advantages and might increase prediction accuracy in some conditions. Secondly, we pruned the highly correlated SNPs and only SNPs that are weakly correlated with each other were used as potential predictors. In the current analysis, we pruned SNPs using pruning parameters $r^2 = 0.1$ and distance = 250 kb. Thirdly, we assessed the robustness of the significant association results by examining different *p*-value cutoffs for selecting informative *trans*-regions (*p*-value $< 5\times10^{-7}$, *p*-value $< 5\times10^{-9}$, and *p*-value $< 5\times10^{-10}$) as candidate predictors for model building. The association results with a nominal *p*-value < 0.05 and consistent effect direction were considered to be replicated.

***Somatic variants of genes encoding associated proteins***

For each of the genes encoding the proteins that are identified to be associated with PDAC risk, we evaluated potentially deleterious somatic level mutations in 150 PDAC patients included in The Cancer Genome Atlas (TCGA). The potentially deleterious somatic variants include missense mutations, splice site mutations, nonstop mutations, nonsense mutations, frameshift mutations, in-frame mutations and translation start site mutations.

The somatic level genetic changes were called using MuTect2 (doi: https://doi.org/10.1101/861054) and deposited to the TCGA data portal. The enrichment of proportion of assessed genes containing such somatic level genetic events compared with the proportion of all protein-coding genes across the genome was evaluated using socscistatistics online website (https://www.socscistatistics.com/tests/ztest/default2.aspx).

***Ingenuity Pathway Analysis (IPA) and Protein-Protein Interaction (PPI) analysis***

To further assess whether genes encoding the identified PDAC associated proteins are enriched in specific pathways, molecular and cellular functions, and networks, we performed the enrichment analysis using Ingenuity Pathway Analysis (IPA) software (35). The "enrichment" score (Fisher exact test *P* value) that measures overlap of observed and predicted regulated gene sets was generated for each of the tested gene sets. The most significant pathways and functions with an enrichment *P* value less than 0.05 were reported. We also built protein-protein interaction (PPI) network using STRING database version 11.5 (https://string-db.org/) with 0.400 confidence level (36). The STRING database integrates different curated databases containing information on known and predicted functional protein–protein associations.

***Drug repurposing analysis***

For the identified proteins, we further assessed whether there is any evidence supporting their potential roles in PDAC by using the OpenTargets (37). Focusing on those showing a potential relevance, we further mined evidence of their targeting drugs using the DrugBank (38) database. We also conducted molecular docking analysis for the identified proteins and corresponding candidate drug agents (39). Specifically, we downloaded the 3D structure of targeted proteins from Protein Data Bank (PDB) (40) with source code 1CPB, 3CDZ, 1IGR,

3DFK, 5NO06, and drug agents from the PubChem database (41). We further worked out molecular docking between each of the proteins and the corresponding meta-drug agents to calculate the binding affinity scores (kcal/mol) for each pair of proteins and drugs.

***In vitro functional validation of genes encoding selected associated novel proteins***

**Cell Lines and Culture Condition**

Human pancreatic cancer cell lines PANC-1 and SU.86.86 were obtained from ATCC (American Type Culture Collection). All cells were cultured in vitro in DMEM (Dulbecco's modified eagle medium) high glucose medium (Gibco, Novato, CA, United States) supplemented with 10% (v/v) fetal bovine serum (FBS) (Gibco). Cells were incubated at 37°C with 5% CO2.

**Gene Expression and Survival Analysis with TCGA Database**

The examination of *GOLM1* and *B4GALT1* gene expressions in Pancreatic Adenocarcinoma (PAAD) was conducted using GEPIA (Gene Expression Profiling Interactive Analysis). The platform, accessible at the following web link: http://gepia.cancer-pku.cn/, facilitated analysis with a dataset consisting of 179 tumor samples and 171 normal controls. The focus of survival analysis was exclusively on PAAD, leveraging TCGA data through the GEPIA web server.

Customized gene selection, normalization, and survival methodologies were implemented to suit the unique characteristics of PAAD. Cohort thresholds were defined, restricting dataset selection to PAAD, and survival plots were generated. These measures were designed to precisely identify the correlation between gene expression and survival outcomes specific to this type of cancer.

**Western blotting**

Post 72-hour silencing, we processed control, B4GALT1-silenced, and GOLM1-silenced cells for Western blotting. Cells were lysed using RIPA buffer, and equal protein amounts were separated on 10% or 12% SDS polyacrylamide gels, then transferred onto PVDF membranes. To prevent non-specific antibody binding, membranes were blocked with 5% milk in TBS with 0.1% Tween for an hour. They were then probed with anti-B4GALT1, anti-GOLM1, and anti-GAPDH antibodies, followed by their respective HRP-conjugated secondary antibodies. Signal detection was performed using Pierce™ ECL Western Blotting Substrate and images were captured and analyzed using Odyssey FC and ImageStudio Software.

**Quantitative Real-Time PCR (qPCR)**

Total RNA was extracted from cells using TRNzol reagent according to the manufacturer's protocol. The concentration of RNA was determined using a UV spectrophotometer. Subsequently, 2 mg of total RNA was reverse transcribed into cDNA using the iScript™ cDNA Synthesis Kit. qPCR analysis was performed on the CFX96™ Real-Time PCR Detection System using the iTaq™ Universal SYBR® Green Supermix. The aim was to detect the expression levels of three genes: B4GALT1, GOLM1, and GAPDH mRNAs. Specific primer pairs were used for each gene. For B4GALT1, the forward sequence was GTATTTTGGAGGTGTCTCTGCTC and the reverse sequence was GGGCGAGATATAGACATGCCTC. For GOLM1, the forward sequence was ATCACCACAGGTGAGAGGCTCA and the reverse sequence was ACTTCCTCTCCAGGTTGGTCTG. For the housekeeping gene GAPDH, the forward sequence was GTCTCCTCTGACTTCAACAGCG and the reverse sequence was ACCACCCTGTTGCTGTAGCCAA. During the qPCR analysis, melting curves were generated

to detect primer-dimer formation and confirm the specificity of the gene-specific peaks for each target. To ensure accurate quantification, the expression data were normalized to the amount of GAPDH mRNA expressed.

**Transfection of siRNA**

The transfection of small-interfering RNA (siRNA) was performed using specific human siRNAs targeting GOLM1 (SASI_Hs01_00223155), B4GALT1 (SASI_Hs01_00080445), and the MISSION siRNA universal negative control, all of which were obtained from Sigma-Aldrich (St. Louis, MO). Cells were seeded in 6-well plates at a density of 1.5x105 cells per well and subsequently transfected with the siRNAs at a concentration of 40 nM. The transfection procedure utilized the lipofectamine 2000 reagent (Invitrogen, Carlsbad, CA, United States) following the manufacturer's recommended guidelines. Gene silencing at both mRNA and protein levels was typically observed 72 h post-transfection. As such, the cells were collected and subjected to assays at the 72-hour time point to assess the efficacy of gene silencing.

**Cell Proliferation Assay**

To observe cell proliferation, cells were transfected with Mock siRNA, siGOLM11 and siB4GAL1 (40 nM). At 24 h after transfection, the cells were trypsinized and seeded into 96-well plates (Corning, NY, United States) at a density of 5000 cells/well in 200 ul media. The plates were incubated in a 37°C humidified incubator. On each day for [3-(4,5-dimethylthiazol-2-yl)-5-(3-carboxymethoxyphenyl)-2-(4-sulfophenyl)-2H-tetrazolium] (MTS) assay.

***In vitro* invasion assay**

Cell invasion was assessed following transfection with Mock siRNA, siGOLM11, and siB4GAL1 (40 nM). A modified Boyden chamber method was employed. Matrigel (BD Biosciences) was coated on the upper chamber of Transwell inserts (Corning, 8 µm pore size) at a concentration of 300 µg/ml, allowing gel formation for 2 hours at 37°C. Cells (5 x 10^4) were then suspended in 200 µl of serum-free medium and added to the upper chamber. The lower chamber contained 600 µl of medium with 10% FBS, acting as a chemoattractant. Following 24 hours of incubation at 37°C, non-invading cells on the upper membrane surface were gently removed using a cotton swab. Cells that invaded the lower membrane surface were fixed with 4% paraformaldehyde and stained with 0.1% crystal violet. Invasion was quantified by counting the stained cells on the underside of the membrane using a light microscope (10 random fields at 200x magnification). All experiments were performed in triplicate to ensure robustness of the findings.

**Wound Scratch assay**

After 24 hours of transfection with Mock siRNA, siGOLM11, and siB4GAL1, PANC-1 and SU.86.86 cells were cultured in a 96-well plate to form a monolayer. Using BioTek's AutoScratchTM Wound Making Tool, straight scratches were carefully created on the cell monolayer to mimic wounds, following the equipment manual's instructions. Time-lapse images of the scratches were captured at specific intervals (e.g., 0 hours, 12 hours, 24 hours, etc.) using the CytationTM 5 Cell Imaging Multi-Mode Reader. Subsequently, image analysis software was employed to quantify the closure of the wounds at each time point. Statistical analysis was performed to compare the wound closure rates at different time points, and the results were presented graphically.

**Results**

The overall workflow of this study is shown in **Figure 1**. Of the proteins assessed, we were able to develop prediction models for 1,864 proteins with a prediction performance $R^2 \geq 0.01$. In the external validation step, 1,389 of them further demonstrated a correlation coefficient of ≥ 0.1 for predicted expression and measured expression levels. The heritability of the proteins ranged from 0.001 to 0.87, with an average value of 0.14. Of such proteins, we observed significant associations between genetically predicted expression levels of 40 proteins and PDAC risk at a false discovery rate (FDR) *p*-value of ≤ 0.05 (**Figure 2, Tables 1 and 2**). Of the associated proteins, 16 are novel ones that have not been reported in previous studies (**Table 1**). Positive associations were observed for 10 of these proteins, and inverse associations were observed for six proteins (**Table 1**). The other 24 associated proteins have been previously reported in our study using pQTL as instruments (42) (**Table 2**). These include 10 that demonstrated positive associations and 14 that showed inverse associations.

For the other proteins that were reported in our previous study using pQTL as instruments (42), while did not show a significant association after FDR correction in the current study (**Supplementary Table 1**), except for sTie-2, the directions of effect were consistent in the current study compared with those in the published work. Among them, for eight proteins, their associations were at $P<0.05$ in the current work using protein genetic prediction models as instruments (**Supplementary Table 1**).

We compared the heritability of the prediction models established using *cis*+*trans* and vs *cis*-only predictors strategies. Here, we focused on the 490 models established using both *cis* and *trans* SNPs in the main analysis. The results showed that 250 out of the 490 (51.02%) models have higher estimated heritability with the *cis*+*trans* strategy (**Supplementary Table 2**), and 215

proteins (43.88%) showed the same estimated heritability between *cis*+*trans* and *cis*-only strategies (**Supplementary Table 2**). Only 25 proteins (5.10%) showed lower estimated heritability when using the *cis*+*trans* strategy (**Supplementary Table 2**). These results showed that *trans* SNPs could in general increase heritability of the prediction models.

The robustness analysis showed that all the 40 PDAC-associated proteins had the same effect directions (**Supplementary Table 3**). A total of 39 proteins could be tested using the bslmm method and 37 out of the 39 (94.87%) could be replicated (except for SEMA6A and CHST11 proteins). When we removed highly correlated SNPs and only weak correlated SNPs were used for establishing prediction models, a total of 39 prediction models were established. The association results showed that associations of 38 out of the 39 (97.44%) proteins could be replicated (**Supplementary Table 3**). In addition, three different *p*-value thresholds (*p*-value < $5\times10^{-7}$, *p*-value < $5\times10^{-9}$, and *p*-value < $5\times10^{-10}$) for selecting trans-SNPs were examined (**Supplementary Table 3**). All the association results were consistent with those in our main analysis. The above results showed the robustness of our main results.

Based on a comparison of exome-sequencing data of tumor tissue and tumor-adjacent normal tissue obtained from 150 TCGA PDAC patients, the somatic level changes of potentially functional variants/mutations were observed in at least one patient for 10 of the 39 genes encoding identified associated proteins (**Supplementary Table 4**). This proportion (10/39=25.64%) is significantly higher (enrichment *P* value < 0.00001) than the overall observed proportion of potentially functional changes across the genes encoding the proteins tested for association analyses (95/1,218 = 7.80%; here 1,218 represents the number of the genes available in TCGA analysis as part of the genes encoding the 1,389 assessed proteins).

According to the IPA analysis, several cancer-related functions were enriched for the genes encoding our identified proteins (**Supplementary Table 5**). The top canonical pathways identified included IL-15 production ($P$=2.21×$10^{-3}$), Heparan Sulfate Biosynthesis (Late Stages) ($P$=2.97×$10^{-3}$), Heparan Sulfate Biosynthesis ($P$=3.99×$10^{-3}$), Sperm Motility ($P$=7.73×$10^{-3}$), and Dermatan Sulfate Biosynthesis (Late Stages) ($P$=0.01) (**Figure 3**). Among the related networks, the top network was cell-to-cell signaling and interaction, cardiovascular system development and function, organismal development (**Supplementary Figure 1**), followed by cancer, organismal injury and abnormalities, respiratory disease, free radical scavenging, cell death and survival, organismal injury and abnormalities, carbohydrate metabolism, small molecule biochemistry, cell cycle, and cancer, cell-to-cell signaling and interaction, cellular assembly and organization. Interactions among identified proteins were investigated based on STRING database (**Figure 3**). In the network, KDR was predicted to interact with IGF1R, NOTCH1, MET, SEMA6A, ENG, SELP, and SELE.

Based on interrogation using the OpenTargets and DrugBank database, ten of the identified proteins are supported to be relevant to PDAC (overall score >0 in OpenTargets) and are targets of existing drugs approved to be used to treat human conditions (**Table 3**). Our work indicates potential drug repurposing opportunities of these drug targets to other indications. The scores of molecular docking between each of the proteins and the corresponding meta-drug agents were included in **Table 3**.

Among the 16 novel associated proteins, analysis of TGCA data also revealed potential relevance of B4GT1 and GOLM1 with tumor development (**Supplementary Figure 2 and 3**). The examination of *GOLM1* and *B4GALT1* gene expression in PADD cancer was conducted using GEPIA (Gene Expression Profiling Interactive Analysis). The analysis involved a dataset

consisting of 179 tumor samples and 171 normal controls. The box plot analysis revealed a statistically significant increase in *GOLM1* (**Supplementary Figure 2A**) and *B4GALT1* (**Supplementary Figure 3A**) expression in the tumor samples as compared with the normal control group. GEPIA, accessible through the following web link: http://gepia.cancer-pku.cn/, served as the platform for this investigation. The survival analysis of *GOLM1* and *B4GALT1* gene expression in PADD cancer was conducted using GEPIA. Survival plots revealed a significant decrease in overall survival (OS) and disease-free survival (DFS) among tumor samples exhibiting elevated *GOLM1* or *B4GALT1* expression (n=89) compared with those with low expression (n=89). Employing the Log-rank test for hypothesis testing, our findings emphasize a noteworthy correlation between heightened gene expression and reduced OS and DFS in the PADD cancer cohort **(Supplementary Figure 2B, C, Supplementary Figure 3B**, C). Consequently, these two proteins were selected as the targets for experimental validation to further investigate their potential roles in PDAC development. Two gene-specific siRNAs (siGOML1 and siB4GAL1) were employed for post-transcriptional gene silencing of *GOML1* and *B4GAL1*, resulting in the knockdown of these two genes. As depicted in **Figure 4A**, qPCR analysis demonstrated a significant reduction in the mRNA expression of *GOML1* and *B4GAL1* in PANC-1 and SU.86.86 cells at 72 hours after transfection with siGOML1 or siB4GAL1 (40 nM) when compared with the untreated control group ($P < 0.05$). No significant difference was observed between the negative control group (NC, Mock-siRNA transfection) and the control groups (**Figure 4A**). This trend was also consistent in the western blot analysis (**Figure 4B**) in comparison with the qPCR assay, indicating that siGOML1 and siB4GAL1 effectively reduce the expression of *GOML1* and *B4GAL1* at both mRNA and protein levels in PANC-1 and SU.86.86 cells.

To assess the biological impact of *GOLM11* and *B4GAL1* silencing in PANC-1 and

SU.86.86 cells, cell proliferation was examined using the MTS assay over a span of five consecutive days. As shown in **Figures 4C** and **4D**, transfection of siGOML1 and siB4GAL1 inhibited cell proliferation in both PANC-1 and SU.86.86 cells compared with the control (untransfected) and NC (Mock-siRNA transfected) groups. Furthermore, a wound healing assay demonstrated that at 12- and 24-hours post-scratch treatment, the open wound area in *GOLM11* and *B4GAL1* siRNA-transfected cells was significantly larger than that in mock siRNA-transfected or untransfected cells (**Figure 4D, 4E**), implying that knockdown of *GOLM11* and *B4GAL1* in PANC-1 and SU.86.86 cells effectively inhibited cell migration *in vitro*. To investigate whether the down-regulation of *GOLML1* and *B4GAL1* affects the invasive capabilities of PANC-1 and SU.86.86 cells, a transwell analysis was performed. The results revealed a significant inhibition of cell invasion in PANC-1 and SU.86.86 cells upon *GOLML1* or *B4GAL1* silencing. The number of siGOML1 or siB4GAL1-transfected cells invading through the membrane was markedly lower than that of control-siRNA transfected cells (**Fig. 4F**, $P < 0.05$). Together, our findings suggest that GOLM1 and B4GT1 play crucial roles in PDAC cell proliferation, migration, and invasion, and their suppression could potentially serve as a therapeutic strategy for PDAC.

**Discussion**

This is the first PWAS study using comprehensive protein genetic prediction models to assess the associations between genetically predicted circulating protein concentrations and PDAC risk. Overall, we identified 40 proteins that were significantly associated with PDAC risk after FDR correction, including 16 novel proteins that have not been previously reported. Our results suggest new knowledge on the genetics and etiology of PDAC, and the newly identified proteins could serve as candidate blood biomarkers for risk assessment of PDAC, a highly fatal

malignancy. We also identified potential drug repurposing opportunities targeting the identified proteins which warrant further investigations.

In previous studies, blood concentrations of specific proteins such as CA242, PIVKA-II, PAM4, S100A6, OPN, RBM6, EphA2, and OPG have been reported to be potentially associated with PDAC risk (4-7). In the INTERVAL dataset, proteins S100A6 and OPG were captured, and we were able to develop satisfactory prediction models for their levels in blood (17). We observed a significant association with the same direction for OPG (*P*-value = 0.03, Z-score = 2.23) but not for S100A6 (*P*-value=0.93) with PDAC risk. Such inconsistent findings with previous studies might be explained by potential biases in previous epidemiological studies and warrant further exploration.

In this large study, we identified 16 novel proteins that were associated with PDAC risk. Previous studies have suggested potential roles for some of the novel proteins in pancreatic tumorigenesis. Tie1 deficiency is reported to induce endothelial–mesenchymal transition (EndMT) and promote a motile phenotype (43). EndMT is known to present in human pancreatic tumors (43). Another study reports that TNF-α that is abundantly present in PDAC, induces EndMT and acts at least partially through TIE1 regulation in murine pancreatic tumors (44). For CPB1, immunohistochemistry of tissue microarray from PDAC patients showed that it was significantly downregulated in pancreatic tumor compared with adjacent normal pancreatic tissues (45). This aligns with the negative association between genetically predicted levels of carboxypeptidase B1 and PDAC risk observed in this study. In another study it was reported that mutations in *CPB1* were associated with pancreatic cancer (46). Regarding GOLM1, one study supported that long non-coding RNA TP73-AS1 could promote pancreatic cancer progression through GOLM1 upregulation by competitively binding to miR-128-3p (47). Further

investigations are warranted to clarify roles of the identified proteins in pancreatic cancer development.

Based on drug repurposing analyses, we prioritized several drugs that may serve as promising candidates for treating PDAC, such as Crizotinib, Cabozantinib, Brigatinib, Capmatinib, Tepotinib, and Tivozanib targeting Met. Previous research has supported potential link between these drugs and PDAC. For example, earlier research found that Crizotinib and Cabozantinib could decrease PDAC cell line viability *in vitro* (48). Cabozantinib together with photodynamic therapy had been shown to achieve local control and decrease in tumor metastases in preclinical PDAC models (49). A translational mathematical modeling study revealed that Tepotinib at a dose selection of 500 mg once daily could be effective for PDAC (50). Further work is needed to assess potential efficacy of these drug candidates in PDAC treatment.

There are several strengths of this study for detecting proteins associated with PDAC risk. We developed comprehensive protein genetic prediction models as instruments, which not only potentially minimize biases commonly encountered in conventional observational study design, but also bring improved statistical power compared with the design of only using pQTLs as instruments. However, several limitations of this study need to be recognized when interpreting our findings. First, our results may still be susceptible to potential pleiotropic effects and may not necessarily infer causality. Similar to the design of transcriptome-wide association study (TWAS), our PWAS should be useful for prioritizing causal proteins; however we cannot completely exclude the possibility of false positive findings for some of the identified associations (51). Several likely reasons may induce these, such as correlated protein expression across participants, correlated genetically predicted protein expression, as well as shared genetic variants (51). Future functional investigation will better characterize whether the identified

proteins play a causal role in PDAC development. Second, since in this work the genetically regulated components of plasma protein levels were studied but not the overall measured levels, the utility of the identified proteins as risk biomarkers for PDAC remains unclear. Additional work for measuring circulating protein levels in pre-diagnostic blood samples are needed to evaluate the prediction role of these proteins in PDAC risk. Third, for our current model development design, the candidate predictors for each protein of interest merely rely on the potentially associated SNPs at a specific statistical threshold. A small proportion of proteins were excluded for downstream model construction because of the lack of such SNPs. Future work considering additional potential predictors beyond such statistics-based selection would be needed to improve the ability to evaluate additional proteins. Fourth, previous work has supported that covariates of smoking and body mass index are related to blood protein levels (52,53). In the current study using INTERVAL resources, we were not able to adjust for these covariates during model construction. Further study is thus needed to validate our results. Lastly, the current study largely focuses on Europeans for both protein genetic prediction model development and downstream association analyses with PDAC risk. Future research is warranted to study proteins associated with PDAC risk in other non-European ancestries.

Our TGCA data analysis has revealed potential relevance of B4GT1 and GOLM1 in tumorigenesis and tumor progression. B4GT1 (Beta-1,4-Galactosyl transferase 1) is an enzyme primarily responsible for catalyzing the galactose transfer to specific receptor molecules within organisms (54). Its significance lies in its involvement in various essential biological processes, such as intercellular communication and cell adhesion. Furthermore, alterations in the expression level of B4GT1 have been observed in certain cancers, suggesting its potential implication in tumor initiation and development (55). This intriguing finding has led us to select B4GT1 as a priority

target for further exploration of its role in PDAC using experimental techniques. Similarly, our attention was drawn to GOLM1 (Golgi Membrane Protein 1), a membrane protein predominantly located in the Golgi apparatus, which plays a pivotal role in cellular secretion and transport processes. Recent investigations have demonstrated an upregulation of GOLM1 expression in multiple cancer types, including liver cancer, lung cancer, and pancreatic cancer. Such evidence strongly suggests that GOLM1 might exert a significant influence on the onset and progression of these malignancies (56). Consequently, we selected GOLM1 as an additional focus for verification to gain deeper insights into its involvement in PDAC. By utilizing RNAi technology to silence these genes, our experimental results corroborated the critical roles of GOLM1 and B4GT1 in driving PDAC cell proliferation, migration, and invasion. Subduing these genes holds promise as a potential therapeutic approach for PDAC treatment.

In summary, using protein genetic prediction models, we identified 16 novel protein biomarker candidates for which the genetically predicted circulating levels were significantly associated with PDAC risk. Future work is needed to better characterize the potential roles of these proteins in the etiology of PDAC development, assess the predictive role of such markers in risk assessment of PDAC, and evaluate whether the potential drug repurposing opportunities we identified may improve PDAC outcomes.

**Data availability**

The pancreatic cancer genetic datasets used for the association analyses described in this manuscript can be obtained from dbGaP [57] (accession numbers phs000206.v5.p3 and phs000648.v1.p1). The INTERVAL individual-level genotype and protein data, and full summary association results from the genetic analysis, are available through the European Genotype Archive (accession number EGAS00001002555). Summary association results are

also publicly available at [58] http://www.phpc.cam.ac.uk/ceu/proteins/, through PhenoScanner [59] http://www.phenoscanner.medschl.cam.ac.uk and from the NHGRI-EBI GWAS Catalog [60]. Other data further supporting this work are openly available in the GigaScience repository, GigaDB [61].

**Abbreviations list:**

Pancreatic ductal adenocarcinoma (PDAC)

protein quantitative trait loci (pQTL)

Genome-wide association studies (GWAS)

the Pancreatic Cancer Cohort Consortium (PanScan)

the Pancreatic Cancer Case-Control Consortium (PanC4)

quality control (QC)

Hardy-Weinberg equilibrium (HWE)

false discovery rate (FDR)

**Competing interests**

L.W. provided consulting service to Pupil Bio Inc. and received honorarium. No potential conflicts of interest were disclosed by the other authors.

**Funding**

This study is supported by the University of Hawai‘i Cancer Center and V Foundation V Scholar Award. Lang Wu and Chong Wu are supported by NCI R01CA263494. Lang Wu is also supported by NHGRI/NIMHD U54HG013243 and NCI R00CA218892. This work was supported in part by NIH-NIMHD U54MD007598, NIH/NCI1U54CA14393, U56 CA101599-01; Department-of-Defense Breast Cancer Research Program grant BC043180, NIH/NCATS

CTSI UL1TR000124 to J.V. Vadgama; Accelerating Excellence in Translational Science Pilot Grants G0812D05, NIH/NCI SC1CA200517 and 9 SC1 GM135050-05 to Y. Wu. Qizhi Yao is supported by VA Merit Award 1 I01 CX001822-01A2 (PI: Yao). The PanScan study was funded in whole or in part with federal funds from the National Cancer Institute (NCI), US National Institutes of Health (NIH) under contract number HHSN261200800001E. Additional support was received from NIH/NCI K07 CA140790, the American Society of Clinical Oncology Conquer Cancer Foundation, the Howard Hughes Medical Institute, the Lustgarten Foundation, the Robert T. and Judith B. Hale Fund for Pancreatic Cancer Research and Promises for Purple. A full list of acknowledgments for each participating study is provided in the Supplementary Note of the manuscript with PubMed ID: 25086665. For the PanC4 GWAS study, the patients and controls were derived from the following PANC4 studies: Johns Hopkins National Familial Pancreas Tumor Registry, Mayo Clinic Biospecimen Resource for Pancreas Research, Ontario Pancreas Cancer Study (OPCS), Yale University, MD Anderson Case Control Study, Queensland Pancreatic Cancer Study, University of California San Francisco Molecular Epidemiology of Pancreatic Cancer Study, International Agency of Cancer Research and Memorial Sloan Kettering Cancer Center. This work is supported by NCI R01CA154823 Genotyping services were provided by the Center for Inherited Disease Research (CIDR). CIDR is fully funded through a federal contract from the National Institutes of Health to The Johns Hopkins University, contract number HHSN2682011000111. The content is solely the responsibility of the authors and does not necessarily represent the official views of the National Institutes of Health. Participants in the INTERVAL randomised controlled trial were recruited with the active collaboration of NHS Blood and Transplant England (www.nhsbt.nhs.uk), which has supported field work and other elements of the trial. DNA extraction and genotyping were

co-funded by the National Institute for Health Research (NIHR), the NIHR BioResource (http://bioresource.nihr.ac.uk) and the NIHR Cambridge Biomedical Research Centre (BRC-1215-20014) [*]. The academic coordinating centre for INTERVAL was supported by core funding from the: NIHR Blood and Transplant Research Unit in Donor Health and Genomics (NIHR BTRU-2014-10024), UK Medical Research Council (MR/L003120/1), British Heart Foundation (SP/09/002; RG/13/13/30194; RG/18/13/33946) and NIHR Cambridge BRC (BRC-1215-20014) [*]. A complete list of the investigators and contributors to the INTERVAL trial is provided in reference [**]. The academic coordinating centre would like to thank blood donor centre staff and blood donors for participating in the INTERVAL trial.

*The views expressed are those of the author(s) and not necessarily those of the NIHR or the Department of Health and Social Care.

**Di Angelantonio E, Thompson SG, Kaptoge SK, Moore C, Walker M, Armitage J, Ouwehand WH, Roberts DJ, Danesh J, INTERVAL Trial Group. Efficiency and safety of varying the frequency of whole blood donation (INTERVAL): a randomised trial of 45 000 donors. Lancet. 2017 Nov 25;390(10110):2360-2371.

**Author contributions**

L.W. conceived the study. Y.W. designed the functional experiments and supervised the *in vitro* functional work. C.W. and J.Z. contributed to the study design and/or prediction model building. S.L. performed model building and statistical analyses. D.H.G. contributed to statistical analyses. K.W. conducted *in vitro* functional work. J.Z. performed the drug repurposing curation. M.A.A.

performed molecular docking analysis. H.Z. and S. L. contributed to the bioinformatics and pathway analyses. L.W., J.Z., K.W., Y.W., A.M., H.Z., and T.Y. wrote the first version of manuscript. D.H.G., P.S., T.L., E.P., Q.Y., T.L., S.F., J.V.V., H-W. D., Y.D., H.Z., S.L., and A.B. contributed to manuscript revision and/or INTERVAL data management. All authors have reviewed and approved the final manuscript.

**Acknowledgements**

The authors also would like to thank all the individuals for their participation in the parent studies and all the researchers, clinicians, technicians and administrative staff for their contribution to the studies.

## Figure legends

**Figure 1.** The overall design of this study.

**Figure 2.** Manhattan plot of 40 identified proteins associated with PDAC risk. Proteins with blue color represent those identified in our previous work using pQTL as instruments, and proteins with red color represent novel ones identified in the current study.

**Figure 3.** PPI network and canonical pathways of 40 identified proteins associated with PDAC risk. Network nodes represent proteins; edge thickness is proportional to the evidence for the PPI;

and dashed lines represent the interaction among clusters. The enrichment of canonical pathways was determined using IPA software.

**Figure 4.** The analysis of cell proliferation, migration and invasion on PANC-1 and SU.86.86 cells with siB4GLAT1 and siGOLM1 transfection. The quantitative real-time PCR (qPCR) assay and the western blot assay (A) were used to investigate the RNAi effect of siB4GLAT1 and siGOLM1 (40 nM, 72 h) in PANC-1 and SU.86.86 cells. GAPDH were used as an internal control for qPCR analyses and western blot analyses, respectively (B,C) The effect of transfection with siB4GLAT1 and siGOLM1 (40 nM) on cell proliferation. The cells were detected by MTS [3-(4,5-dimethylthiazol-2-yl)-5-(3-carboxymethoxyphenyl)-2-(4-sulfophenyl)-2H-tetrazolium] assay on each day for 5 consecutive days. (D,E) Silencing of *B4GLAT1* and *GOLM1* inhibited migration of PANC-1 and SU.86.86 cells. Representative images of wound scratch assay performed to evaluate the motility of cells after silencing *B4GLAT1* and *GOLM1*. After transfection, a scratch was made on cells monolayer and was monitored with microscopy every 12 hours (0, 12, and 24 h). Bar graphs show normalized wound area, calculated using Gen 5. Representative images of invasion assay. Data are represented as mean ± SD from triplicate samples, where $*p < 0.01$ compared to the control. (F) Effect of siB4GLAT1 and siGOLM1 transfection on the invasion of PANC-1 and SU.86.86 cells. After siB4GLAT1 and siGOLM1 transfection for 48 h, invasive ability of PANC-1 and SU.86.86 cells was identified by transwell assay. $**P < 0.01$ compared with the control cells; $^{##}P < 0.01$ compared with the mock cells; data are expressed as the mean ± SD, n = 3.

## References


1. Sung H, Ferlay J, Siegel RL, Laversanne M, Soerjomataram I, Jemal A, *et al.* Global Cancer Statistics 2020: GLOBOCAN Estimates of Incidence and Mortality Worldwide for 36 Cancers in 185 Countries. CA Cancer J Clin **2021**;71(3):209-49 doi 10.3322/caac.21660.
2. Rawla P, Sunkara T, Gaduputi V. Epidemiology of Pancreatic Cancer: Global Trends, Etiology and Risk Factors. World J Oncol **2019**;10(1):10-27 doi 10.14740/wjon1166.
3. Ballehaninna UK, Chamberlain RS. The clinical utility of serum CA 19-9 in the diagnosis, prognosis and management of pancreatic adenocarcinoma: An evidence based appraisal. J Gastrointest Oncol **2012**;3(2):105-19 doi 10.3978/j.issn.2078-6891.2011.021.
4. Tartaglione S, Pecorella I, Zarrillo SR, Granato T, Viggiani V, Manganaro L, *et al.* Protein Induced by Vitamin K Absence II (PIVKA-II) as a potential serological biomarker in pancreatic cancer: a pilot study. Biochem Med (Zagreb) **2019**;29(2):020707 doi 10.11613/BM.2019.020707.
5. Duan B, Hu X, Fan M, Xiong X, Han L, Wang Z, *et al.* RNA-Binding Motif Protein 6 is a Candidate Serum Biomarker for Pancreatic Cancer. Proteomics Clin Appl **2019**;13(5):e1900048 doi 10.1002/prca.201900048.
6. Koshikawa N, Minegishi T, Kiyokawa H, Seiki M. Specific detection of soluble EphA2 fragments in blood as a new biomarker for pancreatic cancer. Cell Death Dis **2017**;8(10):e3134 doi 10.1038/cddis.2017.545.
7. Loosen SH, Neumann UP, Trautwein C, Roderburg C, Luedde T. Current and future biomarkers for pancreatic adenocarcinoma. Tumour Biol **2017**;39(6):1010428317692231 doi 10.1177/1010428317692231.
8. Didelez V, Sheehan N. Mendelian randomization as an instrumental variable approach to causal inference. Stat Methods Med Res **2007**;16(4):309-30 doi 10.1177/0962280206077743.
9. Lawlor DA, Harbord RM, Sterne JA, Timpson N, Davey Smith G. Mendelian randomization: using genes as instruments for making causal inferences in epidemiology. Stat Med **2008**;27(8):1133-63 doi 10.1002/sim.3034.

10. Sun BB, Maranville JC, Peters JE, Stacey D, Staley JR, Blackshaw J, *et al.* Genomic atlas of the human plasma proteome. Nature **2018**;558(7708):73-9 doi 10.1038/s41586-018-0175-2.
11. Wu L, Shu X, Bao J, Guo X, Kote-Jarai Z, Haiman CA, *et al.* Analysis of Over 140,000 European Descendants Identifies Genetically Predicted Blood Protein Biomarkers Associated with Prostate Cancer Risk. Cancer Res **2019**;79(18):4592-8 doi 10.1158/0008-5472.CAN-18-3997.
12. Zhu J, Wu C, Wu L. Associations Between Genetically Predicted Protein Levels and COVID-19 Severity. J Infect Dis **2021**;223(1):19-22 doi 10.1093/infdis/jiaa660.
13. Zhu J, O'Mara TA, Liu D, Setiawan VW, Glubb D, Spurdle AB, *et al.* Associations between Genetically Predicted Circulating Protein Concentrations and Endometrial Cancer Risk. Cancers (Basel) **2021**;13(9) doi 10.3390/cancers13092088.
14. Shu X, Bao J, Wu L, Long J, Shu XO, Guo X, *et al.* Evaluation of associations between genetically predicted circulating protein biomarkers and breast cancer risk. Int J Cancer **2020**;146(8):2130-8 doi 10.1002/ijc.32542.
15. Gusev A, Ko A, Shi H, Bhatia G, Chung W, Penninx BW, *et al.* Integrative approaches for large-scale transcriptome-wide association studies. Nat Genet **2016**;48(3):245-52 doi 10.1038/ng.3506.
16. Wu L, Yang Y, Guo X, Shu XO, Cai Q, Shu X, *et al.* An integrative multi-omics analysis to identify candidate DNA methylation biomarkers related to prostate cancer risk. Nat Commun **2020**;11(1):3905 doi 10.1038/s41467-020-17673-9.
17. Liu D, Zhou D, Sun Y, Zhu J, Ghoneim D, Wu C, *et al.* A Transcriptome-Wide Association Study Identifies Candidate Susceptibility Genes for Pancreatic Cancer Risk. Cancer Res **2020**;80(20):4346-54 doi 10.1158/0008-5472.CAN-20-1353.
18. Sun Y, Zhu J, Zhou D, Canchi S, Wu C, Cox NJ, *et al.* A transcriptome-wide association study of Alzheimer's disease using prediction models of relevant tissues identifies novel candidate susceptibility genes. Genome Med **2021**;13(1):141 doi 10.1186/s13073-021-00959-y.
19. Sun Y, Zhou D, Rahman MR, Zhu J, Ghoneim D, Cox NJ, *et al.* A transcriptome-wide association study identifies novel blood-based gene biomarker candidates for Alzheimer's disease risk. Hum Mol Genet **2021**;31(2):289-99 doi 10.1093/hmg/ddab229.
20. Zhu J, Yang Y, Kisiel JB, Mahoney DW, Michaud DS, Guo X, *et al.* Integrating Genome and Methylome Data to Identify Candidate DNA Methylation Biomarkers for Pancreatic Cancer Risk. Cancer Epidemiol Biomarkers Prev **2021**;30(11):2079-87 doi 10.1158/1055-9965.EPI-21-0400.
21. Liu D, Zhu J, Zhou D, Nikas EG, Mitanis NT, Sun Y, *et al.* A transcriptome-wide association study identifies novel candidate susceptibility genes for prostate cancer risk. Int J Cancer **2022**;150(1):80-90 doi 10.1002/ijc.33808.
22. Sun Y, Bae YE, Zhu J, Zhang Z, Zhong H, Yu J, *et al.* A splicing transcriptome-wide association study identifies novel altered splicing for Alzheimer's disease susceptibility. Neurobiol Dis **2023**;184:106209 doi 10.1016/j.nbd.2023.106209.
23. Sun Y, Bae YE, Zhu J, Zhang Z, Zhong H, Cheng C, *et al.* A Splicing Transcriptome-Wide Association Study Identifies Candidate Altered Splicing for Prostate Cancer Risk. OMICS **2023**;27(8):372-80 doi 10.1089/omi.2023.0065.
24. Sun Y, Zhu J, Yang Y, Zhang Z, Zhong H, Zeng G, *et al.* Identification of candidate DNA methylation biomarkers related to Alzheimer's disease risk by integrating genome

and blood methylome data. Transl Psychiatry **2023**;13(1):387 doi 10.1038/s41398-023-02695-w.

25. Liu D, Bae YE, Zhu J, Zhang Z, Sun Y, Deng Y, *et al.* Splicing transcriptome-wide association study to identify splicing events for pancreatic cancer risk. Carcinogenesis **2023**;44(10-11):741-7 doi 10.1093/carcin/bgad069.
26. Liu S, Zhong H, Zhu J, Wu Y, Deng Y, Wu L. Regulome-wide association study identifies genetically driven accessible regions associated with pancreatic cancer risk. Int J Cancer **2024**;154(4):670-8 doi 10.1002/ijc.34761.
27. Liu S, Zhong H, Zhu J, Wu L. Identification of blood metabolites associated with risk of Alzheimer's disease by integrating genomics and metabolomics data. Mol Psychiatry **2024** doi 10.1038/s41380-023-02400-9.
28. Zhu J, Liu S, Walker KA, Zhong H, Ghoneim DH, Zhang Z, *et al.* Associations between genetically predicted plasma protein levels and Alzheimer's disease risk: a study using genetic prediction models. Alzheimers Res Ther **2024**;16(1):8 doi 10.1186/s13195-023-01378-4.
29. Zhong H, Liu S, Zhu J, Wu L. Associations between genetically predicted levels of blood metabolites and pancreatic cancer risk. Int J Cancer **2023**;153(1):103-10 doi 10.1002/ijc.34466.
30. Zhong H, Zhu J, Liu S, Ghoneim DH, Surendran P, Liu T, *et al.* Identification of blood protein biomarkers associated with prostate cancer risk using genetic prediction models: analysis of over 140,000 subjects. Hum Mol Genet **2023**;32(22):3181-93 doi 10.1093/hmg/ddad139.
31. Yang J, Lee SH, Goddard ME, Visscher PM. GCTA: a tool for genome-wide complex trait analysis. Am J Hum Genet **2011**;88(1):76-82 doi 10.1016/j.ajhg.2010.11.011.
32. Klein AP, Wolpin BM, Risch HA, Stolzenberg-Solomon RZ, Mocci E, Zhang M, *et al.* Genome-wide meta-analysis identifies five new susceptibility loci for pancreatic cancer. Nat Commun **2018**;9(1):556 doi 10.1038/s41467-018-02942-5.
33. McCarthy S, Das S, Kretzschmar W, Delaneau O, Wood AR, Teumer A, *et al.* A reference panel of 64,976 haplotypes for genotype imputation. Nat Genet **2016**;48(10):1279-83 doi 10.1038/ng.3643.
34. Howie BN, Donnelly P, Marchini J. A flexible and accurate genotype imputation method for the next generation of genome-wide association studies. PLoS Genet **2009**;5(6):e1000529 doi 10.1371/journal.pgen.1000529.
35. Kramer A, Green J, Pollard J, Jr., Tugendreich S. Causal analysis approaches in Ingenuity Pathway Analysis. Bioinformatics **2014**;30(4):523-30 doi 10.1093/bioinformatics/btt703.
36. Szklarczyk D, Gable AL, Nastou KC, Lyon D, Kirsch R, Pyysalo S, *et al.* The STRING database in 2021: customizable protein-protein networks, and functional characterization of user-uploaded gene/measurement sets. Nucleic Acids Res **2021**;49(D1):D605-D12 doi 10.1093/nar/gkaa1074.
37. Koscielny G, An P, Carvalho-Silva D, Cham JA, Fumis L, Gasparyan R, *et al.* Open Targets: a platform for therapeutic target identification and validation. Nucleic Acids Res **2017**;45(D1):D985-D94 doi 10.1093/nar/gkw1055.
38. Wishart DS, Knox C, Guo AC, Shrivastava S, Hassanali M, Stothard P, *et al.* DrugBank: a comprehensive resource for in silico drug discovery and exploration. Nucleic Acids Res **2006**;34(Database issue):D668-72 doi 10.1093/nar/gkj067.

39. Alam MA SH, Deng H-W. A robust kernel machine regression towards biomarker selection in multi-omics datasets of osteoporosis for drug discovery. In: University T, editor. arXiv2022.
40. Kim S, Chen J, Cheng T, Gindulyte A, He J, He S, *et al.* PubChem 2019 update: improved access to chemical data. Nucleic Acids Res **2019**;47(D1):D1102-D9 doi 10.1093/nar/gky1033.
41. Kim SY, Jeong HH, Kim J, Moon JH, Sohn KA. Robust pathway-based multi-omics data integration using directed random walks for survival prediction in multiple cancer studies. Biol Direct **2019**;14(1):8 doi 10.1186/s13062-019-0239-8.
42. Zhu J, Shu X, Guo X, Liu D, Bao J, Milne RL, *et al.* Associations between Genetically Predicted Blood Protein Biomarkers and Pancreatic Cancer Risk. Cancer Epidemiol Biomarkers Prev **2020**;29(7):1501-8 doi 10.1158/1055-9965.EPI-20-0091.
43. Garcia J, Sandi MJ, Cordelier P, Binetruy B, Pouyssegur J, Iovanna JL, *et al.* Tie1 deficiency induces endothelial-mesenchymal transition. EMBO Rep **2012**;13(5):431-9 doi 10.1038/embor.2012.29.
44. Adjuto-Saccone M, Soubeyran P, Garcia J, Audebert S, Camoin L, Rubis M, *et al.* TNF-alpha induces endothelial-mesenchymal transition promoting stromal development of pancreatic adenocarcinoma. Cell Death Dis **2021**;12(7):649 doi 10.1038/s41419-021-03920-4.
45. Song Y, Wang Q, Wang D, Junqiang L, Yang J, Li H, *et al.* Label-Free Quantitative Proteomics Unravels Carboxypeptidases as the Novel Biomarker in Pancreatic Ductal Adenocarcinoma. Transl Oncol **2018**;11(3):691-9 doi 10.1016/j.tranon.2018.03.005.
46. Tamura K, Yu J, Hata T, Suenaga M, Shindo K, Abe T, *et al.* Mutations in the pancreatic secretory enzymes CPA1 and CPB1 are associated with pancreatic cancer. Proc Natl Acad Sci U S A **2018**;115(18):4767-72 doi 10.1073/pnas.1720588115.
47. Wang B, Sun X, Huang KJ, Zhou LS, Qiu ZJ. Long non-coding RNA TP73-AS1 promotes pancreatic cancer growth and metastasis through miRNA-128-3p/GOLM1 axis. World J Gastroenterol **2021**;27(17):1993-2014 doi 10.3748/wjg.v27.i17.1993.
48. Escorcia FE, Houghton JL, Abdel-Atti D, Pereira PR, Cho A, Gutsche NT, *et al.* ImmunoPET Predicts Response to Met-targeted Radioligand Therapy in Models of Pancreatic Cancer Resistant to Met Kinase Inhibitors. Theranostics **2020**;10(1):151-65 doi 10.7150/thno.37098.
49. Broekgaarden M, Alkhateeb A, Bano S, Bulin AL, Obaid G, Rizvi I, *et al.* Cabozantinib Inhibits Photodynamic Therapy-Induced Auto- and Paracrine MET Signaling in Heterotypic Pancreatic Microtumors. Cancers (Basel) **2020**;12(6) doi 10.3390/cancers12061401.
50. Xiong W, Friese-Hamim M, Johne A, Stroh C, Klevesath M, Falchook GS, *et al.* Translational pharmacokinetic-pharmacodynamic modeling of preclinical and clinical data of the oral MET inhibitor tepotinib to determine the recommended phase II dose. CPT Pharmacometrics Syst Pharmacol **2021**;10(5):428-40 doi 10.1002/psp4.12602.
51. Wainberg M, Sinnott-Armstrong N, Mancuso N, Barbeira AN, Knowles DA, Golan D, *et al.* Opportunities and challenges for transcriptome-wide association studies. Nature genetics **2019**;51(4):592-9.
52. Madhuvanthi M, Lathadevi GV. Serum Proteins Alteration in Association with Body Mass Index in Human Volunteers. J Clin Diagn Res **2016**;10(6):CC05-7 doi 10.7860/JCDR/2016/18278.8047.

53. Gallus S, Lugo A, Suatoni P, Taverna F, Bertocchi E, Boffi R, *et al.* Effect of Tobacco Smoking Cessation on C-Reactive Protein Levels in A Cohort of Low-Dose Computed Tomography Screening Participants. Sci Rep **2018**;8(1):12908 doi 10.1038/s41598-018-29867-9.
54. Morokuma D, Xu J, Hino M, Mon H, Merzaban JS, Takahashi M, *et al.* Expression and Characterization of Human beta-1, 4-Galactosyltransferase 1 (beta4GalT1) Using Silkworm-Baculovirus Expression System. Mol Biotechnol **2017**;59(4-5):151-8 doi 10.1007/s12033-017-0003-1.
55. Cui Y, Li J, Zhang P, Yin D, Wang Z, Dai J, *et al.* B4GALT1 promotes immune escape by regulating the expression of PD-L1 at multiple levels in lung adenocarcinoma. J Exp Clin Cancer Res **2023**;42(1):146 doi 10.1186/s13046-023-02711-3.
56. Liu Y, Hu X, Liu S, Zhou S, Chen Z, Jin H. Golgi Phosphoprotein 73: The Driver of Epithelial-Mesenchymal Transition in Cancer. Front Oncol **2021**;11:783860 doi 10.3389/fonc.2021.783860.
57. dbGAP: https://www.ncbi.nlm.nih.gov/gap/, Accessed on 1 March 2024.
58. http://www.phpc.cam.ac.uk/ceu/proteins/, Accessed 15 February 2024.
59. PhenoScanner: http://www.phenoscanner.medschl.cam.ac.uk), Accessed 5 March 2024
60. NHGRI-EBI GWAS Catalog: https://www.ebi.ac.uk/gwas/downloads/summary-statistics, Accessed 10 February 2024.
61. Zhu J; Wu K; Liu S; Masca A; Zhong H; Yang T; Ghoneim DH; Surendran P; Liu T; Yao Q; Liu T; Fahle S; Butterworth A; Alam MA; Vadgama JV; Deng Y; Deng H; Wu C; Wu Y; Wu L: Supporting data for "Proteome-wide association study and functional validation identify novel protein markers for pancreatic ductal adenocarcinoma" GigaScience Database. 2024. http://dx.doi.org/10.5524/102502

**Table 1**. Novel proteins with genetically predicted concentrations in plasma to be associated with pancreatic cancer risk

| Protein | SOMAmer ID | Protein full name | Protein-encoding gene | Region for protein encoding gene | Prediction model method | Heritability | Number of Predicting SNPs | Number of Predicting SNPs-Cis* | Number of Predicting SNPs-Trans | Model internal cross validation $R^2$ | Model external validation $R^2$ | Z-value[a] | *P*-value[a] | FDR *P*-value[b] |
|---|---|---|---|---|---|---|---|---|---|---|---|---|---|---|
| IL-23 R | IL23R.5088.175.3 | Interleukin-23 receptor | *IL23R* | 1p31.3 | elastic net | 0.06 | 24 | 24 | 0 | 0.04 | 0.04 | 3.55 | $3.80\times10^{-4}$ | 0.02 |
| sTie-1 | TIE1.2844.53.2 | Tyrosine-Protein Kinase Receptor Tie-1, Soluble | *TIE1* | 1p34.2 | lasso | 0.2 | 18 | 7 | 11 | 0.22 | 0.28 | 5.67 | $1.46\times10^{-8}$ | $1.22\times10^{-6}$ |
| FA20B | FAM20B.7198.197.3 | Glycosaminoglycan Xylosylkinase | *FAM20B* | 1q25.2 | lasso | 0.05 | 8 | 5 | 3 | 0.02 | 0.04 | 5.30 | $1.17\times10^{-7}$ | $7.82\times10^{-6}$ |
| FAM3D | FAM3D.13102.1.3 | Protein FAM3D | *FAM3D* | 3p14.2 | elastic net | 0.27 | 58 | 16 | 42 | 0.37 | 0.36 | 6.10 | $1.07\times10^{-9}$ | $1.02\times10^{-7}$ |
| Carboxypeptidase B1 | CPB1.6356.3.3 | Carboxypeptidase B | *CPB1* | 3q24 | lasso | 0.07 | 7 | 3 | 4 | 0.04 | 0.03 | -4.55 | $5.38\times10^{-6}$ | $3.00\times10^{-4}$ |
| RAP | LRPAP1.3640.14.3 | alpha-2-macroglobulin receptor-associated protein | *LRPAP1* | 4p16.3 | elastic net | 0.47 | 168 | 23 | 145 | 0.27 | 0.22 | 3.21 | 0.001 | 0.04 |
| Semaphorin-6A | SEMA6A.7945.10.3 | Semaphorin-6A | *SEMA6A* | 5q23.1 | elastic net | 0.11 | 66 | 44 | 22 | 0.05 | 0.05 | -3.57 | $3.54\times10^{-4}$ | 0.02 |
| B4GT1 | B4GALT1.13381.49.3 | Beta-1,4-galactosyltransferase 1 | *B4GALT1* | 9p21.1 | elastic net | 0.10 | 39 | 16 | 23 | 0.08 | 0.10 | 4.65 | $3.29\times10^{-6}$ | $1.96\times10^{-4}$ |
| GOLM1 | GOLM1.8983.7.3 | Golgi Membrane Protein 1 | *GOLM1* | 9q21.33 | lasso | 0.11 | 10 | 0 | 10 | 0.14 | 0.17 | 8.07 | $7.12\times10^{-16}$ | $2.14\times10^{-13}$ |
| QSOX2 | QSOX2.8397.147.3 | Sulfhydryl oxidase 2 | *QSOX2* | 9q34.3 | elastic net | 0.31 | 28 | 10 | 18 | 0.40 | 0.40 | 7.98 | $1.44\times10^{-15}$ | $2.75\times10^{-13}$ |
| KIN17 | KIN.14643.27.3 | DNA/RNA-binding protein KIN17 | *KIN* | 10p14 | elastic net | 0.08 | 29 | 0 | 29 | 0.05 | 0.07 | -5.52 | $3.31\times10^{-8}$ | $2.60\times10^{-6}$ |
| ISLR2 | ISLR2.13124.20.3 | Immunoglobulin superfamily containing leucine-rich repeat protein 2 | *ISLR2* | 15q24.1 | elastic net | 0.17 | 77 | 32 | 45 | 0.14 | 0.13 | -3.45 | $5.65\times10^{-4}$ | 0.02 |
| DPEP2 | DPEP2.8327.26.3 | Dipeptidase 2 | *DPEP2* | 16q22.1 | elastic net | 0.07 | 36 | 0 | 36 | 0.06 | 0.05 | -4.01 | $5.97\times10^{-5}$ | 0.003 |
| Chymotrypsin | CTRB1.5671.1.3 | Chymotrypsinogen B | *CTRB1* | 16q23.1 | elastic net | 0.35 | 85 | 69 | 16 | 0.23 | 0.24 | -4.32 | $1.59\times10^{-5}$ | $8.50\times10^{-4}$ |

| | | | | | | | | | | | | | | |
|---|---|---|---|---|---|---|---|---|---|---|---|---|---|---|
| Laminin | LAMA1.LAMB1.LAMC1.2728.62.2 | Laminin | *LAMA1, LAMB1, LAMC1* | 18p11.31, 7q31.1, 1q25.3 | elastic net | 0.09 | 62 | 14 | 48 | 0.08 | 0.05 | 3.88 | $1.06\times10^{-4}$ | 0.005 |
| TPST2 | TPST2.8024.64.3 | Protein-Tyrosine Sulfotransferase 2 | *TPST2* | 22q12.1 | elastic net | 0.08 | 52 | 28 | 24 | 0.07 | 0.08 | 5.88 | $4.16\times10^{-9}$ | $3.71\times10^{-7}$ |

***** SNPs within 1MB of the protein-encoding gene

a Associations between genetically predicted protein levels and PDAC risk after adjustment for age, sex, and top 10 principle components.

b FDR *P*-value: false discovery rate (FDR) adjusted *P*-value; associations with a FDR $p\leq0.05$ considered statistically significant

**Table 2**. Previously reported proteins with genetically predicted concentrations in plasma to be associated with pancreatic cancer risk

| Protein | SOMAmer ID | Protein full name | Protein-encoding gene | Region for protein encoding gene | Prediction model method | Heritability | Number of Predicting SNPs | Number of Predicting SNPs-Cis* | Number of Predicting SNPs-Trans | Model internal cross validation $R^2$ | Model external validation $R^2$ | Z-value[a] | *P*-value[a] | FDR *P*-value[b] |
|---|---|---|---|---|---|---|---|---|---|---|---|---|---|---|
| sE-Selectin | SELE.3470.1.2 | E-selectin | *SELE* | 1q24.2 | lasso | 0.30 | 6 | 0 | 6 | 0.39 | 0.44 | -7.88 | $3.33\times10^{-15}$ | $5.47\times10^{-13}$ |
| P-Selectin | SELP.4154.57.2 | P-Selectin | *SELP* | 1q24.2 | lasso | 0.33 | 11 | 7 | 4 | 0.26 | 0.27 | -3.77 | $1.66\times10^{-4}$ | 0.008 |
| LMA2L | LMAN2L.8013.9.3 | VIP36-like protein | *LMAN2L* | 2q11.2 | top1 | 0.04 | 1 | 1 | 0 | 0.03 | 0.02 | 3.35 | $8.01\times10^{-4}$ | 0.03 |
| Alkaline phosphatase, intestine | ALPI.10463.23.3 | Intestinal-type alkaline phosphatase | *ALPI* | 2q37.1 | lasso | 0.03 | 8 | 0 | 8 | 0.03 | 0.06 | -6.79 | $1.09\times10^{-11}$ | $1.21\times10^{-9}$ |
| VEGF sR2 | KDR.3651.50.5 | Vascular endothelial growth factor receptor 2 | *KDR* | 4q12 | elastic net | 0.29 | 56 | 18 | 38 | 0.18 | 0.12 | -6.21 | $5.22\times10^{-10}$ | $5.37\times10^{-8}$ |
| ADH1B | ADH1B.9834.62.3 | Alcohol dehydrogenase 1B | *ADH1B* | 4q23 | lasso | 0.12 | 6 | 0 | 6 | 0.08 | 0.03 | 3.21 | 0.001 | 0.04 |
| LIF sR | LIFR.5837.49.3 | Leukemia inhibitory factor receptor | *LIFR* | 5p13.1 | top1 | 0.04 | 1 | 0 | 1 | 0.03 | 0.02 | -7.39 | $1.42\times10^{-13}$ | $1.73\times10^{-11}$ |
| gp130, soluble | IL6ST.2620.4.2 | Interleukin-6 receptor subunit beta | *IL6ST* | 5q11.2 | elastic net | 0.08 | 51 | 21 | 30 | 0.06 | 0.05 | -3.69 | $2.22\times10^{-4}$ | 0.01 |
| GP116 | ADGRF5.6409.57.3 | Adhesion G protein-coupled receptor F5 | *ADGRF5* | 6p12.3 | lasso | 0.42 | 22 | 15 | 7 | 0.46 | 0.43 | -4.65 | $3.37\times10^{-6}$ | $1.96\times10^{-4}$ |
| CD36 ANTIGEN | CD36.2973.15.2 | Platelet glycoprotein 4 | *CD36* | 7q21.11 | top1 | 0.04 | 1 | 0 | 1 | 0.03 | 0.05 | 3.31 | $9.25\times10^{-4}$ | 0.03 |
| Met | MET.2837.3.2 | Hepatocyte growth factor receptor | *MET* | 7q31 | blup | 0.09 | 1,668 | 603 | 1,065 | 0.07 | 0.04 | -5.06 | $4.27\times10^{-7}$ | $2.72\times10^{-5}$ |
| STOM | STOM.8261.51.3 | Erythrocyte band 7 integral membrane protein | *STOM* | 9q33.2 | lasso | 0.10 | 5 | 0 | 5 | 0.11 | 0.05 | 3.31 | $9.18\times10^{-4}$ | 0.03 |
| BGAT | ABO.9253.52.3 | Histo-blood group ABO system transferase | *ABO* | 9q34.2 | blup | 0.55 | 2,473 | 2,347 | 126 | 0.72 | 0.72 | 9.18 | $4.20\times10^{-20}$ | $5.62\times10^{-17}$ |
| Notch 1 | NOTCH1.5107.7.2 | Neurogenic locus notch homolog protein 1 | *NOTCH1* | 9q34.3 | top1 | 0.02 | 1 | 0 | 1 | 0.01 | 0.02 | 3.29 | $9.97\times10^{-4}$ | 0.04 |

| | | | | | | | | | | | | | | |
|---|---|---|---|---|---|---|---|---|---|---|---|---|---|---|
| Endoglin | ENG.4908.6.1 | Endoglin | *ENG* | 9q34.11 | top1 | 0.02 | 1 | 0 | 1 | 0.01 | 0.01 | -8.04 | $8.93\times10^{-16}$ | $2.14\times10^{-13}$ |
| ST4S6 | CHST15.4469.78.2 | Carbohydrate sulfotransferase 15 | *CHST15* | 10q26.13 | lasso | 0.05 | 5 | 1 | 4 | 0.05 | 0.03 | -8.62 | $6.46\times10^{-18}$ | $4.32\times10^{-15}$ |
| | CHST15.14097.86.3 | | | | lasso | 0.06 | 9 | 2 | 7 | 0.04 | 0.02 | -8.03 | $9.60\times10^{-16}$ | $2.14\times10^{-13}$ |
| CHSTB | CHST11.7779.86.3 | Carbohydrate sulfotransferase 11 | *CHST11* | 12q23.3 | elastic net | 0.15 | 69 | 46 | 23 | 0.11 | 0.07 | 3.52 | $4.25\times10^{-4}$ | 0.02 |
| THSD1 | THSD1.5621.64.3 | Thrombospondin type-1 domain-containing protein 1 | *THSD1* | 13q14.3 | elastic net | 0.07 | 44 | 27 | 17 | 0.04 | 0.03 | -5.34 | $9.41\times10^{-8}$ | $6.62\times10^{-6}$ |
| GLCE | GLCE.7808.5.3 | D-glucuronyl C5-epimerase | *GLCE* | 15q23 | lasso | 0.27 | 11 | 6 | 5 | 0.36 | 0.34 | 4.18 | $2.94\times10^{-5}$ | 0.002 |
| IGF-I sR | IGF1R.4232.19.2 | Insulin-like growth factor 1 receptor | *IGF1R* | 15q26.3 | top1 | 0.01 | 1 | 0 | 1 | 0.01 | 0.02 | -7.39 | $1.42\times10^{-13}$ | $1.73\times10^{-11}$ |
| Desmoglein-2 | DSG2.9484.75.3 | Desmoglein-2 | *DSG2* | 18q12.1 | elastic net | 0.06 | 66 | 44 | 22 | 0.04 | 0.06 | 5.34 | $9.18\times10^{-8}$ | $6.62\times10^{-6}$ |
| DC-SIGN | CD209.3029.52.2 | CD209 Antigen | *CD209* | 19p13.2 | elastic net | 0.30 | 58 | 26 | 32 | 0.39 | 0.38 | 8.52 | $1.62\times10^{-17}$ | $7.22\times10^{-15}$ |
| IR | INSR.3448.13.2 | Insulin receptor | *INSR* | 19p13.2 | lasso | 0.09 | 7 | 0 | 7 | 0.09 | 0.12 | -7.53 | $4.98\times10^{-14}$ | $7.40\times10^{-12}$ |

***** SNPs within 1MB of the protein-encoding gene

a Associations between genetically predicted protein levels and PDAC risk after adjustment for age, sex, and top 10 principle components.

b FDR *P*-value: false discovery rate (FDR) adjusted *P*-value; associations with a FDR $p \leq 0.05$ considered statistically significant

**Table 3**. Drug repurposing opportunities

| Protein | Protein full name | Protein-encoding gene | OpenTargets information (overall score) | Drugbank ID | Drug name | Molecular action | Molecular docking score* |
|---|---|---|---|---|---|---|---|
| sTie-1 | Tyrosine-Protein Kinase Receptor Tie-1, Soluble | *TIE1* | 0.006 | DB12010 | Fostamatinib | inhibitor | -6.1 |
| Carboxypeptidase B1 | Carboxypeptidase B | *CPB1* | 0.159 | DB04272 | Citric acid | NA | -3.9 |
| Chymotrypsin | Chymotrypsinogen B | *CTRB1* | 0.078 | DB06692 | Aprotinin | NA | MDNA |
| sE-Selectin | E-selectin | *SELE* | 0.023 | DB01136 | Carvedilol | inhibitor | -6.9 |
| P-Selectin | P-Selectin | *SELP* | 0.008 | DB01109 | Heparin | inhibitor | -4.9 |
| | | | | DB08813 | Nadroparin | inhibitor | -4.9 |
| | | | | DB06779 | Dalteparin | inhibitor | -4.9 |
| | | | | DB15271 | Crizanlizumab | inhibitor | 3DSNA |
| VEGF sR2 | Vascular endothelial growth factor receptor 2 | *KDR* | 0.367 | DB06589 | Pazopanib | inhibitor | -6.3 |
| | | | | DB08896 | Regorafenib | inhibitor | -6.5 |
| | | | | DB09079 | Nintedanib | inhibitor | -5.8 |
| | | | | DB14840 | Ripretinib | inhibitor | -6.6 |
| | | | | DB00398 | Sorafenib | antagonist | -6.6 |
| | | | | DB01268 | Sunitinib | inhibitor | -5.6 |
| | | | | DB06595 | Midostaurin | antagonist inhibitor | -5.1 |
| | | | | DB06626 | Axitinib | inhibitor | -6.0 |
| | | | | DB08875 | Cabozantinib | antagonist | **-7.0** |
| | | | | DB08901 | Ponatinib | inhibitor | -6.9 |
| | | | | DB09078 | Lenvatinib | inhibitor | -6.1 |

| | | | | | | | |
|---|---|---|---|---|---|---|---|
| | | | | DB05578 | Ramucirumab | antagonist | 3DSNA |
| | | | | DB12010 | Fostamatinib | inhibitor | -5.3 |
| | | | | DB12147 | Erdafitinib | substrate | -5.5 |
| | | | | DB15822 | Pralsetinib | inhibitor | -6.9 |
| | | | | DB11800 | Tivozanib | inhibitor | -6.4 |
| ADH1B | Alcohol dehydrogenase 1B | *ADH1B* | 0.001 | DB00898 | Ethanol | substrate | -2.8 |
| | | | | DB09462 | Glycerin | NA | -3.7 |
| | | | | DB00157 | NADH | substrate | **-9.6** |
| | | | | DB01213 | Fomepizole | inhibitor | -3.9 |
| Met | Hepatocyte growth factor receptor | *MET* | 0.304 | DB08865 | Crizotinib | inhibitor | **-8.1** |
| | | | | DB08875 | Cabozantinib | antagonist | **-8** |
| | | | | DB12267 | Brigatinib | inhibitor | **-8.2** |
| | | | | DB12010 | Fostamatinib | inhibitor | -6.7 |
| | | | | DB11791 | Capmatinib | inhibitor | **-8.7** |
| | | | | DB15133 | Tepotinib | inhibitor | **-8.3** |
| | | | | DB11800 | Tivozanib | inhibitor | **-8.2** |
| | | | | DB16695 | Amivantamab | antagonist antibody | 3DSNA |
| IGF-I sR | Insulin-like growth factor 1 receptor | *IGF1R* | 0.099 | DB00071 | Insulin pork | NA | MDNA |
| | | | | DB00046 | Insulin lispro | activator | MDNA |
| | | | | DB01307 | Insulin detemir | activator | MDNA |
| | | | | DB00047 | Insulin glargine | activator | MDNA |
| | | | | DB01306 | Insulin aspart | activator | MDNA |
| | | | | DB01309 | Insulin glulisine | activator | MDNA |
| | | | | DB09564 | Insulin degludec | activator | MDNA |

| | | | | | | | |
|---|---|---|---|---|---|---|---|
| | | | | DB14751 | Mecasermin rinfabate | agonist | MDNA |
| | | | | DB09456 | Insulin beef | activator | MDNA |
| | | | | DB08804 | Nandrolone decanoate | inducer | -5.8 |
| | | | | DB01277 | Mecasermin | agonist | 3DSNA |
| | | | | DB00030 | Insulin human | activator | MDNA |
| | | | | DB06343 | Teprotumumab | binder, antibody | 3DSNA |
| | | | | DB12267 | Brigatinib | inhibitor | -5.7 |
| IR | Insulin receptor | *INSR* | 0.013 | DB00047 | Insulin glargine | agonist | MDNA |
| | | | | DB00071 | Insulin pork | binder | MDNA |
| | | | | DB01307 | Insulin detemir | agonist | MDNA |
| | | | | DB00046 | Insulin lispro | agonist | MDNA |
| | | | | DB01306 | Insulin aspart | agonist | MDNA |
| | | | | DB01309 | Insulin glulisine | agonist | MDNA |
| | | | | DB09564 | Insulin degludec | agonist | MDNA |
| | | | | DB09129 | Chromic chloride | activator | MDNA |
| | | | | DB14751 | Mecasermin rinfabate | NA | MDNA |
| | | | | DB09456 | Insulin beef | agonist | MDNA |
| | | | | DB00030 | Insulin human | agonist | MDNA |
| | | | | DB01277 | Mecasermin | NA | 3DSNA |
| | | | | DB12267 | Brigatinib | binding | **-8.4** |
| | | | | DB12010 | Fostamatinib | inhibitor | **-7.5** |

* a score of ≤-7 represents a good interaction between the protein and corresponding drug agent and is bolded.

MDNA: Molecular docking not applicable

3DSNA: 3D structure not available.

Figure 1

Click here to access/download;Figure;Figure 1.pdf

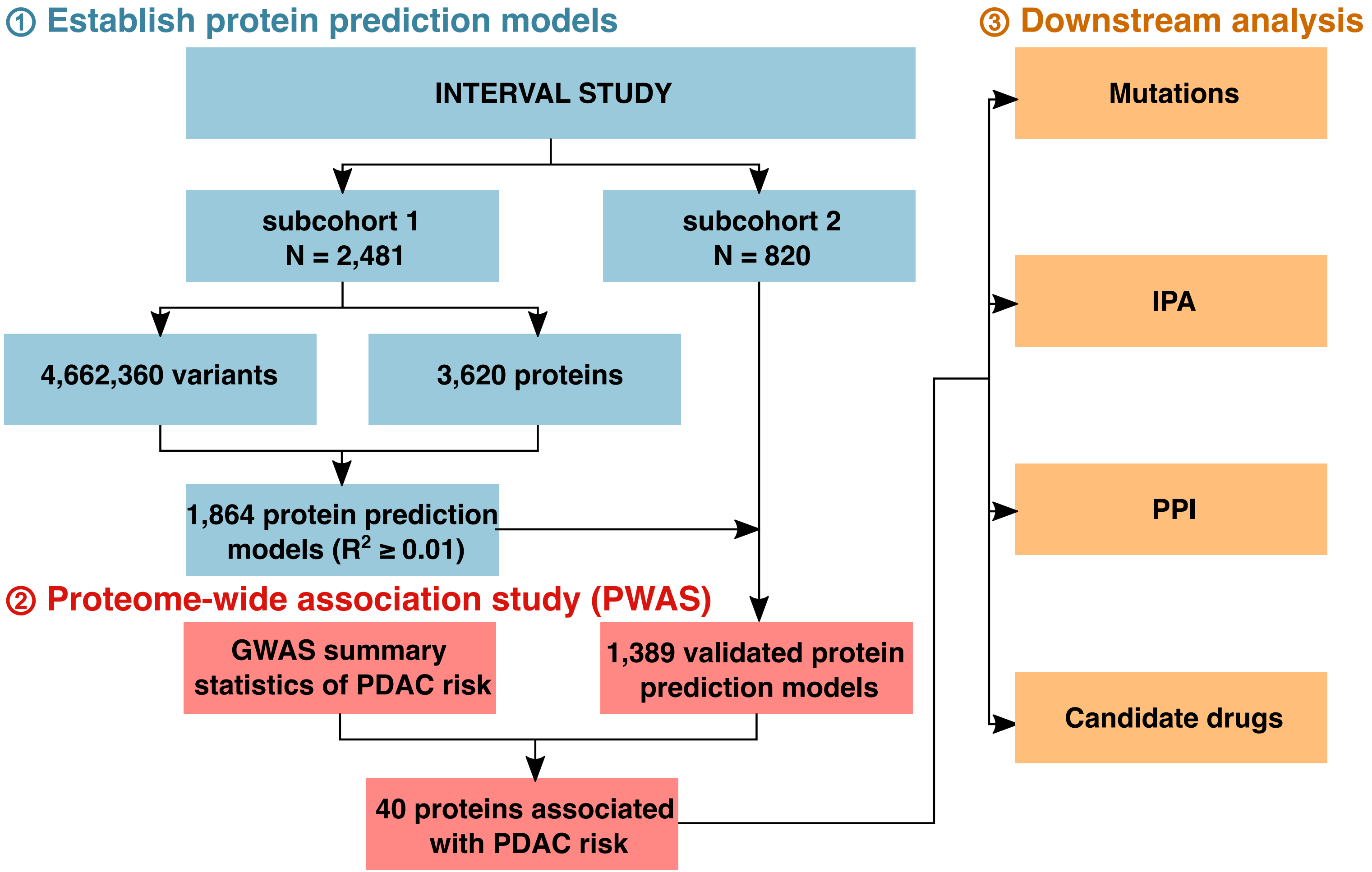

Figure 2

Click here to access/download;Figure;Figure 2.pdf

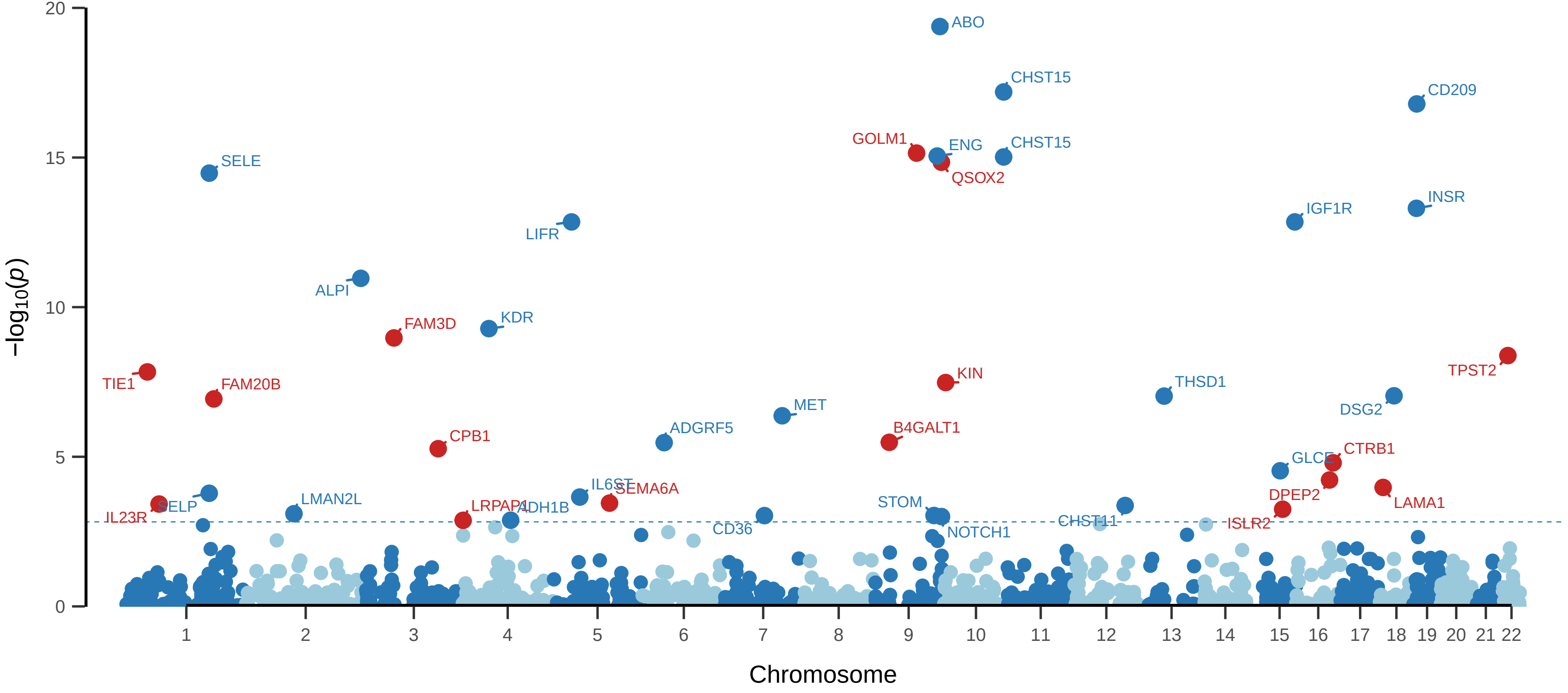

Figure 3

Click here to access/download;Figure;Figure 3.pdf

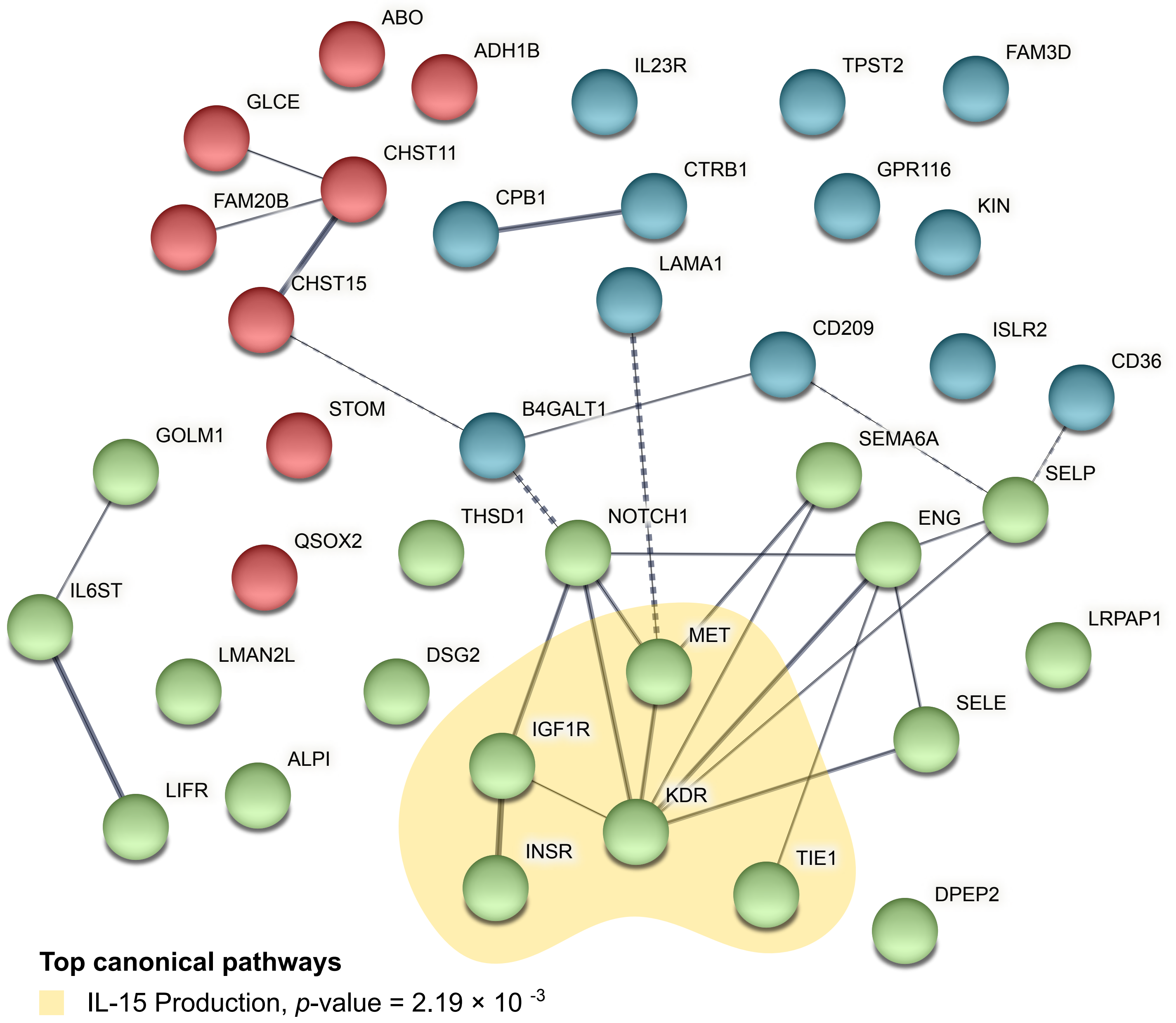

Figure 4

Click here to access/download;Figure;Fig 4.pptx

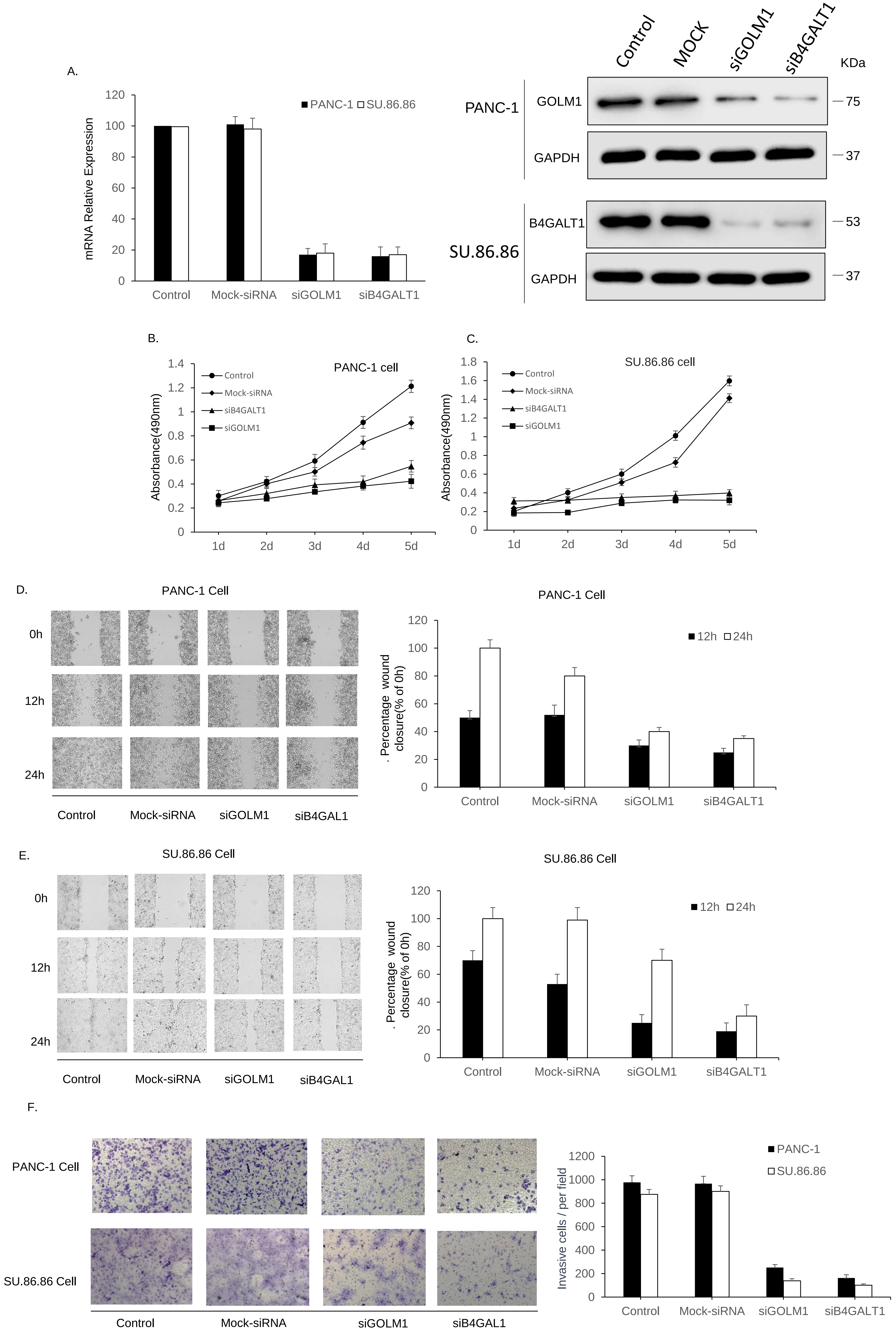

Click here to access/download
**Supplementary Material**
2024-Jan-17 Supplementary File.docx

Personal Cover Click here to access/download;Personal Cover;Cover Letter.docx

UNIVERSITY OF HAWAIʻI
CANCER CENTER

January 17, 2024

Dr. Scott Edmunds

Editor-in-Chief, *GigaScience*

**Proteome-wide association study and functional validation identify novel protein markers for pancreatic ductal adenocarcinoma**

Dear Dr. Edmunds:

Thank you very much for your email sharing with us the reviewers' comments for our earlier submitted manuscript (GIGA-D-23-00321). We are excited to hear that all reviewers think that our work is interesting, unique, and original. We are glad to learn that all reviewers' comments are addressable. We have now carefully addressed all raised concerns and substantially improved our paper. We prepared the point-by-point responses to the reviewer's comments, and tracked changes in the revised manuscript.

We hope that we have satisfactorily addressed all of the reviewers' comments and made this manuscript acceptable for publication in *GigaScience*.

We look forward to hearing from you regarding the decision of *GigaScience* about this manuscript.

Sincerely,

Lang Wu, Ph.D.
Associate Professor
Cancer Epidemiology Division, Population Sciences in the Pacific Program
University of Hawaii Cancer Center, University of Hawaii at Manoa
Email: lwu@cc.hawaii.edu
