# Supplementary material for: Proteome-wide association study and functional validation identify novel protein markers for pancreatic ductal adenocarcinoma
Source: Gigascience. 2024 Apr 12;13:giae012. doi: 10.1093/gigascience/giae012 (PMC11010651; doi:10.1093/gigascience/giae012)
Supplement: giae012_Supplemental_File [file giae012_supplemental_file.docx]

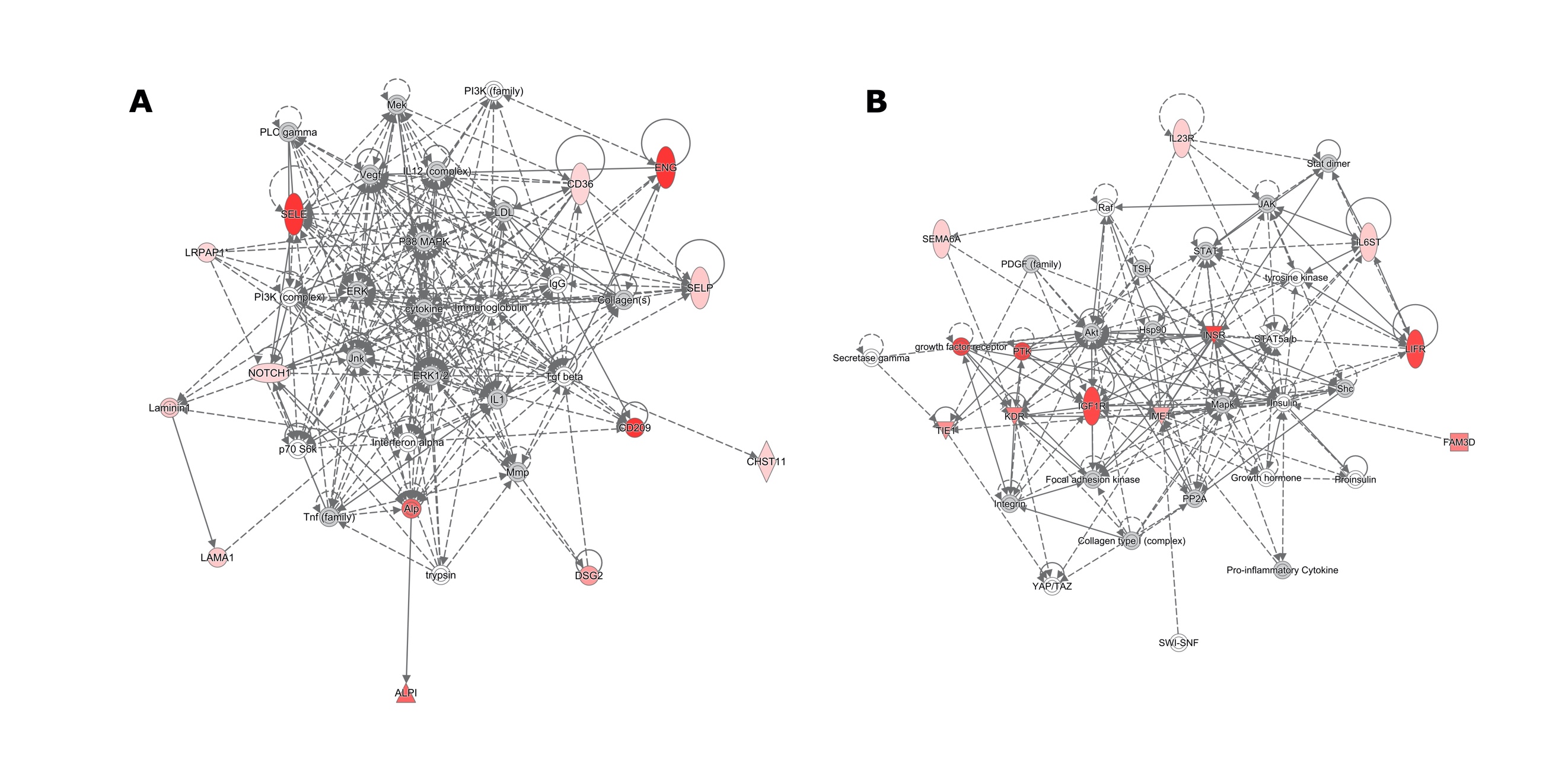


**Supplementary Figure 1.** The top networks identified by IPA. (A) network 1, (B) network 2. The nodes marked with red indicate proteins associated with pancreatic cancer risk. A solid line represents a direct interaction between two nodes and a dotted line indicates an indirect interaction.


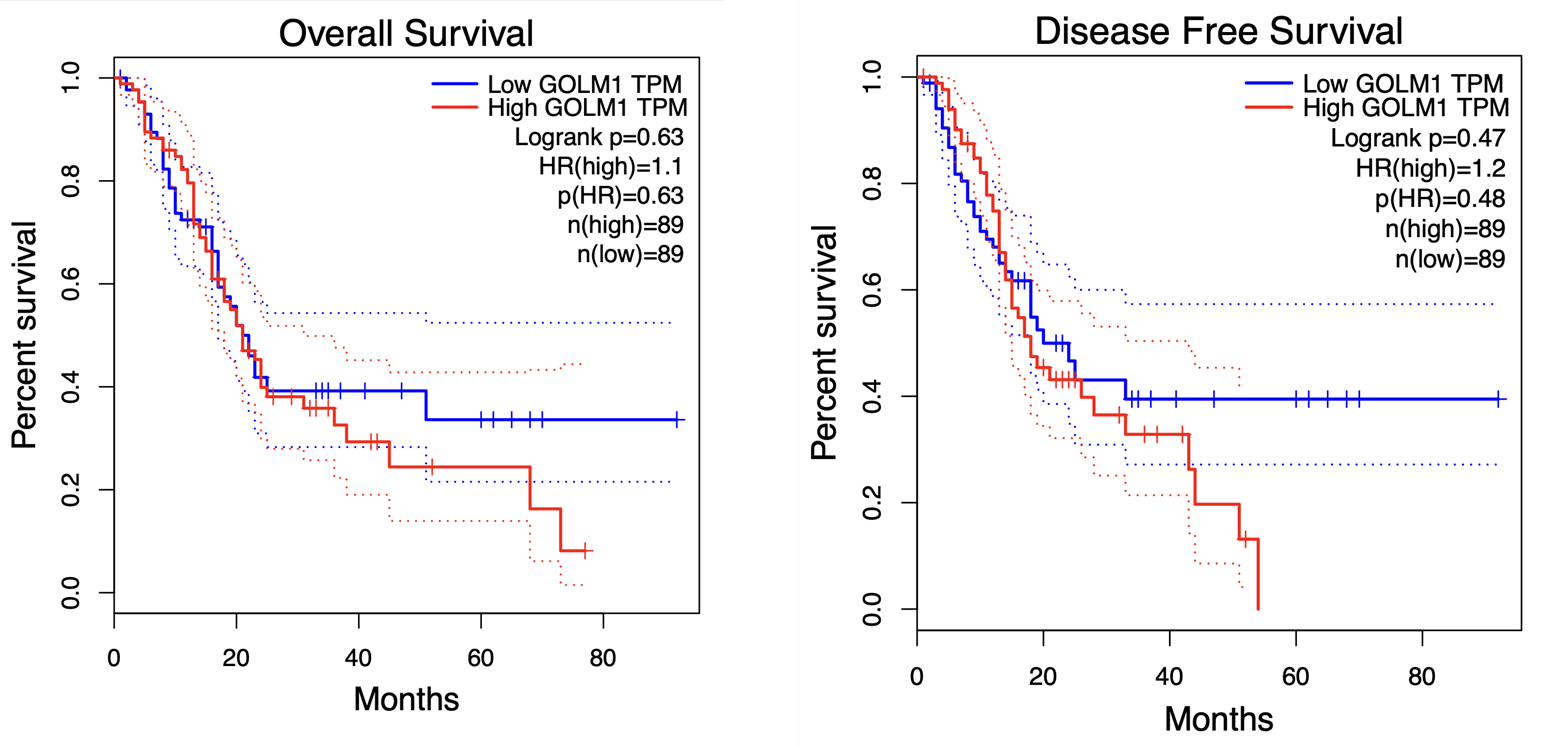

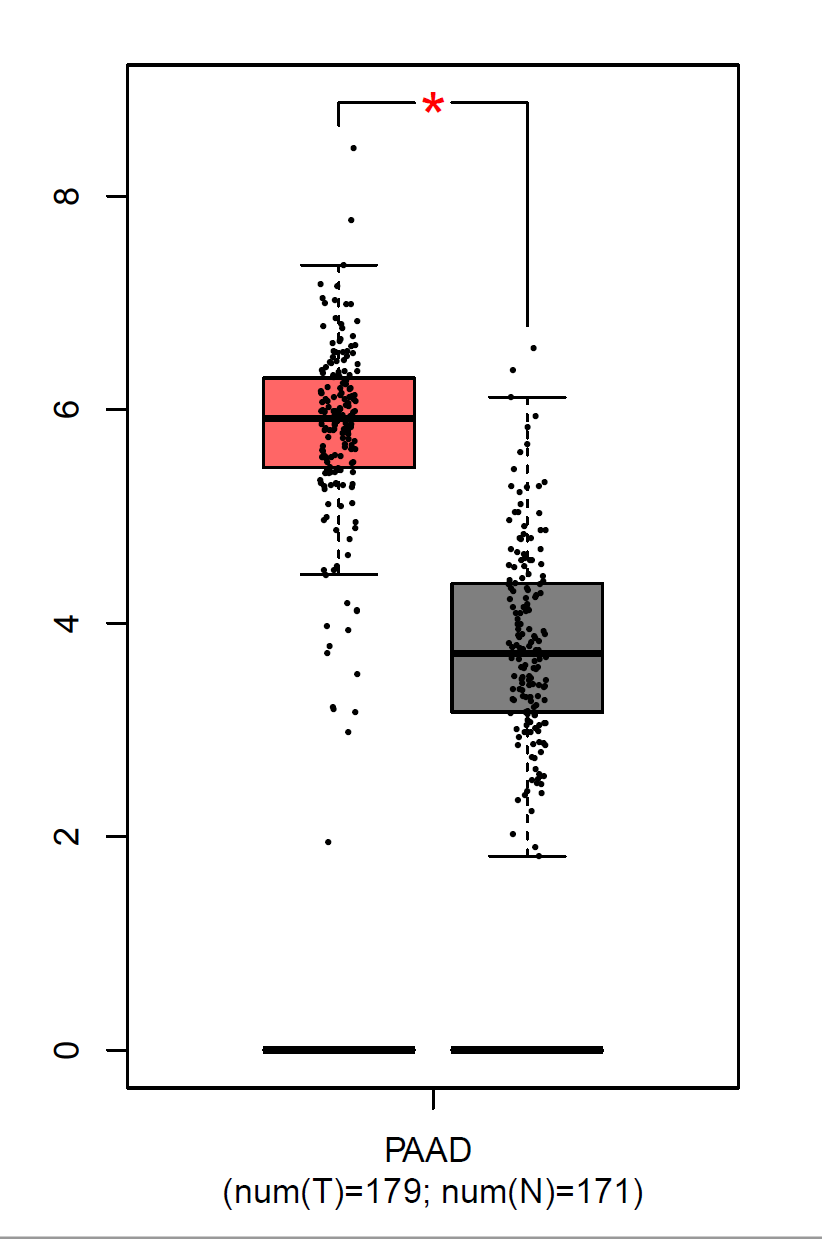


**A B C**

**Supplementary Figure 2:** A.) Box plot analysis of *GOLM1* gene expression in PADD cancer using GEPIA. The plot compares expression levels between tumor (n=179) and normal control (n=171) samples. The analysis was conducted based on RNA sequencing data from the TCGA and GTEx projects. (* *P*<0.01). B.C) Survival analysis of *GOLM1* gene expression in PADD cancer using GEPIA. The Survival Plot compares the overall survival (OS)(B.) and disease free survival(DFS)(C.) between tumor samples with high *GOLM1* expression (n=89) and low *GOLM1* expression (n=89). The analysis utilized the Log-rank test for hypothesis testing, with a significant finding indicating a shorter overall survival and disease free survival in the high *GOLM1* expression group compared with the low *GOLM1* expression group. Cohort thresholds and expression cutoffs were set based on user-defined parameters in the GEPIA platform.


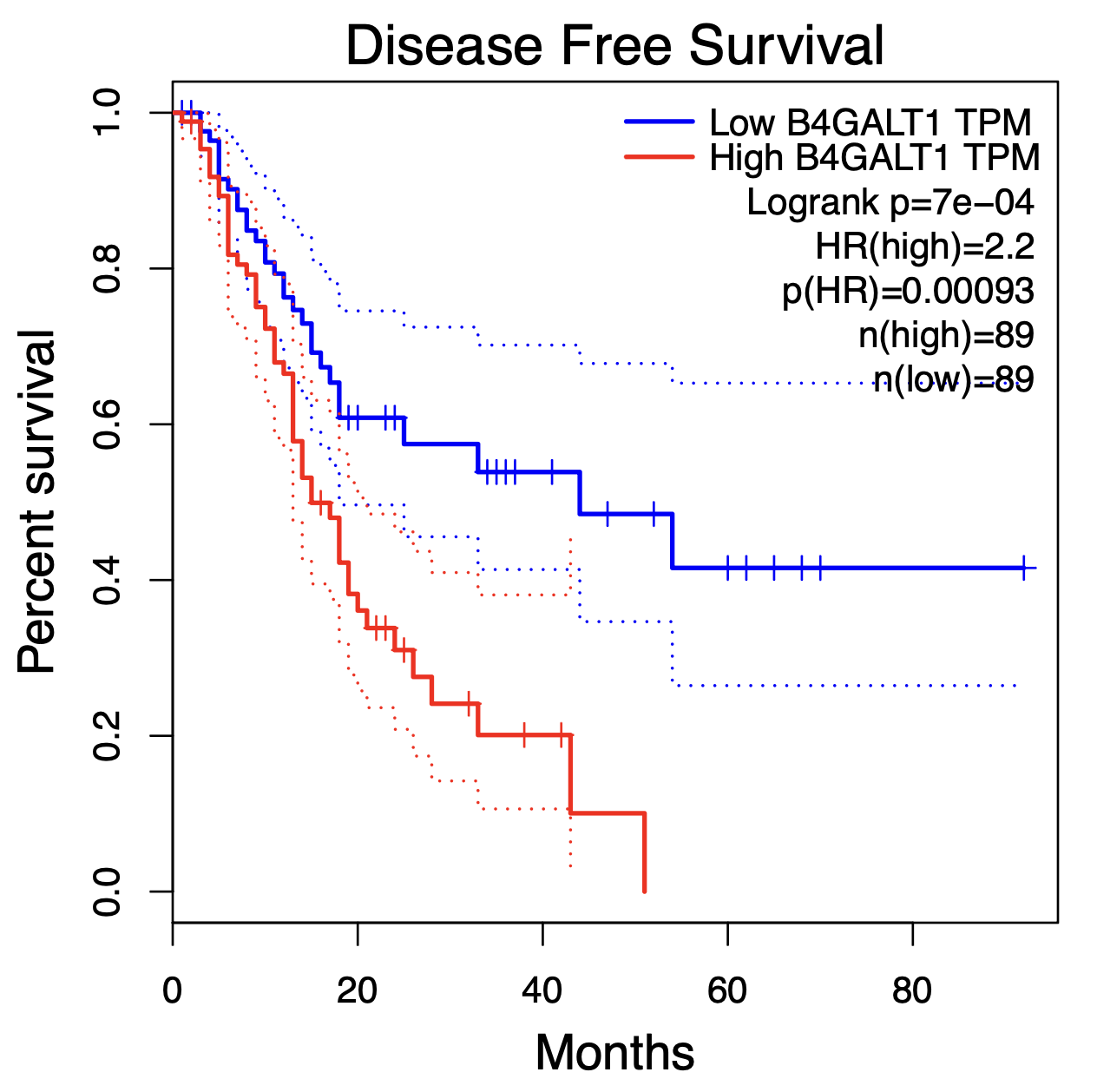

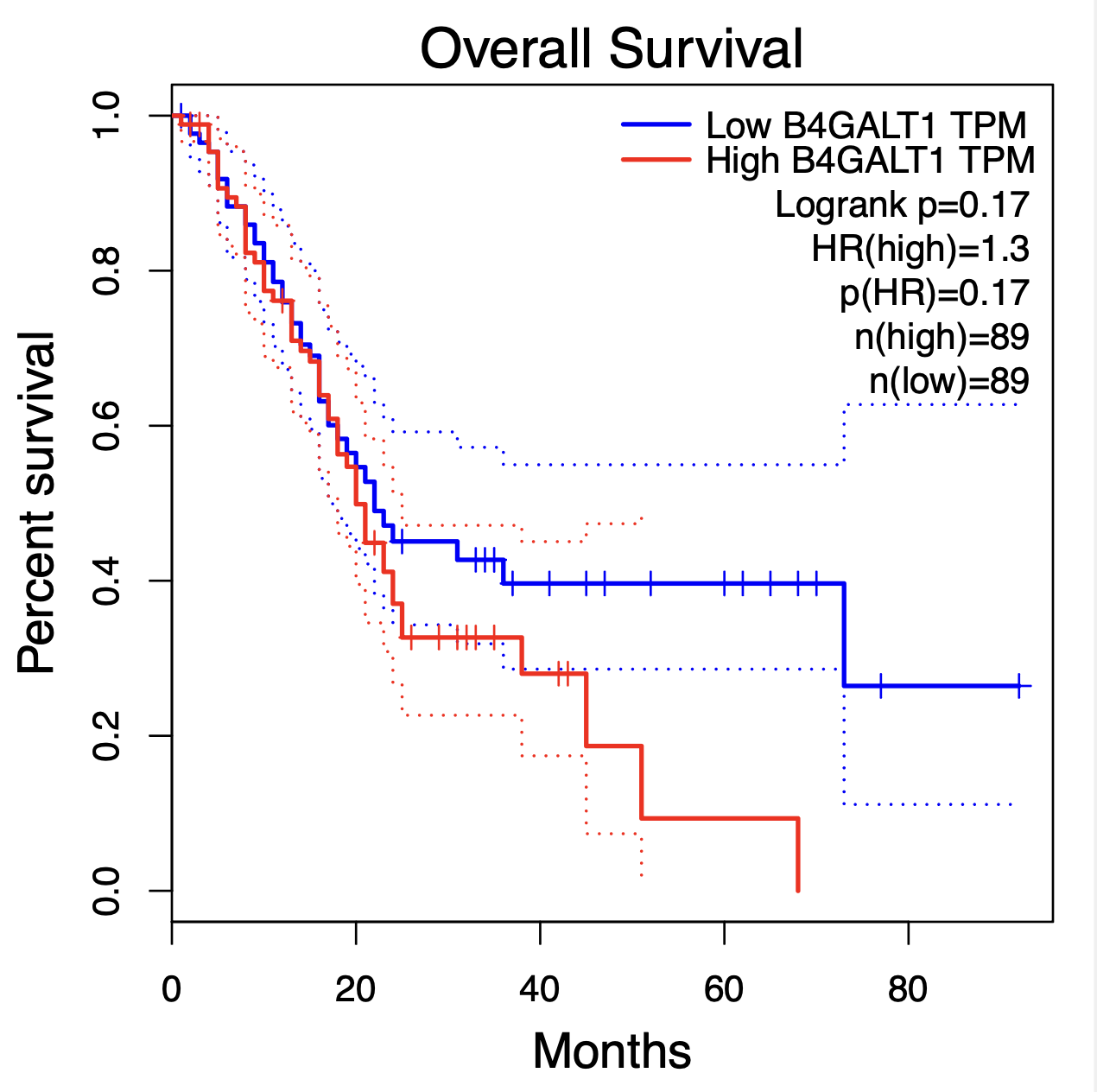

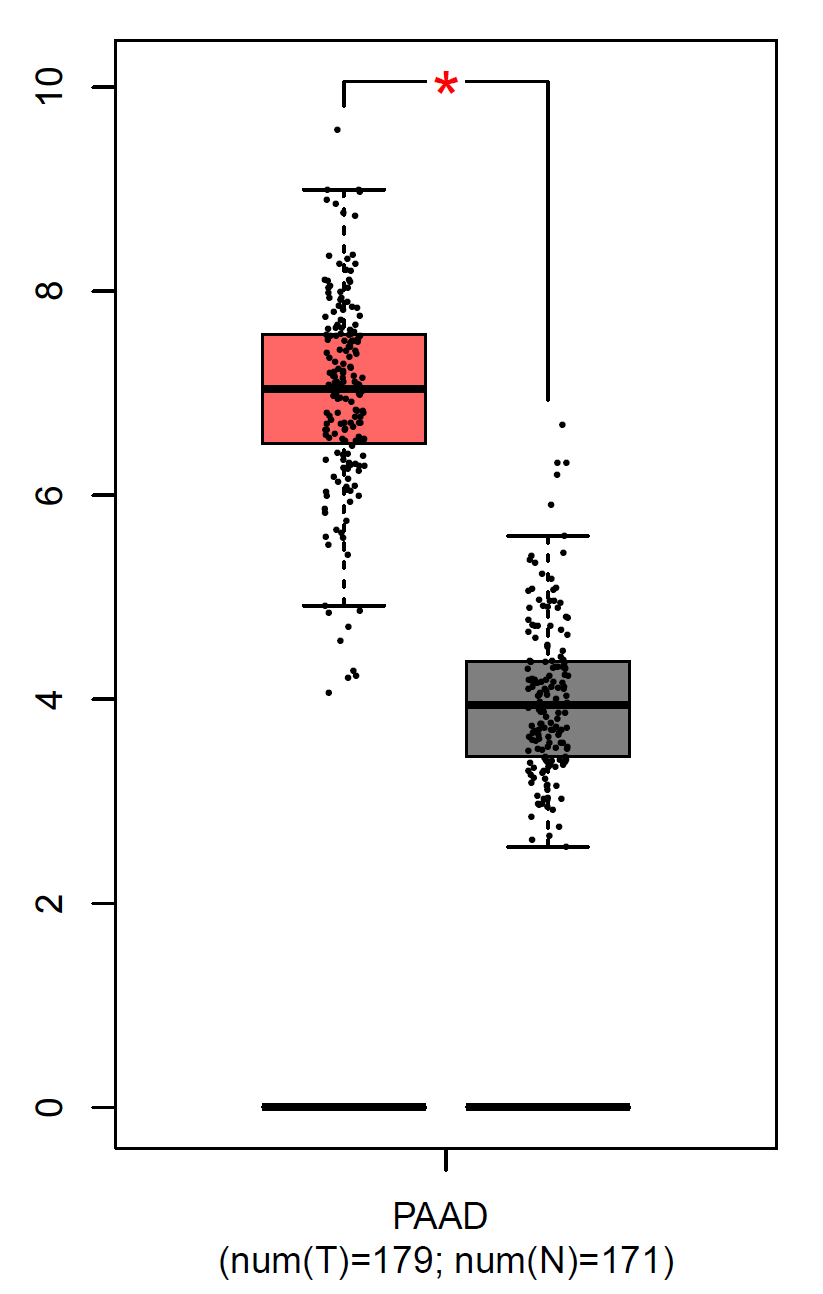


**A B C**

**Supplementary Figure 3:** A.) Box plot analysis of *B4GALT1* gene expression in PADD cancer using GEPIA. The plot compares expression levels between tumor (n=179) and normal control (n=171) samples. The analysis was conducted based on RNA sequencing data from the TCGA and GTEx projects. (* *P*<0.01). B.C) Survival analysis of *B4GALT1* gene expression in PADD cancer using GEPIA. The Survival Plot compares the overall survival (OS)(A.) and the disease free survival(DFS)(B.) between tumor samples with high *B4GALT1* expression (n=89) and low *B4GALT1* expression (n=89). The analysis utilized the Log-rank test for hypothesis testing, with a significant finding indicating a shorter overall survival and disease free survival in the high *B4GALT1* expression group compared to the low *B4GALT1* expression group. Cohort thresholds and expression cutoffs were set based on user-defined parameters in the GEPIA platform.

**Supplementary Table 1**. Associations of proteins identified using pQTL as instruments but not show a significant association with pancreatic cancer risk in the current study

| **Protein** | **Protein full name** | **Protein-encoding gene** | **Prediction model method** | **Number of Prediction SNPs** | **Model internal cross validation R^2^** | **Z-value^a^** | **P-value^a^** | **FDR P-value^b^** | **Association direction in previous study using pQTL as instruments (1)** |
| --- | --- | --- | --- | --- | --- | --- | --- | --- | --- |
| TM11D | Transmembrane protease serine 11D | *TMPRSS11D* | elastic net | 23 | 0.12 | 3.05 | 2.27×10^-3^ | 0.07 | + |
| TENC1 | Tensin-2 | *TNS2* | top1 | 1 | 0.06 | 3.12 | 1.78×10^-3^ | 0.06 | + |
| DOCK9 | Dedicator of cytokinesis protein 9 | *DOCK9* | lasso | 5 | 0.04 | 2.88 | 4.04×10^-3^ | 0.12 | + |
| B3GN2 | N-acetyllactosaminide beta-1,3-N-acetylglucosaminyltransferase 2 | *B3GNT2* | elastic net | 60 | 0.13 | 2.74 | 6.20×10^-3^ | 0.16 | + |
| TLL1 | Tolloid-like protein 1 | *TLL1* | elastic net | 23 | 0.04 | 0.50 | 0.62 | 0.98 | + |
| GFRAL | GDNF family receptor alpha-like | *GFRAL* | elastic net | 68 | 0.05 | 2.94 | 3.30×10^-3^ | 0.10 | + |
| sTie-2 | Angiopoietin-1 receptor, soluble | *TEK* | elastic net | 42 | 0.06 | -0.55 | 0.58 | 0.98 | + |
| F177A | Protein FAM177A1 | *FAM177A1* | lasso | 12 | 0.21 | -2.18 | 2.91×10^-2^ | 0.45 | - |
| Cadherin-5 | Cadherin-5 | *CDH5* | lasso | 11 | 0.18 | 2.55 | 0.01 | 0.26 | + |
| JAG1 | Protein jagged-1 | *JAG1* | best linear unbiased prediction | 1221 | 0.04 | -1.41 | 0.16 | 0.93 | - |
| FAM3B | Protein FAM3B | *FAM3B* | elastic net | 35 | 0.12 | 2.11 | 3.45×10^-2^ | 0.47 | + |
| CRBB2 | Beta-crystallin B2 | *CRYBB2* | NA^c^ | | | | | | + |
| IP-10 | C-X-C motif chemokine 10 | *CXCL10* | NA | | | | | | - |
| C1GLC | C1GALT1-specific chaperone 1 | *C1GALT1C1* | NA | | | | | | + |
| IL-3Ra | Interleukin-3 receptor subunit alpha | *IL3RA* | NA | | | | | | - |

a Associations between genetically predicted protein levels and PDAC risk after adjustment for age, sex, and top 10 principle components

b FDR *P*-value: false discovery rate (FDR) adjusted *P*-value; associations with a FDR *p*≤0.05 considered statistically significant

c NA indicates that no satisfied prediction model was established for the corresponding protein

**Supplementary Table 2**. Comparison of heritability between *cis*+*trans* models and *cis*-only models.

| **ID** | **cis+trans h^2^** | **cis h^2^** |
| --- | --- | --- |
| IDO1.9759.13.3 | 0.19 | 0.02 |
| SERPINA4.3449.58.2 | 0.23 | 0.23 |
| OBP2B.5680.54.3 | 0.39 | 0.05 |
| IL22RA2.9456.34.3 | 0.06 | 0.05 |
| GPNMB.8606.39.3 | 0.09 | 0.05 |
| ICAM5.8245.27.3 | 0.43 | 0.41 |
| LILRA4.8299.66.3 | 0.10 | 0.06 |
| MPO.2580.83.2 | 0.09 | 0.09 |
| PLXNC1.4564.2.2 | 0.21 | 0.21 |
| PRTN3.3514.49.2 | 0.24 | 0.24 |
| ST3GAL6.6947.4.3 | 0.39 | 0.39 |
| PYGL.11441.11.3 | 0.09 | 0.01 |
| PLA2G12B.9380.2.3 | 0.08 | 0.03 |
| CA6.3352.80.3 | 0.60 | 0.60 |
| COL18A1.2201.17.6 | 0.06 | 0.06 |
| DHX58.14012.17.3 | 0.01 | 0.00 |
| LILRA5.7787.25.3 | 0.22 | 0.17 |
| LEPR.5400.52.3 | 0.31 | 0.31 |
| MET.2837.3.2 | 0.09 | 0.02 |
| OLA1.12659.13.3 | 0.03 | 0.01 |
| GABARAPL2.12494.99.3 | 0.05 | 0.02 |
| CCL14.2900.53.3 | 0.26 | 0.26 |
| IGFLR1.7244.16.3 | 0.15 | 0.15 |
| FJX1.7921.65.3 | 0.07 | 0.05 |
| ART3.7970.315.3 | 0.07 | 0.06 |
| CPZ.6493.9.3 | 0.04 | 0.04 |
| CHST15.4469.78.2 | 0.05 | 0.01 |
| ITIH1.7955.195.3 | 0.24 | 0.15 |
| PIANP.9599.6.3 | 0.05 | 0.05 |
| HSP90B1.6393.63.3 | 0.39 | 0.39 |
| C1QC.14100.63.3 | 0.35 | 0.33 |
| PGLYRP1.3329.14.2 | 0.03 | 0.03 |
| CTSF.9212.22.3 | 0.06 | 0.02 |
| CHGA.8476.11.3 | 0.03 | 0.01 |
| TXNRD1.13967.14.3 | 0.13 | 0.01 |
| CLEC11A.2966.65.2 | 0.16 | 0.16 |
| MAN2B2.9251.28.3 | 0.09 | 0.07 |
| CCBL2.12682.5.3 | 0.05 | 0.01 |
| SEMA3G.5628.21.3 | 0.05 | 0.03 |
| ADRBK1.3347.9.2 | 0.20 | 0.00 |
| AMICA1.8232.90.3 | 0.35 | 0.35 |
| CD274.5060.62.3 | 0.02 | 0.01 |
| ISLR2.13124.20.3 | 0.17 | 0.04 |
| GPC1.8697.38.3 | 0.05 | 0.05 |
| IL11RA.3814.63.1 | 0.03 | 0.03 |
| LRIG3.3322.52.2 | 0.03 | 0.03 |
| KLK7.3378.49.2 | 0.19 | 0.16 |
| LILRA5.8766.29.3 | 0.04 | 0.03 |
| FAM3D.13102.1.3 | 0.27 | 0.04 |
| CA10.13666.222.3 | 0.07 | 0.03 |
| IGF2R.3676.15.3 | 0.58 | 0.58 |
| IL6ST.2620.4.2 | 0.08 | 0.03 |
| GALP.9398.30.3 | 0.01 | 0.01 |
| HDHD2.13472.35.3 | 0.14 | 0.05 |
| HTATIP2.10630.5.3 | 0.04 | 0.01 |
| PEAR1.8275.31.3 | 0.11 | 0.09 |
| NMRAL1.13988.67.3 | 0.13 | 0.16 |
| TMEM132C.7173.141.3 | 0.07 | 0.04 |
| MANBA.6382.17.3 | 0.11 | 0.08 |
| STX8.10903.50.3 | 0.08 | 0.02 |
| IMPAD1.9231.23.3 | 0.12 | 0.12 |
| ART3.10970.3.3 | 0.06 | 0.06 |
| CPNE1.5346.24.3 | 0.43 | 0.27 |
| APBB2.12761.12.3 | 0.04 | 0.01 |
| IL5RA.4491.4.2 | 0.36 | 0.36 |
| SERPINF1.7735.17.3 | 0.10 | 0.10 |
| NTNG1.5637.81.3 | 0.09 | 0.09 |
| PCSK1.13388.57.3 | 0.38 | 0.38 |
| SAA1.4336.2.1 | 0.71 | 0.71 |
| PLA2R1.10916.44.3 | 0.47 | 0.47 |
| CECR1.6077.63.3 | 0.62 | 0.62 |
| IL1R2.14133.93.3 | 0.12 | 0.10 |
| ALDH3A1.11480.1.3 | 0.04 | 0.04 |
| GSTP1.4911.49.2 | 0.20 | 0.01 |
| TDGF1.5810.25.3 | 0.73 | 0.68 |
| FLRT2.13122.19.3 | 0.04 | 0.04 |
| LILRB5.7015.8.3 | 0.74 | 0.74 |
| PSG4.5649.83.3 | 0.32 | 0.32 |
| LYZ.4920.10.1 | 0.10 | 0.10 |
| TNXB.5698.60.3 | 0.55 | 0.55 |
| IL7R.5089.11.3 | 0.07 | 0.04 |
| TPSB2.3403.1.2 | 0.70 | 0.70 |
| ADAMTS13.3175.51.5 | 0.15 | 0.15 |
| SCG3.7957.2.3 | 0.12 | 0.12 |
| VPS24.12508.9.3 | 0.15 | 0.00 |
| B4GALT1.13381.49.3 | 0.10 | 0.04 |
| FAM171B.8851.42.3 | 0.04 | 0.04 |
| ICAM1.4342.10.3 | 0.71 | 0.68 |
| SLAMF7.5487.7.3 | 0.42 | 0.42 |
| RGMB.3331.8.1 | 0.03 | 0.02 |
| NCAM2.6507.16.3 | 0.10 | 0.08 |
| SPINK2.13405.61.3 | 0.10 | 0.10 |
| IL17RB.5084.154.3 | 0.11 | 0.10 |
| RNASE2.8394.56.3 | 0.04 | 0.04 |
| SEMA4D.5737.61.3 | 0.28 | 0.28 |
| DNAJC30.7866.11.3 | 0.06 | 0.06 |
| CBL.12016.60.3 | 0.18 | 0.01 |
| SVEP1.11109.56.3 | 0.20 | 0.16 |
| SPINT2.2843.13.2 | 0.45 | 0.45 |
| LRPAP1.3640.14.3 | 0.47 | 0.13 |
| GRN.4992.49.1 | 0.40 | 0.06 |
| CRHBP.6039.24.3 | 0.17 | 0.17 |
| IGDCC4.9793.145.3 | 0.06 | 0.06 |
| ERAP2.8960.3.3 | 0.43 | 0.43 |
| HPGDS.12549.33.3 | 0.10 | 0.10 |
| ADAM23.7049.2.3 | 0.30 | 0.30 |
| REG4.11102.22.3 | 0.03 | 0.03 |
| PDCD1LG2.3004.67.2 | 0.16 | 0.12 |
| PPIL1.9884.8.3 | 0.08 | 0.03 |
| PENK.9076.25.3 | 0.12 | 0.12 |
| CXCL16.2436.49.4 | 0.08 | 0.03 |
| TAC1.9337.43.3 | 0.04 | 0.02 |
| TXNDC5.11212.7.3 | 0.06 | 0.02 |
| POFUT1.5634.39.3 | 0.32 | 0.27 |
| SAT2.12524.18.3 | 0.04 | 0.02 |
| ADGRF5.6409.57.3 | 0.42 | 0.22 |
| CASP3.3593.72.3 | 0.06 | 0.01 |
| LAMA1.LAMB1.LAMC1.2728.62.2 | 0.09 | 0.04 |
| GRAMD1C.8842.16.3 | 0.33 | 0.32 |
| PTHLH.2962.50.2 | 0.09 | 0.02 |
| B4GALT6.10832.24.3 | 0.23 | 0.23 |
| IL27RA.5132.71.3 | 0.64 | 0.60 |
| GLRX2.12486.8.3 | 0.11 | 0.02 |
| CD209.3029.52.2 | 0.30 | 0.09 |
| HLA.DQA2.7757.5.3 | 0.12 | 0.11 |
| GRAMD1C.8336.267.3 | 0.18 | 0.15 |
| PRCP.5722.78.3 | 0.06 | 0.03 |
| CCDC126.6388.21.3 | 0.08 | 0.05 |
| VTN.13125.45.3 | 0.48 | 0.48 |
| MXRA7.8005.1.3 | 0.03 | 0.03 |
| PTN.3045.72.2 | 0.03 | 0.03 |
| CHI3L2.9383.24.3 | 0.02 | 0.01 |
| DUSP13.6525.17.3 | 0.09 | 0.04 |
| PDGFRB.3459.49.2 | 0.48 | 0.48 |
| CLPS.5749.53.3 | 0.31 | 0.31 |
| MAN1A2.9077.10.3 | 0.03 | 0.03 |
| CXCL5.2979.8.2 | 0.01 | 0.00 |
| CDH5.2819.23.2 | 0.16 | 0.01 |
| SIGLEC9.3007.7.2 | 0.76 | 0.76 |
| CHST9.11646.4.3 | 0.05 | 0.05 |
| CBR3.14091.42.3 | 0.19 | 0.19 |
| CD59.11514.196.3 | 0.06 | 0.06 |
| GP1BA.4990.87.1 | 0.04 | 0.01 |
| TAPBP.12378.71.3 | 0.20 | 0.20 |
| ITIH5.8233.2.3 | 0.16 | 0.16 |
| IGLL1.6485.59.3 | 0.08 | 0.06 |
| ICOSLG.5061.27.3 | 0.08 | 0.02 |
| SYT11.7089.42.3 | 0.01 | 0.00 |
| RELT.14112.40.3 | 0.03 | 0.01 |
| NELL1.6544.33.3 | 0.18 | 0.14 |
| BST1.4535.50.2 | 0.49 | 0.50 |
| PAM.5620.13.3 | 0.24 | 0.27 |
| SERPINA4.14105.5.3 | 0.16 | 0.16 |
| NAGK.3894.15.2 | 0.19 | 0.06 |
| ERAP1.4964.67.1 | 0.43 | 0.43 |
| COL1A1.11140.56.3 | 0.07 | 0.02 |
| FAM20B.7198.197.3 | 0.05 | 0.02 |
| SERPINA12.6551.94.3 | 0.11 | 0.08 |
| FSTL4.9350.3.3 | 0.05 | 0.03 |
| CST6.3303.23.2 | 0.02 | 0.02 |
| NRXN3.5111.15.3 | 0.02 | 0.02 |
| MAP2K3.6151.18.3 | 0.09 | 0.01 |
| FCN1.3613.62.5 | 0.17 | 0.17 |
| TLR4.LY96.3647.49.4 | 0.14 | 0.12 |
| SOD3.8463.2.3 | 0.46 | 0.53 |
| SIGLEC12.10037.98.3 | 0.31 | 0.31 |
| FSTL1.13112.179.3 | 0.01 | 0.01 |
| APLP2.10627.87.3 | 0.04 | 0.02 |
| CBLN4.5688.65.3 | 0.07 | 0.05 |
| SERPINA10.13119.26.3 | 0.37 | 0.13 |
| IL12B.IL23A.10365.132.3 | 0.06 | 0.05 |
| RETN.3046.31.1 | 0.12 | 0.12 |
| CAT.3488.64.2 | 0.03 | 0.03 |
| FAM3B.9177.6.3 | 0.26 | 0.22 |
| F10.4878.3.1 | 0.06 | 0.06 |
| OSMR.10892.8.3 | 0.06 | 0.06 |
| GPNMB.8289.8.3 | 0.03 | 0.03 |
| TIMP4.6462.12.3 | 0.05 | 0.05 |
| PRTN3.13720.95.3 | 0.24 | 0.24 |
| RNASE4.5644.60.3 | 0.15 | 0.12 |
| CBLN1.9313.27.3 | 0.49 | 0.49 |
| ESAM.2981.9.3 | 0.05 | 0.04 |
| SPATA20.11117.2.3 | 0.04 | 0.03 |
| CDON.4541.49.2 | 0.17 | 0.17 |
| ADAMTS5.3168.8.2 | 0.15 | 0.15 |
| CD109.3290.50.2 | 0.10 | 0.10 |
| IL12RB2.3815.14.1 | 0.02 | 0.02 |
| N6AMT1.11096.57.3 | 0.07 | 0.00 |
| CEL.9796.4.3 | 0.05 | 0.03 |
| TMEM2.8992.1.3 | 0.06 | 0.01 |
| DSG2.9484.75.3 | 0.06 | 0.03 |
| CST7.3302.58.1 | 0.57 | 0.60 |
| CXCL11.3038.9.2 | 0.04 | 0.03 |
| NAGPA.11208.15.3 | 0.11 | 0.11 |
| POMGNT2.6359.50.3 | 0.04 | 0.03 |
| GHR.2948.58.2 | 0.05 | 0.05 |
| LIPN.8097.77.3 | 0.52 | 0.52 |
| AMY1A.7918.114.3 | 0.53 | 0.53 |
| FCGR2B.3310.62.1 | 0.79 | 0.79 |
| ASIP.5676.54.3 | 0.14 | 0.21 |
| RARRES1.8398.277.3 | 0.46 | 0.47 |
| JAG1.5092.51.3 | 0.06 | 0.01 |
| IFI16.12893.159.3 | 0.32 | 0.07 |
| CD33.3166.92.1 | 0.55 | 0.55 |
| FAS.5392.73.2 | 0.08 | 0.08 |
| SFRP1.3221.54.1 | 0.04 | 0.04 |
| B3GAT3.6897.38.3 | 0.10 | 0.08 |
| CCL23.3028.36.2 | 0.27 | 0.27 |
| GZMK.9545.156.3 | 0.02 | 0.02 |
| FLT4.2358.19.2 | 0.43 | 0.03 |
| UCMA.10977.55.3 | 0.22 | 0.22 |
| IL15RA.14054.17.3 | 0.36 | 0.36 |
| NRBP1.12616.45.3 | 0.14 | 0.01 |
| FGF7.14031.18.3 | 0.06 | 0.05 |
| NQO1.9837.60.3 | 0.17 | 0.17 |
| DKK1.3535.84.1 | 0.06 | 0.02 |
| DPT.4979.34.2 | 0.16 | 0.16 |
| KLK11.2831.29.1 | 0.34 | 0.34 |
| TCN1.11232.46.3 | 0.11 | 0.15 |
| MANSC4.9578.263.3 | 0.09 | 0.09 |
| OAS1.10361.25.3 | 0.07 | 0.02 |
| CPM.7768.10.3 | 0.03 | 0.03 |
| IL1RL2.2994.71.2 | 0.05 | 0.05 |
| NTN4.3327.27.1 | 0.03 | 0.03 |
| ARL3.12571.14.3 | 0.24 | 0.02 |
| SIGLEC14.5125.6.3 | 0.14 | 0.05 |
| MATN2.3325.2.2 | 0.03 | 0.03 |
| DDX58.12382.2.3 | 0.09 | 0.04 |
| PPT1.9244.27.3 | 0.32 | 0.14 |
| NPW.9986.14.3 | 0.17 | 0.18 |
| CPA4.9267.2.3 | 0.69 | 0.69 |
| RMDN1.7096.30.3 | 0.35 | 0.06 |
| VEGFC.3132.1.1 | 0.28 | 0.34 |
| AKR1C1.12618.50.3 | 0.05 | 0.05 |
| CXCL1.2985.35.1 | 0.29 | 0.29 |
| PSG3.6444.15.3 | 0.27 | 0.27 |
| GZMA.3440.7.2 | 0.05 | 0.02 |
| CCL7.4886.3.1 | 0.10 | 0.10 |
| ALCAM.5451.1.3 | 0.05 | 0.05 |
| ESM1.3805.16.2 | 0.04 | 0.04 |
| PLCG1.4563.61.2 | 0.06 | 0.01 |
| XXYLT1.6375.75.3 | 0.05 | 0.05 |
| COL15A1.8974.172.3 | 0.04 | 0.04 |
| PTGR1.13543.7.3 | 0.12 | 0.12 |
| SPINK6.5731.1.3 | 0.12 | 0.12 |
| LHB.8376.25.4 | 0.02 | 0.02 |
| SEMA5A.13132.14.3 | 0.47 | 0.47 |
| APMAP.10605.22.3 | 0.08 | 0.07 |
| CTRB1.5671.1.3 | 0.35 | 0.36 |
| ENPP7.4435.66.2 | 0.59 | 0.59 |
| EGF.5509.7.3 | 0.03 | 0.03 |
| LILRB1.5090.49.2 | 0.53 | 0.29 |
| CREB3L4.11308.8.3 | 0.15 | 0.02 |
| CHIT1.3600.2.3 | 0.48 | 0.48 |
| S100A6.13090.17.3 | 0.16 | 0.02 |
| SERPING1.4479.14.2 | 0.24 | 0.24 |
| SECTM1.13093.6.3 | 0.09 | 0.05 |
| MINPP1.5586.66.3 | 0.08 | 0.01 |
| AKR1A1.4192.10.2 | 0.46 | 0.29 |
| TNFSF14.5355.69.3 | 0.05 | 0.01 |
| SEMA6A.7945.10.3 | 0.10 | 0.05 |
| GLTPD2.7948.129.3 | 0.08 | 0.08 |
| F10.3077.66.2 | 0.06 | 0.06 |
| MFAP2.9294.45.3 | 0.08 | 0.02 |
| MAPK13.5006.71.1 | 0.24 | 0.04 |
| HP.3054.3.2 | 0.66 | 0.66 |
| UGT1A6.7891.45.3 | 0.05 | 0.05 |
| RGMA.5483.1.3 | 0.09 | 0.03 |
| IL16.2774.10.3 | 0.27 | 0.15 |
| COL11A2.11278.4.3 | 0.12 | 0.12 |
| CCL17.3519.3.2 | 0.10 | 0.06 |
| DEFB1.6629.3.3 | 0.15 | 0.13 |
| EPHB2.5077.28.3 | 0.04 | 0.04 |
| CD48.3292.75.1 | 0.06 | 0.06 |
| NTN1.6649.51.3 | 0.14 | 0.14 |
| CHST15.14097.86.3 | 0.06 | 0.01 |
| MMP9.2579.17.5 | 0.08 | 0.08 |
| CCDC134.5587.3.3 | 0.08 | 0.01 |
| GPNMB.8240.207.3 | 0.02 | 0.02 |
| SPINT3.7926.13.3 | 0.09 | 0.08 |
| ICAM5.5124.62.3 | 0.26 | 0.24 |
| APOF.12370.30.3 | 0.02 | 0.02 |
| KDR.3651.50.5 | 0.29 | 0.25 |
| IL18R1.14079.14.3 | 0.14 | 0.14 |
| FAM151A.7856.51.3 | 0.10 | 0.10 |
| LTF.2795.23.3 | 0.05 | 0.05 |
| RET.3220.40.2 | 0.04 | 0.04 |
| GFRAL.6920.1.3 | 0.09 | 0.08 |
| CRP.4337.49.2 | 0.04 | 0.03 |
| FUT8.8244.16.3 | 0.47 | 0.48 |
| ESAM.7841.84.3 | 0.04 | 0.03 |
| EMILIN3.8773.172.3 | 0.08 | 0.08 |
| CTSH.8465.52.3 | 0.48 | 0.48 |
| RTN4R.5105.2.3 | 0.15 | 0.15 |
| IL17RA.2992.59.2 | 0.72 | 0.72 |
| DNAJA4.9744.139.3 | 0.02 | 0.01 |
| CDNF.4962.52.1 | 0.04 | 0.04 |
| HINT1.5900.11.2 | 0.02 | 0.02 |
| TREM1.9266.1.3 | 0.15 | 0.15 |
| CRISP2.9282.12.3 | 0.51 | 0.51 |
| TNFRSF1B.8368.102.3 | 0.02 | 0.02 |
| GALNT16.8923.94.3 | 0.04 | 0.04 |
| MANSC1.9557.5.3 | 0.09 | 0.07 |
| CFHR5.7885.17.3 | 0.16 | 0.16 |
| APOE.2937.10.2 | 0.15 | 0.15 |
| CCL25.14068.29.3 | 0.10 | 0.08 |
| H6PD.7161.25.3 | 0.57 | 0.24 |
| CNTN4.3298.52.2 | 0.10 | 0.10 |
| TIE1.2844.53.2 | 0.24 | 0.03 |
| MSMB.10620.21.3 | 0.61 | 0.67 |
| CFHR5.3666.17.4 | 0.34 | 0.34 |
| CCL25.2705.5.2 | 0.20 | 0.18 |
| ASAH2.3212.30.3 | 0.30 | 0.30 |
| CCL5.5480.49.3 | 0.03 | 0.01 |
| FAM3B.5618.50.3 | 0.18 | 0.17 |
| GNMT.14006.36.3 | 0.06 | 0.06 |
| C1QTNF5.7810.20.3 | 0.16 | 0.11 |
| RNASE6.5646.20.3 | 0.34 | 0.32 |
| SWAP70.13552.7.3 | 0.32 | 0.06 |
| CST6.14711.27.3 | 0.03 | 0.03 |
| CPXM1.6255.74.3 | 0.16 | 0.14 |
| ARFIP1.13488.3.3 | 0.08 | 0.02 |
| COCH.7227.75.3 | 0.14 | 0.10 |
| S100A12.5852.6.3 | 0.25 | 0.03 |
| PSG5.9314.9.3 | 0.12 | 0.01 |
| IL1RAP.2630.12.2 | 0.47 | 0.47 |
| MANEA.8014.359.3 | 0.48 | 0.48 |
| UNC5C.5139.32.3 | 0.08 | 0.06 |
| FUT3.4548.4.2 | 0.48 | 0.50 |
| CACNA2D3.8885.6.3 | 0.07 | 0.06 |
| NSF.13992.12.3 | 0.03 | 0.00 |
| KLK14.8620.56.3 | 0.26 | 0.26 |
| THSD1.5621.64.3 | 0.07 | 0.06 |
| ENPP5.6556.5.3 | 0.49 | 0.49 |
| APOL1.11510.31.3 | 0.33 | 0.09 |
| SIGLEC7.2742.68.2 | 0.17 | 0.15 |
| CD177.13116.25.3 | 0.87 | 0.86 |
| CCL16.4913.78.1 | 0.60 | 0.61 |
| LPO.4801.13.3 | 0.06 | 0.04 |
| MGAT2.6909.40.3 | 0.02 | 0.02 |
| HS6ST1.5465.32.3 | 0.09 | 0.04 |
| GNLY.3195.50.2 | 0.58 | 0.35 |
| THBS2.3339.33.1 | 0.18 | 0.18 |
| SLITRK3.10565.19.3 | 0.10 | 0.10 |
| EPHB6.5078.82.3 | 0.06 | 0.06 |
| APOE.5312.49.3 | 0.03 | 0.03 |
| DEFB119.8370.102.3 | 0.08 | 0.00 |
| FRZB.13740.51.3 | 0.52 | 0.52 |
| B3GNT2.7980.72.3 | 0.13 | 0.05 |
| DPP7.8346.9.3 | 0.06 | 0.06 |
| FCRL4.8973.23.3 | 0.61 | 0.61 |
| IL18R1.3446.7.2 | 0.18 | 0.18 |
| CLMP.10440.26.3 | 0.06 | 0.06 |
| AGER.4125.52.2 | 0.20 | 0.07 |
| GSTA1.12446.49.3 | 0.06 | 0.06 |
| FCGR3B.3311.27.1 | 0.14 | 0.13 |
| SELP.4154.57.2 | 0.32 | 0.25 |
| XCL1.14078.69.3 | 0.08 | 0.08 |
| CHI3L1.11104.13.3 | 0.77 | 0.77 |
| PDCD5.12517.52.3 | 0.31 | 0.05 |
| MGAT4B.7141.21.3 | 0.08 | 0.08 |
| MAPK3.2855.49.2 | 0.17 | 0.01 |
| SEMA3E.5363.51.3 | 0.42 | 0.42 |
| CRELD1.7628.40.3 | 0.36 | 0.36 |
| TNFAIP6.5036.50.1 | 0.15 | 0.15 |
| PTPN11.3397.7.4 | 0.07 | 0.01 |
| FCGR2A.3309.2.2 | 0.84 | 0.84 |
| FUT5.4549.78.2 | 0.27 | 0.27 |
| FAH.11424.4.3 | 0.06 | 0.05 |
| TAX1BP3.12498.12.3 | 0.05 | 0.02 |
| GXYLT1.8229.1.3 | 0.13 | 0.08 |
| NCR1.8360.169.3 | 0.05 | 0.05 |
| CCL3.3040.59.1 | 0.14 | 0.14 |
| COLEC10.6558.5.3 | 0.26 | 0.01 |
| KDELC2.8296.117.3 | 0.05 | 0.05 |
| ELAVL1.11592.1.3 | 0.13 | 0.01 |
| MFGE8.4455.89.2 | 0.04 | 0.04 |
| COLEC11.4430.44.3 | 0.55 | 0.55 |
| CLEC12A.11187.11.3 | 0.72 | 0.72 |
| RELT.5115.31.3 | 0.04 | 0.04 |
| PMEL.6472.40.3 | 0.02 | 0.02 |
| LCT.9017.58.3 | 0.13 | 0.13 |
| LGALS2.3033.57.1 | 0.07 | 0.07 |
| IL1RAP.14048.7.3 | 0.45 | 0.45 |
| FAM177A1.8039.41.3 | 0.21 | 0.17 |
| SMPDL3A.14086.11.3 | 0.72 | 0.72 |
| MTHFS.14107.1.3 | 0.27 | 0.03 |
| RRM2B.8925.25.3 | 0.11 | 0.02 |
| CCL22.3508.78.3 | 0.07 | 0.04 |
| SIRPA.5430.66.3 | 0.54 | 0.54 |
| SPARCL1.4467.49.2 | 0.13 | 0.13 |
| SMAP1.11649.3.3 | 0.14 | 0.03 |
| NEO1.8900.28.3 | 0.05 | 0.02 |
| SPOCK3.9906.21.3 | 0.17 | 0.16 |
| FCN2.3313.21.2 | 0.19 | 0.16 |
| FCRL3.4440.15.2 | 0.08 | 0.08 |
| CCL15.14109.15.3 | 0.38 | 0.35 |
| ICOSLG.9303.9.3 | 0.19 | 0.19 |
| COLEC12.5457.5.2 | 0.05 | 0.02 |
| NUDT9.9482.110.3 | 0.05 | 0.05 |
| CCL23.2913.1.2 | 0.35 | 0.35 |
| S100A4.14116.129.3 | 0.09 | 0.02 |
| IL6R.8092.29.3 | 0.50 | 0.50 |
| NRP1.3214.3.2 | 0.06 | 0.04 |
| LGALS9.9197.4.3 | 0.03 | 0.01 |
| CPB1.6356.3.3 | 0.07 | 0.03 |
| NME2.4249.64.2 | 0.10 | 0.03 |
| AHSG.3581.53.3 | 0.20 | 0.20 |
| TREML2.5736.1.3 | 0.09 | 0.09 |
| SIGLEC14.8248.222.3 | 0.57 | 0.57 |
| PCOLCE2.6081.52.3 | 0.27 | 0.27 |
| PSAPL1.8814.33.3 | 0.13 | 0.13 |
| TPST1.7928.183.3 | 0.02 | 0.02 |
| BPI.4126.22.1 | 0.22 | 0.22 |
| NCAM1.4498.62.2 | 0.06 | 0.06 |
| CFH.4159.130.1 | 0.11 | 0.11 |
| LILRA6.7059.14.3 | 0.78 | 0.78 |
| SVEP1.11178.21.3 | 0.24 | 0.19 |
| CRISPLD2.5691.2.3 | 0.07 | 0.07 |
| TEK.3773.15.4 | 0.10 | 0.07 |
| CHST11.7779.86.3 | 0.15 | 0.12 |
| ASPN.6451.64.3 | 0.09 | 0.09 |
| ALOX15B.12422.143.3 | 0.12 | 0.04 |
| ABO.9253.52.3 | 0.55 | 0.55 |
| IL6R.4139.71.2 | 0.53 | 0.53 |
| COLGALT1.5638.23.3 | 0.07 | 0.02 |
| NFASC.7179.69.3 | 0.25 | 0.24 |
| ANGPTL1.11142.11.3 | 0.12 | 0.12 |
| ESD.4984.83.1 | 0.24 | 0.24 |
| NQO2.9754.33.3 | 0.37 | 0.37 |
| TMEM132A.7871.16.3 | 0.34 | 0.34 |
| CD300A.5630.48.3 | 0.08 | 0.06 |
| PSG9.9335.28.3 | 0.06 | 0.06 |
| APOB.2797.56.2 | 0.06 | 0.01 |
| MRC1.2637.77.2 | 0.44 | 0.44 |
| GDF15.4374.45.2 | 0.35 | 0.35 |
| NAAA.3173.49.2 | 0.18 | 0.15 |
| ACYP2.12812.25.3 | 0.17 | 0.02 |
| EDAR.2977.7.2 | 0.29 | 0.32 |
| SPINT1.2828.82.2 | 0.08 | 0.02 |
| STX7.8274.64.3 | 0.22 | 0.01 |
| CREG1.9357.4.3 | 0.14 | 0.03 |
| LSAMP.2999.6.2 | 0.02 | 0.02 |
| SERPINA10.6583.67.3 | 0.48 | 0.07 |
| CNTN5.3299.29.2 | 0.17 | 0.06 |
| VEGFA.14032.2.3 | 0.28 | 0.24 |
| DNAJB11.7110.2.3 | 0.05 | 0.01 |
| ENTPD5.4437.56.3 | 0.08 | 0.08 |
| CFB.4129.72.1 | 0.27 | 0.22 |
| POSTN.6645.53.3 | 0.04 | 0.04 |
| NCR3.3003.29.2 | 0.11 | 0.11 |
| MICB.5102.55.3 | 0.21 | 0.20 |
| RBP7.14208.3.3 | 0.23 | 0.00 |
| MBL2.3000.66.1 | 0.80 | 0.79 |
| FCER2.3291.30.2 | 0.09 | 0.09 |
| FAM20A.6433.57.3 | 0.06 | 0.03 |
| SMOC1.13118.5.3 | 0.09 | 0.08 |
| TPST2.8024.64.3 | 0.08 | 0.04 |
| GLCE.7808.5.3 | 0.27 | 0.16 |
| QSOX2.8397.147.3 | 0.31 | 0.09 |
| CCL15.3509.1.1 | 0.35 | 0.33 |
| NUDT12.13947.371.3 | 0.06 | 0.06 |
| POSTN.6650.20.3 | 0.04 | 0.04 |
| C4A.C4B.4481.34.2 | 0.24 | 0.32 |
| CBR1.12381.26.3 | 0.22 | 0.23 |
| TNC.4155.3.2 | 0.42 | 0.30 |
| DDX39B.9742.59.3 | 0.28 | 0.01 |
| MTRF1L.11134.30.3 | 0.02 | 0.02 |
| MMP10.8479.4.3 | 0.11 | 0.09 |
| QDPR.11257.1.3 | 0.24 | 0.24 |
| PDGFD.9341.1.3 | 0.09 | 0.06 |
| CD163.5028.59.1 | 0.11 | 0.01 |
| GFRA2.2515.14.3 | 0.15 | 0.15 |
| LILRB2.5091.28.3 | 0.36 | 0.36 |
| KRTDAP.5739.75.3 | 0.08 | 0.01 |
| GSTM1.7239.9.3 | 0.05 | 0.02 |
| CFI.2567.5.6 | 0.08 | 0.07 |
| CNTN2.3296.92.2 | 0.22 | 0.22 |
| TPI1.4309.59.3 | 0.14 | 0.02 |
| ATP4B.9994.217.3 | 0.05 | 0.01 |
| GGH.9370.69.3 | 0.12 | 0.11 |
| TCN2.5584.21.3 | 0.65 | 0.65 |
| WISP1.13692.154.3 | 0.25 | 0.25 |
| PLXNA1.9005.16.3 | 0.03 | 0.03 |
| CSGALNACT2.10772.21.3 | 0.02 | 0.02 |
| AZGP1.9312.8.3 | 0.04 | 0.01 |
| TAPBPL.6364.7.3 | 0.76 | 0.56 |
| GPC5.4991.12.1 | 0.25 | 0.25 |
| SERPINF1.9211.19.3 | 0.13 | 0.13 |
| APCS.2474.54.5 | 0.05 | 0.05 |

**Supplementary Table 3**. Robustness analysis.

| **ID** | **bslmm method** | | | | **LD clumped** | | | | ***p*-value set as 5×10^-7^** | | | | **p-value set as 5×10^-9^** | | | | **p-value set as 5×10^-10^** | | | |
| --- | --- | --- | --- | --- | --- | --- | --- | --- | --- | --- | --- | --- | --- | --- | --- | --- | --- | --- | --- | --- |
|  | **MODEL** | **R^2^** | **Z** | **P** | **MODEL** | **R^2^** | **Z** | **P** | **MODEL** | **R^2^** | **Z** | **P** | **MODEL** | **R^2^** | **Z** | **P** | **MODEL** | **R^2^** | **Z** | **P** |
| ABO.9253.52.3 | bslmm | 0.73 | 9.32 | 1.21E-20 | blup | 0.70 | 8.90 | 5.52E-19 | blup | 0.72 | 9.12 | 7.21E-20 | blup | 0.72 | 9.20 | 3.54E-20 | blup | 0.72 | 9.20 | 3.54E-20 |
| CHST15.4469.78.2 | bslmm | 0.04 | -8.92 | 4.80E-19 | enet | 0.05 | -7.91 | 2.66E-15 | lasso | 0.06 | -7.52 | 5.46E-14 | lasso | 0.05 | -8.63 | 6.25E-18 | lasso | 0.05 | -8.63 | 6.30E-18 |
| CD209.3029.52.2 | bslmm | 0.38 | 7.94 | 2.02E-15 | enet | 0.37 | 8.43 | 3.40E-17 | enet | 0.39 | 8.52 | 1.62E-17 | enet | 0.39 | 8.52 | 1.62E-17 | enet | 0.39 | 8.52 | 1.62E-17 |
| GOLM1.8983.7.3 | bslmm | 0.14 | 7.7 | 1.39E-14 | enet | 0.14 | 8.23 | 1.87E-16 | lasso | 0.14 | 8.07 | 7.15E-16 | lasso | 0.14 | 8.07 | 7.04E-16 | lasso | 0.14 | 7.99 | 1.33E-15 |
| ENG.4908.6.1 | bslmm | 0.01 | -3.95 | 7.97E-05 | enet | 0.02 | -4.26 | 2.02E-05 | lasso | 0.01 | -6.26 | 3.92E-10 | top1 | 0.01 | -8.04 | 8.93E-16 | NA | NA | NA | NA |
| CHST15.14097.86.3 | bslmm | 0.04 | -8.8 | 1.40E-18 | enet | 0.05 | -7.05 | 1.74E-12 | lasso | 0.04 | -8.05 | 8.59E-16 | lasso | 0.04 | -8.53 | 1.51E-17 | lasso | 0.04 | -8.53 | 1.52E-17 |
| QSOX2.8397.147.3 | bslmm | 0.39 | 7.68 | 1.53E-14 | enet | 0.37 | 7.65 | 1.98E-14 | enet | 0.40 | 7.98 | 1.44E-15 | enet | 0.40 | 7.97 | 1.62E-15 | enet | 0.40 | 7.97 | 1.62E-15 |
| SELE.3470.1.2 | bslmm | 0.38 | -7.43 | 1.08E-13 | top1 | 0.38 | -7.34 | 2.21E-13 | lasso | 0.39 | -7.88 | 3.33E-15 | lasso | 0.39 | -7.88 | 3.30E-15 | lasso | 0.39 | -7.88 | 3.27E-15 |
| INSR.3448.13.2 | bslmm | 0.09 | -7.32 | 2.43E-13 | enet | 0.09 | -6.87 | 6.52E-12 | enet | 0.10 | -7.23 | 4.87E-13 | lasso | 0.09 | -7.53 | 5.07E-14 | lasso | 0.09 | -7.52 | 5.28E-14 |
| IGF1R.4232.19.2 | bslmm | 0.01 | -6.13 | 8.84E-10 | top1 | 0.01 | -7.39 | 1.42E-13 | enet | 0.04 | -3.00 | 2.68E-03 | NA | NA | NA | NA | NA | NA | NA | NA |
| LIFR.5837.49.3 | bslmm | 0.03 | -6.52 | 7.02E-11 | top1 | 0.03 | -7.39 | 1.42E-13 | lasso | 0.03 | -7.07 | 1.56E-12 | top1 | 0.03 | -7.39 | 1.42E-13 | top1 | 0.03 | -7.39 | 1.42E-13 |
| ALPI.10463.23.3 | bslmm | 0.03 | -4.11 | 3.89E-05 | lasso | 0.04 | -6.62 | 3.56E-11 | lasso | 0.03 | -6.79 | 1.13E-11 | lasso | 0.03 | -6.79 | 1.12E-11 | lasso | 0.03 | -6.75 | 1.44E-11 |
| KDR.3651.50.5 | bslmm | 0.18 | -4.49 | 7.14E-06 | enet | 0.18 | -5.38 | 7.41E-08 | enet | 0.18 | -6.07 | 1.28E-09 | enet | 0.18 | -6.20 | 5.80E-10 | enet | 0.18 | -6.20 | 5.80E-10 |
| FAM3D.13102.1.3 | bslmm | 0.34 | 6.08 | 1.23E-09 | lasso | 0.37 | 5.55 | 2.92E-08 | enet | 0.37 | 6.10 | 1.07E-09 | enet | 0.37 | 6.10 | 1.09E-09 | enet | 0.37 | 6.12 | 9.45E-10 |
| TPST2.8024.64.3 | bslmm | 0.06 | 5.15 | 2.63E-07 | enet | 0.08 | 5.76 | 8.48E-09 | enet | 0.09 | 5.78 | 7.57E-09 | lasso | 0.06 | 6.77 | 1.25E-11 | lasso | 0.06 | 6.77 | 1.25E-11 |
| TIE1.2844.53.2 | bslmm | 0.21 | 4.45 | 8.43E-06 | enet | 0.23 | 5.57 | 2.62E-08 | lasso | 0.22 | 5.67 | 1.45E-08 | lasso | 0.22 | 5.67 | 1.46E-08 | lasso | 0.22 | 5.67 | 1.46E-08 |
| KIN.14643.27.3 | bslmm | 0.05 | -3.84 | 1.25E-04 | lasso | 0.05 | -6.35 | 2.10E-10 | enet | 0.05 | -5.66 | 1.55E-08 | lasso | 0.05 | -6.38 | 1.79E-10 | lasso | 0.05 | -6.39 | 1.66E-10 |
| DSG2.9484.75.3 | bslmm | 0.04 | 6.29 | 3.14E-10 | blup | 0.04 | 4.39 | 1.13E-05 | enet | 0.04 | 5.34 | 9.18E-08 | enet | 0.04 | 5.34 | 9.18E-08 | enet | 0.04 | 5.24 | 1.62E-07 |
| THSD1.5621.64.3 | bslmm | 0.04 | -4.76 | 1.95E-06 | blup | 0.05 | -4.08 | 4.59E-05 | enet | 0.04 | -5.34 | 9.41E-08 | enet | 0.04 | -5.34 | 9.41E-08 | enet | 0.04 | -5.18 | 2.27E-07 |
| FAM20B.7198.197.3 | bslmm | 0.02 | 4.51 | 6.33E-06 | blup | 0.03 | 3.59 | 3.36E-04 | lasso | 0.02 | 5.29 | 1.23E-07 | lasso | 0.02 | 5.44 | 5.32E-08 | lasso | 0.02 | 5.45 | 4.92E-08 |
| MET.2837.3.2 | bslmm | 0.07 | -5.83 | 5.48E-09 | blup | 0.08 | -4.96 | 7.05E-07 | blup | 0.07 | -5.00 | 5.72E-07 | blup | 0.06 | -5.58 | 2.46E-08 | enet | 0.06 | -6.90 | 5.32E-12 |
| B4GALT1.13381.49.3 | bslmm | 0.08 | 3.47 | 5.19E-04 | enet | 0.09 | 4.43 | 9.24E-06 | enet | 0.08 | 4.65 | 3.26E-06 | enet | 0.08 | 4.65 | 3.29E-06 | enet | 0.08 | 4.65 | 3.29E-06 |
| ADGRF5.6409.57.3 | bslmm | 0.44 | -4.89 | 1.01E-06 | lasso | 0.45 | -4.48 | 7.49E-06 | lasso | 0.45 | -4.65 | 3.24E-06 | lasso | 0.45 | -4.65 | 3.25E-06 | lasso | 0.46 | -4.66 | 3.23E-06 |
| CPB1.6356.3.3 | bslmm | 0.04 | -5.73 | 1.01E-08 | lasso | 0.04 | -3.23 | 1.24E-03 | lasso | 0.05 | -3.88 | 1.05E-04 | lasso | 0.04 | -4.54 | 5.55E-06 | lasso | 0.04 | -4.54 | 5.52E-06 |
| CTRB1.5671.1.3 | bslmm | 0.23 | -4.67 | 2.99E-06 | enet | 0.19 | -5.28 | 1.30E-07 | enet | 0.23 | -4.29 | 1.80E-05 | enet | 0.22 | -4.57 | 4.98E-06 | enet | 0.22 | -4.57 | 4.98E-06 |
| GLCE.7808.5.3 | bslmm | 0.36 | 3.95 | 7.77E-05 | enet | 0.36 | 4.25 | 2.13E-05 | lasso | 0.36 | 4.15 | 3.34E-05 | lasso | 0.36 | 4.18 | 2.94E-05 | lasso | 0.36 | 4.18 | 2.96E-05 |
| DPEP2.8327.26.3 | bslmm | 0.04 | -5.14 | 2.74E-07 | enet | 0.06 | -4.36 | 1.32E-05 | enet | 0.06 | -3.75 | 1.80E-04 | enet | 0.06 | -4.01 | 5.97E-05 | enet | 0.06 | -4.01 | 5.97E-05 |
| LAMA1.LAMB1.LAMC1.2728.62.2 | bslmm | 0.07 | 2.77 | 5.54E-03 | enet | 0.09 | 3.74 | 1.87E-04 | enet | 0.08 | 3.88 | 1.06E-04 | enet | 0.08 | 3.88 | 1.06E-04 | enet | 0.08 | 3.88 | 1.06E-04 |
| SELP.4154.57.2 | bslmm | 0.25 | -3.05 | 2.25E-03 | lasso | 0.24 | -4.17 | 2.99E-05 | enet | 0.27 | -3.27 | 1.09E-03 | lasso | 0.26 | -3.77 | 1.66E-04 | lasso | 0.26 | -3.77 | 1.66E-04 |
| IL6ST.2620.4.2 | bslmm | 0.05 | -4.09 | 4.33E-05 | enet | 0.07 | -3.81 | 1.40E-04 | enet | 0.07 | -3.28 | 1.05E-03 | enet | 0.06 | -3.75 | 1.76E-04 | enet | 0.04 | -4.08 | 4.55E-05 |
| SEMA6A.7945.10.3 | bslmm | 0.06 | -1.73 | 8.28E-02 | enet | 0.07 | -3.31 | 9.17E-04 | enet | 0.05 | -3.57 | 3.54E-04 | lasso | 0.05 | -4.40 | 1.07E-05 | lasso | 0.05 | -4.40 | 1.09E-05 |
| IL23R.5088.175.3 | bslmm | 0.03 | 3.54 | 4.06E-04 | enet | 0.04 | 3.54 | 3.95E-04 | enet | 0.04 | 3.55 | 3.80E-04 | enet | 0.04 | 3.55 | 3.80E-04 | enet | 0.04 | 3.55 | 3.80E-04 |
| CHST11.7779.86.3 | bslmm | 0.11 | 1.46 | 1.44E-01 | enet | 0.12 | 4.44 | 8.80E-06 | enet | 0.11 | 3.52 | 4.25E-04 | enet | 0.10 | 3.70 | 2.15E-04 | enet | 0.10 | 3.70 | 2.15E-04 |
| ISLR2.13124.20.3 | bslmm | 0.14 | -2.08 | 3.73E-02 | blup | 0.15 | -2.82 | 4.74E-03 | enet | 0.15 | -3.31 | 9.48E-04 | lasso | 0.14 | -3.18 | 1.49E-03 | lasso | 0.14 | -3.18 | 1.50E-03 |
| LMAN2L.8013.9.3 | bslmm | 0.03 | 3.49 | 4.92E-04 | top1 | 0.03 | 3.35 | 8.01E-04 | lasso | 0.04 | 3.73 | 1.88E-04 | top1 | 0.03 | 3.35 | 8.01E-04 | top1 | 0.03 | 3.35 | 8.01E-04 |
| STOM.8261.51.3 | bslmm | 0.11 | 3.19 | 1.41E-03 | lasso | 0.12 | 3.39 | 7.09E-04 | lasso | 0.11 | 3.31 | 9.21E-04 | top1 | 0.11 | 3.09 | 1.98E-03 | top1 | 0.11 | 3.09 | 1.98E-03 |
| CD36.2973.15.2 | bslmm | 0.03 | 2.91 | 3.57E-03 | enet | 0.04 | NA | NA | top1 | 0.03 | 3.31 | 9.25E-04 | top1 | 0.03 | 3.31 | 9.25E-04 | top1 | 0.03 | 3.31 | 9.25E-04 |
| NOTCH1.5107.7.2 | bslmm | 0.01 | 2.91 | 3.65E-03 | blup | 0.01 | 1.78 | 7.52E-02 | top1 | 0.01 | 3.29 | 9.97E-04 | top1 | 0.01 | 3.29 | 9.97E-04 | NA | NA | NA | NA |
| ADH1B.9834.62.3 | bslmm | 0.07 | 3.02 | 2.56E-03 | lasso | 0.08 | 3.19 | 1.40E-03 | enet | 0.08 | 3.31 | 9.32E-04 | enet | 0.08 | 3.13 | 1.77E-03 | enet | 0.08 | 2.99 | 2.76E-03 |
| LRPAP1.3640.14.3 | NA | NA | NA | NA | enet | 0.25 | 2.90 | 3.70E-03 | enet | 0.27 | 3.00 | 2.68E-03 | enet | 0.26 | 3.29 | 1.01E-03 | enet | 0.27 | 3.26 | 1.12E-03 |

**Supplementary Table 4.** Somatic level potentially deleterious changes of genes encoding identified proteins in TCGA pancreatic adenocarcinoma patients.

| **Gene** | **Chr** | **Start Position** | **End Position** | **Variant Classification** | **Reference Allele** | **Alternative Allele** | **HGVSp annotation** | **Frequency (%)** |
| --- | --- | --- | --- | --- | --- | --- | --- | --- |
| LAMA1 | chr18 | 6,943,189 | 6,943,189 | Missense Mutation | C | T | p.Gly3020Ser | 1/167 (0.60%) |
| IL6ST | chr5 | 55,951,928 | 55,951,928 | Splice Site | C | A | p.X567_splice | 1/167 (0.60%) |
| DPEP2 | chr16 | 67,990,882 | 67,990,882 | Missense Mutation | G | A | p.Ser283Leu | 1/167 (0.60%) |
| CHST11 | chr12 | 104,756,986 | 104,756,986 | Missense Mutation | G | T | p.Arg81Leu | 1/167 (0.60%) |
| TIE1 | chr1 | 43,313,239 | 43,313,239 | Missense Mutation | G | A | p.Val678Ile | 1/167 (0.60%) |
| GLCE | chr15 | 69,261,147 | 69,261,147 | Missense Mutation | A | C | p.Gln216Pro | 1/167 (0.60%) |
| ISLR2 | chr15 | 74,134,243 | 74,134,243 | Missense Mutation | G | A | p.Glu497Lys | 1/167 (0.60%) |
| LMAN2L | chr2 | 96,734,471 | 96,734,471 | Missense Mutation | C | G | p.Gly121Ala | 1/167 (0.60%) |
| LRPAP1 | chr4 | 3,514,849 | 3,514,855 | Frame Shift Del | AGCTTCT | - | p.Glu303GlyfsTer13 | 1/167 (0.60%) |
| CPB1 | chr3 | 148,845,572 | 148,845,572 | Missense Mutation | G | A | p.Met309Ile | 1/167 (0.60%) |
| INSR | chr19 | 7,117,078 | 7,117,078 | Missense Mutation | A | C | p.Leu1376Trp | 1/167 (0.60%) |
| IL23R | chr1 | 67,240,182 | 67,240,182 | Missense Mutation | A | G | p.Asn350Ser | 1/167 (0.60%) |
| SELP | chr1 | 169,609,594 | 169,609,594 | Missense Mutation | G | A | p.Arg415Cys | 1/167 (0.60%) |
| SELE | chr1 | 169,725,910 | 169,725,910 | Missense Mutation | G | A | p.Ala591Val | 1/167 (0.60%) |
| ALPI | chr2 | 232,456,604 | 232,456,604 | Missense Mutation | C | T | p.Ala70Val | 1/167 (0.60%) |
| FAM3D | chr3 | 58,637,154 | 58,637,154 | Missense Mutation | C | T | p.Asp149Asn | 1/167 (0.60%) |
| KDR | chr4 | 55,121,154 | 55,121,154 | Missense Mutation | C | A | p.Ser35Ile | 1/167 (0.60%) |
| ADH1B | chr4 | 99,310,834 | 99,310,834 | Missense Mutation | G | A | p.Ala345Val | 1/167 (0.60%) |
| LIFR | chr5 | 38,484,781 | 38,484,781 | Missense Mutation | C | T | p.Arg862Gln | 1/167 (0.60%) |
| SEMA6A | chr5 | 116,478,116 | 116,478,116 | Missense Mutation | C | T | p.Gly489Asp | 1/167 (0.60%) |
| ADGRF5 | chr6 | 46,884,158 | 46,884,158 | Missense Mutation | C | A | p.Gly148Trp | 1/167 (0.60%) |
| MET | chr7 | 116,699,436 | 116,699,436 | Missense Mutation | A | C | p.Met118Leu | 1/167 (0.60%) |
| ENG | chr9 | 127,819,658 | 127,819,658 | Splice Region | C | T | p.Ala425= | 1/167 (0.60%) |
| ABO | chr9 | 133,257,476 | 133,257,476 | Missense Mutation | C | T | p.Asp102Asn | 1/167 (0.60%) |
| QSOX2 | chr9 | 136,209,108 | 136,209,108 | Missense Mutation | C | T | p.Ala573Thr | 1/167 (0.60%) |
| NOTCH1 | chr9 | 136,497,438 | 136,497,438 | Missense Mutation | C | T | p.Ala2101Thr | 1/167 (0.60%) |
| THSD1 | chr13 | 52,377,932 | 52,377,932 | Missense Mutation | C | T | p.Ala680Thr | 1/167 (0.60%) |
| IGF1R | chr15 | 98,922,350 | 98,922,350 | Missense Mutation | G | A | p.Asp802Asn | 1/167 (0.60%) |
| DSG2 | chr18 | 31,535,398 | 31,535,398 | Missense Mutation | T | C | p.Val470Ala | 1/167 (0.60%) |

**Supplementary Table 5**. Top diseases, bio functions, and networks associated with the genes encoding identified pancreatic cancer risk associated proteins.

| **Top diseases and disorders** | | **Molecular and Cellular Functions** | | **Top networks** | |
| --- | --- | --- | --- | --- | --- |
| **Terms** | ***P*-value** | **Terms** | ***P*-value** | **Terms** | **Score** |
| Cancer | 2.88×10^-5^ | Cellular Function and Maintenance | 1.54×10^-4^ | Cell-To-Cell Signaling and Interaction, Cardiovascular System Development and Function, Organismal Developmen | 25 |
| Organismal Injury and Abnormalities | 2.88×10^-5^ | Cellular Movement | 1.54×10^-4^ | Cancer, Organismal Injury and Abnormalities, Respiratory Disease | 12 |
| Respiratory Disease | 2.88×10^-5^ | Cell-To-Cell Signaling and Interaction | 2.60×10^-4^ | Free Radical Scavenging, Cell Death and Survival, Organismal Injury and Abnormalities | 17 |
| Tumor Morphology | 5.36×10^-5^ | Carbohydrate Metabolism | 2.76×10^-4^ | Carbohydrate Metabolism, Small Molecule Biochemistry, Cell Cycle | 12 |
| Inflammatory Response | 2.36×10^-4^ | Cell Death and Survival | 4.88×10^-4^ | Cancer, Cell-To-Cell Signaling and Interaction, Cellular Assembly and Organization | 7 |

**Reference**

1. Zhu J, Shu X, Guo X, Liu D, Bao J, Milne RL*, et al.* Associations between Genetically Predicted Blood Protein Biomarkers and Pancreatic Cancer Risk. Cancer Epidemiol Biomarkers Prev **2020**;29(7):1501-8 doi 10.1158/1055-9965.EPI-20-0091.
