# Supplementary material for: Proteome-wide association study and functional validation identify novel protein markers for pancreatic ductal adenocarcinoma
Source: Gigascience. 2024 Apr 12;13:giae012. doi: 10.1093/gigascience/giae012 (PMC11010651; doi:10.1093/gigascience/giae012)
Supplement: giae012_Response_to_Reviewer_Comments_Original_Submission [file giae012_response_to_reviewer_comments_original_submission.pdf]

Author's Response To Reviewer Comments

Re: GIGA-D-23-00321

Proteome-wide association study and functional validation identify novel protein markers for pancreatic ductal adenocarcinoma

Authors' responses to reviewers (Page and line numbers in our responses refer to the revised version of the manuscript with TRACK CHANGES)

Reviewer #1:

Proteome-Wide Association Study (PWAS) marks a significant advancement in biomedical research, bears great potential in identifying protein biomarkers linked to cancer's onset, progression, and treatment response, which are crucial for early detection, diagnosis, and monitoring. In the present study, Jingjing et al. leverage genome and plasma proteome data from 2,481 healthy individuals of European descent from the INTERVAL study to develop protein genetic prediction models. Their PWAS investigation, using these models, aims to identify potential protein markers for cancer. They notably pinpoint two novel proteomic markers, GOLM1 and B4GALT1, that may significantly influence pancreatic ductal adenocarcinoma cell behaviors.

In general, this pioneering PWAS work in exploring genetically predicted blood protein concentrations and their association with PDAC risk is undeniably a breakthrough in cancer research. However, the second part of this study, namely the process used to screen out GOLMA1 and B4GALT1 raised some questions and concerns.

Specifically In the words from 364 to line 367. The authors claimed that "Among the 16 novel associated proteins, analysis of TGCA data also revealed potential relevance of B4GT1 and GOLM1 with tumor development (data not shown). Consequently, these two proteins were selected as the targets for experimental validation to further investigate their potential roles in PDAC development." I don't understand why they addressed "data not shown". The absence of this crucial data and the rationale for prioritizing these two proteins over other 14 proteins are not clear. This omission is particularly concerning as neither B4GT1 nor GOLM1 is listed in Supplementary Table 2 as having relevant somatic mutations using TCGA data.

Response-1:
Thank you very much for your insightful comments and suggestions concerning our paper. We agree that these points are pivotal for understanding the unique significance of B4GT1 and GOLM1. Please allow us to provide further information to clarify these issues.

Regarding your point on "data not shown", to substantiate our selection of B4GT1 and GOLM1, we have now included the analysis result of TCGA data as supplementary figures (Supplementary Fig. 2 and 3). In brief, we have conducted a comprehensive bioinformatic analysis leveraging data from TCGA, which clearly indicated the potential relevance of B4GALT1 and GOLM1 with pancreatic tumor development. We apologize for the omission in the previous version of the manuscript.

Page 12, Lines 274-286:
Gene Expression and Survival Analysis with TCGA Database
The examination of GOLM1 and B4GALT1 gene expressions in Pancreatic Adenocarcinoma (PAAD) was conducted using GEPIA (Gene Expression Profiling Interactive Analysis). The platform, accessible at the following web link: http://gepia.cancer-pku.cn/, facilitated analysis with a dataset consisting of 179 tumor samples and 171 normal controls. The focus of survival analysis was exclusively on PAAD, leveraging TCGA data through the GEPIA web server.
Customized gene selection, normalization, and survival methodologies were implemented to suit the unique

characteristics of PAAD. Cohort thresholds were defined, restricting dataset selection to PAAD, and survival plots were generated. These measures were designed to precisely identify the correlation between gene expression and survival outcomes specific to this type of cancer.

Page 18, Lines 423-439:
Among the 16 novel associated proteins, analysis of TGCA data also revealed potential relevance of B4GT1 and GOLM1 with tumor development (Supplementary Figure 2 and 3). The examination of GOLM1 and B4GALT1 gene expression in PADD cancer was conducted using GEPIA (Gene Expression Profiling Interactive Analysis). The analysis involved a dataset consisting of 179 tumor samples and 171 normal controls. The box plot analysis revealed a statistically significant increase in GOLM1 (Supplementary Figure 2A) and B4GALT1 (Supplementary Figure 3A) expression in the tumor samples as compared with the normal control group. GEPIA, accessible through the following web link: http://gepia.cancer-pku.cn/, served as the platform for this investigation. The survival analysis of GOLM1 and B4GALT1 gene expression in PADD cancer was conducted using GEPIA. Survival plots revealed a significant decrease in overall survival (OS) and disease-free survival (DFS) among tumor samples exhibiting elevated GOLM1 or B4GALT1 expression (n=89) compared with those with low expression (n=89). Employing the Log-rank test for hypothesis testing, our findings emphasize a noteworthy correlation between heightened gene expression and reduced OS and DFS in the PADD cancer cohort (Supplementary Figure 2B, C, Supplementary Figure 3B, C).

I could understand that due to the novelty of PWAS, the authors are able to successfully identified B4GT1 and GOLM1 as important markers at proteomic level. However, through literature search, there is very limited published peer-reviewed papers to show them play any roles in Pancreatic ductal adenocarcinoma in other omics level, like genetics, genomics, transcriptomics.
Response-2:
Thanks for your comment. Your statement underlines a relevant point about the yet unclear roles of B4GT1 and GOLM1 at other omics levels in pancreatic ductal adenocarcinoma. We think that this indeed underscores the potential of our innovative PWAS design in uncovering novel proteins that could not have been identified if we use another design focusing on other omics level. As described above in another response, after we identified these two proteins, when we focused on their RNA expression levels, we could identify additional evidence at RNA levels showing their potential relevance with PDAC.

Were the other 14 proteins subjected to similar experimental protocols, and if so, what were the findings? This information is vital for understanding the unique significance of B4GT1 and GOLM1 in this context.
Response-3:
Thanks for your comment. We conducted a bioinformatics analysis using the GEPIA online TCGA tool to investigate the survival rates associated with the expression of the 16 genes encoding the novel proteins with genetically predicted concentrations in plasma linked to PDAC risk. The findings indicate that, in pancreatic adenocarcinoma (PAAD), GOLM1, B4GALT1, FAM20B, FAB3D, and LRPAP1 exhibit significantly higher expression in tumor tissues, and they are associated with noteworthy survival rate differences among patients. Further validation through mRNA PCR tests in normal Human Pancreatic Duct Epithelial Cell Line and pancreatic cancer cell lines (PANC-1, SU.86.86) revealed that only GOLM1 and B4GALT1 displayed elevated expression in pancreatic cancer cell lines. Consequently, for subsequent biological investigations, GOLM1 and B4GALT1 were selected due to their distinct high expression in pancreatic cancer cell lines, suggesting their potential relevance to the pathogenesis of pancreatic cancer.

Experimental studies to validate the role of all 16 novel proteins would be exhaustive in terms of resources and time. Given the supportive associations of B4GALT1 and GOLM1 revealed by the TCGA data, it was prudent to prioritize these two for experimental validation, in the current stage of study. We believe this maybe the most efficient strategy to follow up on a large number of candidates generated from a high-throughput PWAS, but agree that the other 14 proteins certainly warrant further investigation.

Finally, concerning the other 14 proteins, although they were not subjected to the same experimental protocols, ongoing studies in our lab are focused on further analyzing these proteins in vitro and in vivo to better understand their roles in PDAC. As these studies were not included in the current manuscript, we

would be delighted to share our findings in an appropriate future publication.

We hope these explanations address your concerns, and we thank you again for improving the quality of our work through your insightful comments.

Reviewer #2:

Zhu et al. constructed a series of pQTL models and used them to identify genetic predicted serum protein markers for pancreatic ductal adenocarcinoma, followed by a series of functional validations, which may provide valuable clues for prediction and treatment of PDAC. I have several concerns on this study.

Major concerns:
1. This study integrated both cis- and trans-acting elements to construct pQTL models. It would be better to provide the heritability of each pQTL model constructed and the comparison results (such as the h2 explained and predictive performance on gene expression) with those focus solely on cis-acting variants, as the author stated that the integration strategy has an enhanced statistical power.

Rsponse-1:
Thank you very much for your insightful comments. We have compared h2 of the prediction models between those with cis+trans factors and only cis genetic factors. The results indeed showed that when involving trans-acting elements, enhanced statistical power could be achieved.

Page 8, Lines 181-185:
We also estimated the genetic heritability of plasma proteins (the proportion of the variation of protein levels that could be explained by potential predictors) using GCTA1. We compared the heritability of plasma proteins when using cis+trans SNPs vs only cis SNPs to assess whether it could capture more heritability when involving trans-SNPs.

Page 16, Lines 376-383:
We compared the heritability of the prediction models established using cis+trans and vs cis-only predictors strategies. Here, we focused on the 490 models established using both cis and trans SNPs in the main analysis. The results showed that 250 out of the 490 (51.02%) models have higher estimated heritability with the cis+trans strategy (Supplementary Table 2), and 215 proteins (43.88%) showed the same estimated heritability between cis+trans and cis-only strategies (Supplementary Table 2). Only 25 proteins (5.10%) showed lower estimated heritability when using cis+trans strategy (Supplementary Table 2). These results showed that trans SNPs could in general increase heritability of the prediction models.

2. The integration strategy is somewhat like some PGS methods (such as C+T). Would the author consider to try some other strategies used in common PGS analysis? For example, using LD clumping for SNPs selection, trying some other P value threshold combinations to define and select gene- associated SNPs in cis and trans regions, and using the bslmm strategy, which seems to be demonstrated to have decent performance in the FUSION article.
Rsponse-2:
We thank the reviewer for the comments. We have now performed several additional robustness analyses, including using the bslmm method, LD clumping for SNP selection, and different p-value thresholds. The results show that our results are robust under different methods/thresholds.

Page 10, Lines 220-233:
Robustness analyses
To further examine whether the identified significant associations from the main analyses may be robust to different strategies, three alternative strategies were used to test these proteins under different scenorios. Firstly, we established prediction models using the bslmm method embedded in TWAS/FUSION software. This method was not enabled by the default parameter due to the intensive Markov chain Monte Carlo

(MCMC) computation, although bslmm has some advantages and might increase prediction accuracy in some conditions. Secondly, we pruned the highly correlated SNPs and only SNPs that are weakly correlated with each other were used as potential predictors. In the current analysis, we pruned SNPs using pruning parameters r2 = 0.1 and distance = 250 kb. Thirdly, we assessed the robustness of the significant association results by examining different p-value cutoffs for selecting informative trans-regions (p-value < 5×10-7, p-value < 5×10-9, and p-value < 5×10-10) as candidate predictors for model building. The association results with a nominal p-value < 0.05 and consistent effect direction were considered to be replicated.

Page 16, Lines 384-393:
The robustness analysis showed that all the 40 significantly PDAC-associated proteins had the same effect directions (Supplementary Table 3). A total of 39 proteins could be tested using the bslmm method and 37 out of 39 (94.87%) could be replicated (except for SEMA6A and CHST11 proteins). When we removed highly correlated SNPs and only weak correlated SNPs were used for establishing prediction models, a total of 39 prediction models were established. The association results showed that associations of 38 out of the 39 (97.44%) proteins could be replicated (Supplementary Table 3). In addition, three different p-value thresholds (p-value < 5×10-7, p-value < 5×10-9, and p-value < 5×10-10) for selecting trans-SNPs were examined (Supplementary Table 3). All the association results were consistent with those in our main analysis. The above results showed the robustness of our main results.

3. This study selected proteins for pWAS analysis based on prediction R/R2 of pQTL models. Would the author take the h2 of each pQTL model into consideration as the FUSION article did?
Rsponse-3:
We thank the reviewer for the comments. The R2≥0.01was a common threshold used in previous relevant omics integration studies. Here we also added the information of h2 estimated using the GCTA software in the revised manuscript (main text as well as Tables 1 and 2) 1.

Page 8, Lines 174-175:
R2≥0.01 was used as the threshold for selecting satisfactory prediction models, which is commonly used in relevant omics integration studies.
Page 15, Lines 361-362:
The heritability of the proteins ranged from 0.001 to 0.87, with an average value of 0.14.

4. Although the author used the TWAS/FUSION framework for pQTL models construction and protein-PDAC association assessment, it would be better to add more description into the supplementary file on how this framework was applied to the current study.
Rsponse-4:
We thank the reviewer for the comments. We have now added more descriptions of the way we performed the association assessment.

Page 9, Lines 212-216:
We calculated the PWAS test statistic Z-score = w'Z/(w'Σs,sw)1/2, where the Z is a vector of standardized effect sizes of SNPs for a given protein (Wald z-scores), w is a vector of prediction weights for the abundance feature of the protein being tested, and the Σs,s is the LD matrix of the SNPs estimated from the 1000 Genomes Project as the LD reference panel.

Reference
1. Yang, J., Lee, S. H., Goddard, M. E. & Visscher, P. M. GCTA: a tool for genome-wide complex trait analysis. Am J Hum Genet 88, 76–82 (2011).
