# Supplementary material for: Proteome-wide association study and functional validation identify novel protein markers for pancreatic ductal adenocarcinoma
Source: Gigascience. 2024 Apr 12;13:giae012. doi: 10.1093/gigascience/giae012 (PMC11010651; doi:10.1093/gigascience/giae012)
Supplement: giae012_Reviewer_1_Report_Original_Submission — Jian Sang -- 12/18/2023 Reviewed [file giae012_reviewer_1_report_original_submission.pdf]

Reviewer Report

Title: Proteome-wide association study and functional validation identify novel protein markers for pancreatic ductal adenocarcinoma

Version: Original Submission **Date:** 12/18/2023

Reviewer name: Jian Sang

Reviewer Comments to Author:

Proteome-Wide Association Study (PWAS) marks a significant advancement in biomedical research, bears great potential in identifying protein biomarkers linked to cancer's onset, progression, and treatment response, which are crucial for early detection, diagnosis, and monitoring. In the present study, Jingjing et al. leverage genome and plasma proteome data from 2,481 healthy individuals of European descent from the INTERVAL study to develop protein genetic prediction models. Their PWAS investigation, using these models, aims to identify potential protein markers for cancer. They notably pinpoint two novel proteomic markers, GOLM1 and B4GALT1, that may significantly influence pancreatic ductal adenocarcinoma cell behaviors.

In general, this pioneering PWAS work in exploring genetically predicted blood protein concentrations and their association with PDAC risk is undeniably a breakthrough in cancer research. However, the second part of this study, namely the process used to screen out GOLMA1 and B4GALT1 raised some questions and concerns.

Specifically In the words from 364 to line 367. The authors claimed that "Among the 16 novel associated proteins, analysis of TGCA data also revealed potential relevance of B4GT1 and GOLM1 with tumor development (data not shown). Consequently, these two proteins were selected as the targets for experimental validation to further investigate their potential roles in PDAC development." I don't understand why they addressed "data not shown". The absence of this crucial data and the rationale for prioritizing these two proteins over other 14 proteins are not clear. This omission is particularly concerning as neither B4GT1 nor GOLM1 is listed in Supplementary Table 2 as having relevant somatic mutations using TCGA data.

I could understand that due to the novelty of PWAS, the authors are able to successfully identified B4GT1 and GOLM1 as important markers at proteomic level. However, through literature search, there is very limited published peer-reviewed papers to show them play any roles in Pancreatic ductal adenocarcinoma in other omics level, like genetics, genomics, transcriptomics.

Were the other 14 proteins subjected to similar experimental protocols, and if so, what were the findings? This information is vital for understanding the unique significance of B4GT1 and GOLM1 in this context.

Methods

Are the methods appropriate to the aims of the study, are they well described, and are necessary controls included? Choose an item.

Conclusions

Are the conclusions adequately supported by the data shown? Choose an item.

Reporting Standards

Does the manuscript adhere to the journal's guidelines on minimum standards of reporting? Choose an item.

Choose an item.

Statistics

Are you able to assess all statistics in the manuscript, including the appropriateness of statistical tests used? Choose an item.

Quality of Written English

Please indicate the quality of language in the manuscript: Choose an item.

Declaration of Competing Interests

Please complete a declaration of competing interests, considering the following questions:

- Have you in the past five years received reimbursements, fees, funding, or salary from an organisation that may in any way gain or lose financially from the publication of this manuscript, either now or in the future?
- Do you hold any stocks or shares in an organisation that may in any way gain or lose financially from the publication of this manuscript, either now or in the future?
- Do you hold or are you currently applying for any patents relating to the content of the manuscript?
- Have you received reimbursements, fees, funding, or salary from an organization that holds or has applied for patents relating to the content of the manuscript?
- Do you have any other financial competing interests?
- Do you have any non-financial competing interests in relation to this paper?

If you can answer no to all of the above, write 'I declare that I have no competing interests' below. If your reply is yes to any, please give details below.

I declare that I have no competing interests

I agree to the open peer review policy of the journal. I understand that my name will be included on my report to the authors and, if the manuscript is accepted for publication, my named report including any attachments I upload will be posted on the website along with the authors' responses. I agree for my

report to be made available under an Open Access Creative Commons CC-BY license (http://creativecommons.org/licenses/by/4.0/). I understand that any comments which I do not wish to be included in my named report can be included as confidential comments to the editors, which will not be published.

Choose an item.

To further support our reviewers, we have joined with Publons, where you can gain additional credit to further highlight your hard work (see: https://publons.com/journal/530/gigascience). On publication of this paper, your review will be automatically added to Publons, you can then choose whether or not to claim your Publons credit. I understand this statement.

Yes Choose an item.
