# Supplementary material for: Proteome-wide association study and functional validation identify novel protein markers for pancreatic ductal adenocarcinoma
Source: Gigascience. 2024 Apr 12;13:giae012. doi: 10.1093/gigascience/giae012 (PMC11010651; doi:10.1093/gigascience/giae012)
Supplement: giae012_Reviewer_2_Report_Original_Submission — Huang Peng -- 1/2/2024 Reviewed [file giae012_reviewer_2_report_original_submission.pdf]

Reviewer Report

Title: Proteome-wide association study and functional validation identify novel protein markers for pancreatic ductal adenocarcinoma

Version: Original Submission **Date:** 1/2/2024

Reviewer name: Huang Peng

Reviewer Comments to Author:

Zhu et al. constructed a series of pQTL models and used them to identify genetic predicted serum protein markers for pancreatic ductal adenocarcinoma, followed by a series of functional validations, which may provide valuable clues for prediction and treatment of PDAC. I have several concerns on this study.

Major concerns:

1. This study integrated both cis- and trans-acting elements to construct pQTL models. It would be better to provide the heritability of each pQTL model constructed and the comparison results (such as the h2 explained and predictive performance on gene expression) with those focus solely on cis-acting variants, as the author stated that the integration strategy has an enhanced statistical power.
2. The integration strategy is somewhat like some PGS methods (such as C+T). Would the author consider to try some other strategies used in common PGS analysis? For example, using LD clumping for SNPs selection, trying some other P value threshold combinations to define and select gene- associated SNPs in cis and trans regions, and using the bslmm strategy, which seems to be demonstrated to have decent performance in the FUSION article.
3. This study selected proteins for pWAS analysis based on prediction R/R2 of pQTL models. Would the author take the h2 of each pQTL model into consideration as the FUSION article did?
4. Although the author used the TWAS/FUSION framework for pQTL models construction and protein-PDAC association assessment, it would be better to add more description into the supplementary file on how this framework was applied to the current study.

Methods

Are the methods appropriate to the aims of the study, are they well described, and are necessary controls included? Choose an item.

Conclusions

Are the conclusions adequately supported by the data shown? Choose an item.

Reporting Standards

Does the manuscript adhere to the journal's guidelines on [minimum standards of reporting?] Choose an item.

Choose an item.

Statistics

Are you able to assess all statistics in the manuscript, including the appropriateness of statistical tests used? Choose an item.

Quality of Written English

Please indicate the quality of language in the manuscript: Choose an item.

Declaration of Competing Interests

Please complete a declaration of competing interests, considering the following questions:

- Have you in the past five years received reimbursements, fees, funding, or salary from an organisation that may in any way gain or lose financially from the publication of this manuscript, either now or in the future?
- Do you hold any stocks or shares in an organisation that may in any way gain or lose financially from the publication of this manuscript, either now or in the future?
- Do you hold or are you currently applying for any patents relating to the content of the manuscript?
- Have you received reimbursements, fees, funding, or salary from an organization that holds or has applied for patents relating to the content of the manuscript?
- Do you have any other financial competing interests?
- Do you have any non-financial competing interests in relation to this paper?

If you can answer no to all of the above, write 'I declare that I have no competing interests' below. If your reply is yes to any, please give details below.

I declare that I have no competing interests

I agree to the open peer review policy of the journal. I understand that my name will be included on my report to the authors and, if the manuscript is accepted for publication, my named report including any attachments I upload will be posted on the website along with the authors' responses. I agree for my report to be made available under an Open Access Creative Commons CC-BY license (http://creativecommons.org/licenses/by/4.0/). I understand that any comments which I do not wish to be included in my named report can be included as confidential comments to the editors, which will not be published.

Choose an item.

To further support our reviewers, we have joined with Publons, where you can gain additional credit to further highlight your hard work (see: https://publons.com/journal/530/gigascience). On publication of this paper, your review will be automatically added to Publons, you can then choose whether or not to claim your Publons credit. I understand this statement.

Yes Choose an item.
